# Structure-dependent absorption of atypical sphingoid long-chain bases from digestive tract into lymph

**DOI:** 10.1186/s12944-021-01448-2

**Published:** 2021-03-01

**Authors:** Daisuke Mikami, Shota Sakai, Megumi Nishimukai, Kohei Yuyama, Katsuyuki Mukai, Yasuyuki Igarashi

**Affiliations:** 1 Laboratory of Biomembrane and Biofunctional Chemistry, Graduate School of Advanced Life Science, and Frontier Research Center for Post-Genome Science and Technology, Hokkaido University, Kita-21 Nishi-11, Kita-ku, Sapporo, 001-0021 Japan; 2 Department of Biochemistry & Cell Biology, National Institute of Infectious Diseases, 1-23-1 Toyama, Shinjuku-ku, Tokyo, 162-8640 Japan; 3 Department of Animal Science, Faculty of Agriculture, Iwate University, 3-18-8 Ueda, Morioka, Iwate 020-8550 Japan; 4 R & D Headquarters, Daicel Corporation, 2-18-1, Konan, Minato-ku, Tokyo, 108-8230 Japan

**Keywords:** Sphingolipids, Long-chain base, Lipid metabolism, Lipidomics, Lymphatic absorption, Sphingomyelin

## Abstract

**Background:**

Dietary sphingolipids have various biofunctions, including skin barrier improvement and anti-inflammatory and anti-carcinoma properties. Long-chain bases (LCBs), the essential backbones of sphingolipids, are expected to be important for these bioactivities, and they vary structurally between species. Given these findings, however, the absorption dynamics of each LCB remain unclear.

**Methods:**

In this study, five structurally different LCBs were prepared from glucosylceramides (GlcCers) with LCB 18:2(4E,8Z);2OH and LCB 18:2(4E,8E);2OH moieties derived from konjac tuber (*Amorphophallus konjac*), from GlcCers with an LCB 18(9Me):2(4E,8E);2OH moiety derived from Tamogi mushroom (*Pleurotus cornucopiae var. citrinopileatus*), and from ceramide 2-aminoethyphosphonate with LCB 18:3(4E,8E,10E);2OH moiety and LCB 18(9Me):3(4E,8E,10E);2OH moiety derived from giant scallop (*Mizuhopecten yessoensis*), and their absorption percentages and metabolite levels were analyzed using a lymph-duct-cannulated rat model via liquid chromatography tandem mass spectrometry (LC/MS/MS) with a multistage fragmentation method.

**Results:**

The five orally administered LCBs were absorbed and detected in chyle (lipid-containing lymph) as LCBs and several metabolites including ceramides, hexosylceramides, and sphingomyelins. The absorption percentages of LCBs were 0.10–1.17%, depending on their structure. The absorption percentage of LCB 18:2(4E,8Z);2OH was the highest (1.17%), whereas that of LCB 18:3(4E,8E,10E);2OH was the lowest (0.10%). The amount of sphingomyelin with an LCB 18:2(4E,8Z);2OH moiety in chyle was particularly higher than sphingomyelins with other LCB moieties.

**Conclusions:**

Structural differences among LCBs, particularly geometric isomerism at the C8–C9 position, significantly affected the absorption percentages and ratio of metabolites. This is the first report to elucidate that the absorption and metabolism of sphingolipids are dependent on their LCB structure. These results could be used to develop functional foods that are more readily absorbed.

**Supplementary Information:**

The online version contains supplementary material available at 10.1186/s12944-021-01448-2.

## Background

**Fig. 1:**
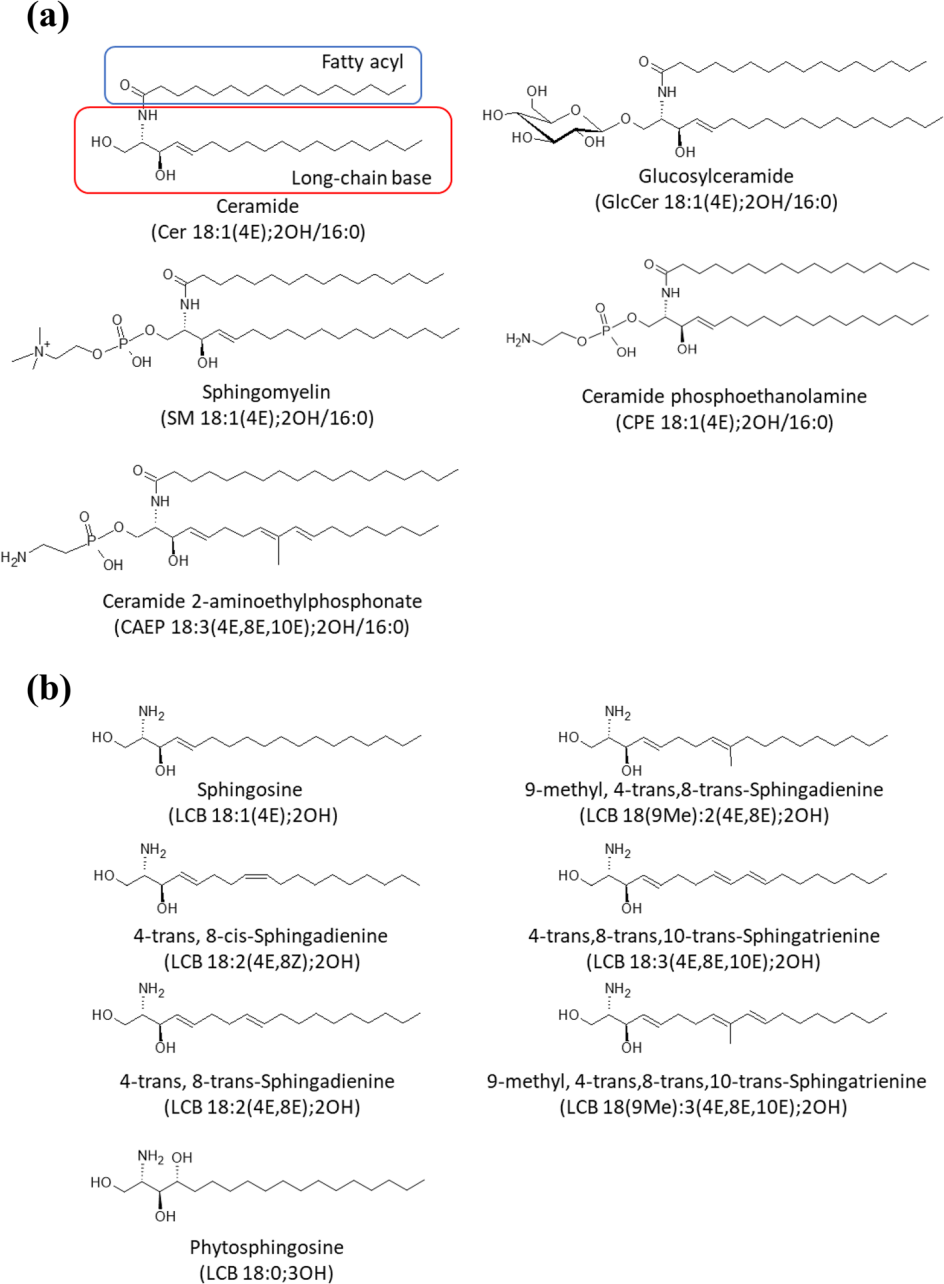
Structures of ceramide, glucosylceramide, sphingomyelin, ceramide phosphoethanolamine, ceramide 2-aminoethylphosphonate (**a**), and long-chain bases (**b**). Structures were drawn using ACD/ChemSketch software

Sphingolipids have long-chain aliphatic amino alcohol moieties, called long-chain bases (LCBs). LCBs are widely distributed in nature and perform important functions in eukaryotic cells [1, 2]. Ceramides consist of LCB and fatty acid (FA) components, and the basic structures of glycosphingolipids and sphingomyelins (SMs) include polar head group sugars and phosphocholine, respectively [3] (Fig. 1a). Sphingolipids are involved in mammalian cell apoptosis, differentiation, proliferation, and cell migration [2–7]. Ingested sphingolipids can induce important physiological activities, such as improvement of skin barrier function, anti-inflammatory action, and suppression of the onset of colorectal cancer [4, 8–11]. Therefore, sphingolipids are speculated to be useful functional food components.

The structures of LCBs vary between species [1] (Fig. 1b). Sphingosine [LCB 18:1(4E);2OH, 2-amino-4-trans-octadecene-1,3-diol] is the most abundant LCB in mammals [1]. Plants and fungi produce LCBs with two double bonds, such as sphingadienine [4-trans,8-cis-sphingadienine, LCB 18:2(4E,8Z);2OH], 4-trans,8-trans-sphingadienine [LCB 18:2(4E,8E);2OH], and 9-methyl,4-trans,8-trans-sphingadienine [LCB 18(9Me):2(4E,8Z);2OH]. Additionally, 4-trans,8-trans,10-trans-sphingatrienine [LCB 18:3(4E,8E,10E);2OH] and 9-methyl,4-trans,8-trans,10-trans-sphingatrienine [LCB 18(9Me):3(4E,8E,10E);2OH], having three double bonds, are found in marine organisms [1]. Phytosphingosine [LCB 18:0;3OH], having three hydroxy groups, are also found in mammal and other species [1].

Orally ingested glucosylceramides (GlcCers) and SMs with an LCB 18:1(4E);2OH moiety are degraded to ceramide and polar head groups by specific enzymes in the digestive tract of rats, which is followed by the breakdown of ceramide to LCB and fatty acids [12–17]. Previous studies have shown that maize-derived GlcCers and squid-derived ceramide 2-aminoethyphosphonate [CAEP, sphingolipid with one oxygen atom missing from ceramide phosphoethanolamine (CPE)] (Fig. 1a), which have atypical LCBs such as LCB 18:2(4E,8E);2OH and LCB 18(9Me):3(4E,8E,10E);2OH, are also hydrolyzed in the digestive tract, and the liberated LCBs are transferred to chyle as LCBs and ceramides with an LCB 18:2(4E,8E);2OH or LCB 18(9Me):3(4E,8E,10E);2OH moiety [18–21]. These findings suggested that liberated LCBs are absorbed into small intestinal epithelial cells, metabolized to sphingolipids from incorporated LCBs in the form of ceramide, and then absorbed into chyle in rats [18, 22, 23]. As the structures of LCBs in plants, fungi, and marine organisms are different from those in mammals [1], the absorption percentages of different LCBs into chyle are likely to differ, depending on their structure. Previous study showed that the absorption percentage of typical LCB 18:1(4E);2OH is estimated to about 0.73%, and it is higher absorption percentage than that of plant derived LCB 18:2(4,8);2OH [19].

Most of the sphingolipid materials currently available on the market are plants or mushrooms, such as konjac, rice and mushrooms. Thus, to elucidate the bioactivity and mechanism of each dietary sphingolipid, it is important to clarify the relationship between their individual bioavailability and structure. Using conventional mass spectrometry (MS) analysis methods, one can analyze the LCB backbones of ceramides and hexosylceramides (HexCers) [24]. In addition, LCB structure of SMs were analyzed by in-source CID/PRM at negative ion mode. To explore the relationship between LCB structures and absorption rates, as well as their metabolites, the amounts of LCBs and their metabolites in chyle from rats administered structurally different pure LCBs were analyzed.

## Methods

### General reagents

*N*-heptadecanoyl-d-erythro-sphingosine [C17 ceramide; Cer 18:1(4E);2OH/17:0], *N*-heptadecanoyl-d-erythro-sphingosylphosphorylcholine [C17 SM; SM 18:1(4E);2OH/17:0], d-glucosyl-1,1′-N-lauroyl-d-erythro-sphingosine [C12 GlcCer; GlcCer 18:1(4E);2OH/12:0], and 2-amino-4-trans-heptadecene-1,3-diol [LCB 17:1(4E);2OH] were obtained from Avanti Polar Lipids (Alabaster, Alabama, USA). Triolein and sodium taurocholate were purchased from Wako Pure Chemical Industries, Ltd. (Osaka, Japan). Caco-2 cells (ECA86010202) were obtained from the RIKEN BioResource Center (Tsukuba, Ibaraki, Japan). Fetal bovine serum (FBS) was purchased from Life Technologies, Inc. (Gaithersburg, MD, USA). Dulbecco’s Modified Eagle Medium (DMEM), non-essential amino acid solution (NEAA), penicillin-streptomycin solution (P/S), trypsin- ethylenediaminetetraacetic acid solution, and β-glucosidase from almonds were obtained from Merck KGaA (Darmstadt, Germany). Six-well Falcon cell culture insert plates (high pore density, pore size 0.4 μm) and 10-cm cell culture dishes were purchased from Corning (New York, USA).

### Extraction and isolation of LCBs

GlcCers with LCB 18:2(4E,8Z);2OH and LCB 18:2(4E,8E);2OH moieties were extracted from konjac tubers (*Amorphophallus konjac*). GlcCers with an LCB 18(9Me):2(4E,8E);2OH moiety were obtained from Tamogi mushroom (*Pleurotus cornucopiae var. citrinopileatus*). CAEPs with LCB 18:3(4E,8E,10E);2OH and LCB 18(9Me):3(4E,8E,10E);2OH moieties were extracted from the giant scallop (*Mizuhopecten yessoensis*). Each sphingolipid was fractionated by silica gel column chromatography as described previously [6, 20]. Purified GlcCers and CAEPs were hydrolyzed in 10% Ba(OH)_2_/dioxane (1:1, v/v) at 110 °C for 24 h [25]. In the case of GlcCers hydrolysis, the resulting materials were lyso-GlcCers, which are deacylated products of GlcCers. To liberate the LCBs, lyso-GlcCers were extracted from the Ba(OH)_2_/dioxane solution using ethyl acetate and dried under reduced pressure. Dried materials were resuspended in acetate buffer (pH 5.0) and treated with β-glucosidase [26]. Liberated LCBs were fractionated on a silica gel, and purified using an Inertsil ODS-3 column (diameter, 20 mm; length, 250 mm; particle size, 5 μm; GL Science, Tokyo, Japan) attached to a high-performance liquid chromatography (HPLC) instrument, resulting in LCB 18:2(4E,8Z);2OH, LCB 18:2(4E,8E);2OH, LCB 18(9Me):2(4E,8Z);2OH, LCB 18:3(4E,8E,10E);2OH, and LCB 18(9Me):3(4E,8E,10E);2OH. LCB purity was measured using HPLC after derivatization with ortho-phthalaldehyde (OPA) [27]. In brief, methanol solutions of LCBs (50 μL) were mixed with an OPA solution (250 μL) and incubated for 15 min at 20 °C. The OPA solution consisted of OPA (5 mg), 2-mercaptoethanol (5 μL), ethanol (100 μL), and 100 mM boric acid-KOH buffer (pH 10.5, 10 mL). The generated OPA-derivatized LCBs were analyzed by HPLC with an ODS-3 column (diameter, 4.6 mm; length, 75 mm; particle size, 3 μm; GL Science). A mixture of acetonitrile and water was used as the eluent, and OPA-derivatized LCBs were eluted at 0.5 mL/min over 50 min across the following gradient: 0–5 min, 55% acetonitrile; 5–15 min, 55–80% acetonitrile; 15–40 min, 80–100% acetonitrile; 40–42 min, 100% acetonitrile; 42–45 min, 100–55% acetonitrile; 45–50 min, 55% acetonitrile. Eluted OPA-LCBs were detected using a fluorescence detector set at excitation and emission wavelength of 340 and 455 nm, respectively.

### Chyle collection from rats administrated LCBs

Male Wistar/ST rats (Japan SLC, Inc., Shizuoka, Japan) aged 9 weeks were housed in individual stainless-steel cages and fed a semi-purified diet (AIN 93G formula) over a 5 day acclimation period. After overnight fasting, a vinyl catheter and a silicone catheter were implanted into the thoracic lymph duct and the duodenum, respectively, as described previously [28]. Test lipids (100 g/L), prepared in 1 mL of emulsified solution, contained 100 mg of triolein and 10 mg of sodium taurocholate (control group) or 90 mg of triolein, 10 mg of each LCB, and 10 mg of sodium taurocholate (LCB-administered groups). Test lipids were emulsified using a sonicator, and emulsions were used for experiments. The molar amounts of LCB administered to rats were as follows: LCB 18:2(4E,8Z);2OH, 33.6 μmol; LCB 18:2(4E,8E);2OH, 33.6 μmol; LCB 18(9Me):2(4E,8Z);2OH, 31.2 μmol; LCB 18:3 (4E,8E,10E);2OH, 33.9 μmol; LCB 18(9Me):3(4E,8E,10E);2OH, 32.3 μmol. After lymph collection for 30 min (initial lymph) on day 1 post-operation, the rats were infused with 1 mL of an emulsified test solution over 1 min via the duodenal tube, and infusion of glucose-NaCl solution through the duodenal tube was continued at 1.8 mL/h until the end of the experiment. Chyle was collected in a test tube, at 1 h intervals for 8 h, following the administration of the test solution. The collected chyle was immediately frozen and stored at − 80 °C until subsequent analyses. Animal experiments were conducted in accordance with the guidelines of the Animal Committee of Iwate University (Authorization No. A201450).

### Lipid extraction

Lipids were extracted from the collected chyle samples, as described previously [6], with slight modifications. The collected chyle samples (0.5 mL) were transferred to glass-capped test tubes and mixed with chloroform (1 mL), methanol (2 mL), phosphate-buffered saline (0.3 mL), and 1 nmol of internal standards [Cer 18:1(4E);2OH/17:0, GlcCer 18:1(4E);2OH/12:0, LCB 17:1(4E);2OH, and SM 18:1(4E);2OH/17:0]. After incubation at 37 °C for 2 h, chloroform (1 mL) and water (1 mL) were added, the mixtures were centrifuged at 1000 *× g* for 5 min, and the lower organic phases were collected into fresh test tubes. Chloroform (1 mL) was added to the upper phase and residual lipids were re-extracted. After centrifugation, the resulting lower organic phases were combined and dried with nitrogen gas. 0.4 M NaOH-methanol solution (1 mL) was added to the extracted lipids and incubated at 37 °C for 2 h to saponify glycerolipids. Chloroform (1 mL) and water (1 mL) were added, and lipids were collected from the lower organic phase and evaporated with nitrogen gas. Dried residues were treated with cold acetone (1 mL) and centrifuged at 1000×*g* at 4 °C for 5 min. The supernatants were discarded, and the pellet was analyzed via liquid chromatography tandem mass spectrometry (LC-MS/MS).

### Culture of Caco-2 cells and LCB treatment

Caco-2 cells were cultured in DMEM supplemented with 10% FBS, NEAA, and P/S (100 U/mL penicillin and 0.1 mg/mL streptomycin) at 37 °C in a humidified 5% CO_2_ incubator. Caco-2 cells were seeded into six-well cell culture inserts at a density of 1.5 × 10^5^ cells/insert. To generate differentiated Caco-2 cells for use as a small intestinal epithelial cell model, Caco-2 cells were cultured for 21 days after reaching confluency. The medium was refreshed every second day. For LCB treatment, 10 mM dimethyl sulfoxide (DMSO) solutions of LCB 18:2(4E,8Z);2OH or LCB 18:2(4E,8E);2OH were mixed with serum-free DMEM at a final concentration of 10 μM. These LCB-serum-free DMEM solutions were added to the cell culture inserts and incubated for 24 h. The final DMSO concentration was adjusted to 0.1%. Basal-side medium and cells were collected, and lipids were extracted as described above. Protein amounts of recovered Caco-2 cells were measured using the BCA protein assay kit (Nacalai Tesque, Kyoto, Japan) according to the manufacture’s protocol and bovine serum albumin was used as standard protein. Lipid amounts were normalized with medium volumes and cellular protein amounts.

### LC-MS/MS analysis of chyle lipids

Chyle were analyzed using a TripleTOF 5600 LC-MS/MS system (AB SCIEX, Foster City, California, USA) equipped with an InertSustain NH2 column (diameter, 2.1 mm; length, 100 mm; particle size, 5 μm; GL Science) in the electrospray ionization (ESI)-positive mode as described previously, with slight modifications [6, 24]. Sphingolipids extracted from chyle were dissolved in 200 μL of mobile phase A, and 10 μL of sample solution was used for sphingolipid analysis. Mobile phase A was acetonitrile:methanol:formic acid:1 M ammonium formate (95:5:0.2:5, v:v:v:v), and mobile phase B was methanol:formic acid:1 M ammonium formate (100:0.2:5, v:v:v). The gradient method was used for analysis as follows: 0–5 min, 0% B; 5–10 min, 0–20% B; 10–12 min, 20% B; 12–15 min, 20–50% B; 15–22 min, 50% B; 22–27 min, 50–80% B; 27–30 min, 80% B; 30–45 min, 80–0% B. The flow rate of the mobile phase was set at 0.13 mL/min. LCB species were identified on the basis of their retention times and high resolution *m/z* of protonated ions; [M + H]^+^ and other sphingolipid species were identified in the parallel reaction monitoring (PRM) mode using characteristic product ions [ceramides and HexCers with an LCB 18:1(4E);2OH moiety for *m/z* 264.2691; ceramides and HexCers with an LCB 18:2(4E,8Z);2OH or LCB 18:2(4E,8E);2OH moiety for *m/z* 262.2535; ceramides and HexCers with an LCB 18:3 (4E,8E,10E);2OH moiety for *m/z* 260.2378; ceramides and HexCers with an LCB 18(9Me):2(4E,8Z);2OH moiety for *m/z* 276.2691; ceramides and HexCers with an LCB 18(9Me):3(4E,8E,10E);2OH moiety for *m/z* 274.2535; SM for *m/z* 184.0864]. MS conditions were as follows: ion spray voltage floating, 5500 V; temperature, 300 °C; declustering potential, 80 V; collision energy, 10 V; ion source gas 1, 20 psi; ion source gas 2, 50 psi; curtain gas, 20 psi; time-of-flight (TOF) accumulation time, 0.2 s; product accumulation time, 0.05 s. To generate product ions, the quadrupole ion isolation width, collision energy, and collision energy spread was set at *m/z* ± 0.7, 35 V, and 5 V, respectively. Data acquisition and analysis was performed using Analyst TF 1.7.1 software and MultiQuant 3.0.1 software (AB SCIEX), respectively. The amounts of target analytes were normalized against recovered lymph volumes and calculated to absolute molar amounts for each collected time.

### SM analysis by in-source collision-induced dissociation (CID)/PRM

To identify the LCB backbones of SMs, the parameters for MS were as follows: negative ion mode for TOF scans; ion spray voltage floating, − 4500 V; temperature, 300 °C; declustering potential, − 300 V; collision energy, − 10 V; ion source gas 1, 20 psi; ion source gas 2, 50 psi; curtain gas, 20 psi; accumulation time, 0.2 s. Detected in-source fragmented [M-CH_3_]^−^ SM ions serving as precursor ions and [M-CH_3_-fatty acyl]^−^ ions providing LCB backbone information were generated by CID. The quadrupole ion isolation width, collision energy, and collision energy spread was set at *m/z* ± 1.5, − 50 V, and 5 V, respectively. The LC conditions were the same as those described above for the positive ion mode. LCB species for SMs were identified from their typical product ions [SMs with an LCB 18:1(4E);2OH moiety for *m/z* 449.31; SMs with an LCB 18:2(4E,8Z);2OH or LCB 18:2(4E,8E);2OH moiety for *m/z* 447.30; SMs with an LCB 18:3 (4E,8E,10E);2OH moiety for *m/z* 445.28; SMs with an LCB 18(9Me):2(4E,8Z);2OH moiety for *m/z* 459.30; SMs with an LCB 18(9Me):3(4E,8E,10E);2OH moiety for *m/z* 457.28].

### Statistical analysis

Statistical analysis was carried out using the GraphPad Prism 9.0.0. To determine statistical significance of the difference among three or more groups (for example, control group, LCB 18:2(4E,8Z);2OH-administrated group, and LCB 18:2(4E,8E);2OH-administrated group), data were analyzed using one-way analysis of variance (ANOVA) followed by Tukey’s multiple comparison test. When comparing only two groups (for example, control group and LCB 18:3 (4E,8E,10E);2OH-administrated group), the parametric Student’s *t*-test was employed. Statistical significance was considered at *P* < 0.05. Data are presented as the mean ± standard deviation.

## Results

### Isolation and purity determination of LCBs

GlcCers and CAEPs were purified from konjac tubers, Tamogi mushrooms, and scallops to prepare LCBs for rat lymph cannulation experiments. The obtained GlcCers and CAEPs were heated in Ba(OH)_2_ aqueous solution/dioxane. Most GlcCers were hydrolyzed to lyso-GlcCers, which were subsequently treated with β-glucosidase and LCBs were liberated. In contrast, CAEPs were completely hydrolyzed to LCB in Ba(OH)_2_ aqueous solution/dioxane. Liberated LCBs were finally purified by ODS HPLC, and LCB 18:2(4E,8Z);2OH, LCB 18:2(4E,8E);2OH, LCB 18(9Me):2(4E,8Z);2OH, LCB 18:3(4E,8E,10E);2OH, and LCB 18(9Me):3(4E,8E,10E);2OH were successfully isolated. Purified LCBs were analyzed by ESI-MS, and their associated ions were observed at *m/z* 298.2719 (calculated for 298.2746, Δ 9.06 ppm) [M + H]^+^ [LCB 18:2(4E,8Z);2OH], 298.2704 (calculated for 298.2746, Δ 14.09 ppm) [M + H]^+^ [LCB 18:2(4E,8E);2OH], 296.2573 (calculated for 296.2573, Δ 5.58 ppm) [M + H]^+^[LCB 18:3(4E,8E,10E);2OH], 312.2872 (calculated for 312.2903, Δ 9.78 ppm) [M + H]^+^ [LCB 18(9Me):2(4E,8Z);2OH], and 310.2727 (calculated for 310.2746, Δ 6.14 ppm) [M + H]^+^ [LCB 18(9Me):3(4E,8E,10E);2OH] (Additional file 1: Fig. S1A-F). Isolated LCBs were derivatized with OPA and analyzed by HPLC with a fluorescence detector. The purities were > 99% for LCB 18:2(4E,8Z);2OH, 95% for LCB 18:2(4E,8E);2OH, > 99% for LCB 18:3(4E,8E,10E);2OH, > 99% for LCB 18(9Me):2(4E,8Z);2OH, and > 99% for LCB 18(9Me):3(4E,8E,10E);2OH (Additional file 1: Fig. S2A-L).

### Recovery of LCBs from chyle

**Fig. 2:**
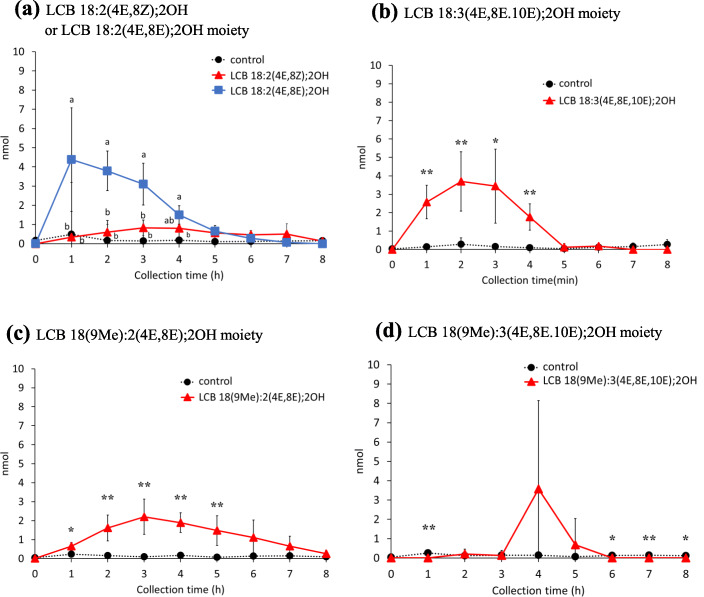
Amounts of long-chain bases in chyle of rats after enteral administration of long-chain base emulsions. **a** LCB 18:2(4E,8Z);2OH or LCB 18:2(4E,8E);2OH, **b** LCB 18:3(4E,8E,10E);2OH, **c** LCB 18(9Me):2(4E,8E);2OH, and **d** LCB 18(9Me):3(4E,8E,10E);2OH. Lipid amounts are normalized with recovered lymph volumes and calculated to absolute amount for each collected time. Values presented as mean ± standard deviation (SD) (*n* = 4–5). Different letters in each time points indicate significant difference at *P* < 0.05. * and ** means significant difference in each time points at *P* < 0.05 and *P* < 0.01. Exact *P* values are listed in Table S2

Emulsions containing 10 mg of LCB (in brief LCB 18:2(4E,8Z);2OH, 33.6 μmol; LCB 18:2(4E,8E);2OH, 33.6 μmol; LCB 18(9Me):2(4E,8Z);2OH, 31.2 μmol; LCB 18:3 (4E,8E,10E);2OH, 33.9 μmol; LCB 18(9Me):3(4E,8E,10E);2OH, 32.3 μmol) emulsified with triolein and taurocholic acid were administered to rats through the duodenum tubes, and chyle were collected from the thoracic duct lymph over time. There were no significant differences in the amount of lymph output among rats administrated with each LCB emulsion (Additional file 1: Fig. S3), indicating that surgery and animal maintenance were carried out appropriately. First, LCBs extracted from the collected chyle and treated with 0.4 M NaOH-methanol solution were analyzed via LC/MS/MS. HPLC retention times and exact masses of protonated ion signals [the [M + H]^+^ *m/z* 298.2746, 298.2746, 296.259, 312.2903, and 310.2746 for LCB 18:2(4E,8Z);2OH, LCB 18:2(4E,8E);2OH, LCB 18:3(4E,8E,10E);2OH, LCB 18(9Me):2(4E,8E);2OH, and LCB 18(9Me):3(4E,8E,10E);2OH, respectively] were used for the identification of each LCB species (Additional file 1: Fig. S4A-E). Peak areas were integrated at *m/z* ± 0.05, and LCB amounts were determined by comparing the [M + H]^+^ ion signals of LCB 18:2(4E,8Z);2OH, LCB 18:2(4E,8E);2OH, LCB 18:3(4E,8E,10E);2OH, LCB 18(9Me):2(4E,8Z);2OH, and LCB 18(9Me):3(4E,8E,10E);2OH with the peak area of the LCB 17:1(4E);2OH internal standard. The amounts of LCBs in chyle at each time point are shown in Fig. 2. The amounts of all LCBs [LCB 18:2(4E,8Z);2OH, LCB 18:2(4E,8E);2OH, LCB 18:3(4E,8E,10E);2OH, LCB 18(9Me):2(4E,8Z);2OH, and LCB 18(9Me):3(4E,8E,10E);2OH], were elevated in the chyle of rats infused with the corresponding LCBs. The amount of LCB 18:2(4E,8Z);2OH was highest at 3 h after administration (0.83 nmol); whereas, the amount of LCB 18:2(4E,8E);2OH, a geometrical isomer of LCB 18:2(4E,8Z);2OH, was highest at 1 h after administration (4.38 nmol). The levels of both LCB 18:2(4E,8Z);2OH and LCB 18:2(4E,8E);2OH at 8 h decreased. The total amount of LCB 18:2(4E,8Z);2OH, up to 8 h after LCB administration, was ~ 4.4-fold higher than that of LCB 18:2(4E,8E);2OH. In the case of other LCBs, the amount of LCB 18:3(4E,8E,10E);2OH in chyle increased after 1–3 h, LCB 18(9Me):2(4E,8Z);2OH increased after 2–4 h, and LCB 18(9Me):3(4E,8E,10E);2OH was detected at 4 h in some samples after administration. This result suggests that the absorption percentages of LCBs in chyle differ with their structure, even among geometrical isomers. Herein, the percentage of absorption of LCB 18:2(4E,8Z);2OH, LCB 18:2(4E,8E);2OH, LCB 18:3(4E,8E,10E);2OH, LCB 18(9Me):2(4E,8Z);2OH, and LCB 18(9Me):3(4E,8E,10E);2OH into chyle as an LCB was 0.011 ± 0.006%, 0.048 ± 0.016%, 0.043 ± 0.016%, 0.004 ± 0.0004%, and 0.014 ± 0.013%, respectively, when integrated up to 8 h after administration (Fig. 2a-d).

### Quantification of ceramide and HexCer with atypical LCB moieties in chyle

**Fig. 3:**
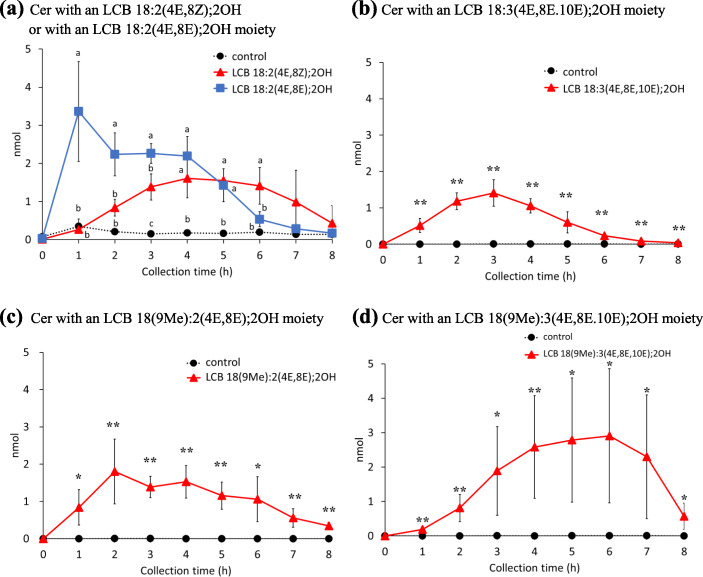
Amounts of ceramides in lymph of rats after enteral administration of long-chain base emulsions. Ceramides with an **a** LCB 18:2(4E,8Z);2OH or LCB 18:2(4E,8E);2OH, **b** LCB 18:3(4E,8E,10E);2OH, **c** LCB 18(9Me):2(4E,8E);2OH, and **d** LCB 18(9Me):3(4E,8E,10E);2OH moiety, in the lymph of rats following enteral administration of long-chain base emulsions. Lipid amounts are normalized with recovered lymph volumes and calculated to absolute amount for each collected time. Values are presented as mean ± standard deviation (SD) (*n* = 4–5). Different letters in each time points indicate significant difference at *P* < 0.05. * and ** means significant difference in each time points at *P* < 0.05 and *P* < 0.01. Exact *P* values are listed in Table S2

As the LCBs absorbed from the digestive tract were expected to be metabolized to ceramides and complex sphingolipids in intestinal epithelial cells, these LCB metabolites in the chyle were also analyzed. As expected, a portion of each administered LCB was processed into ceramides (Fig. 3a-d), and more than 80% were linked to FA 16:0, followed by FA 24:0 and FA 23:0 (Additional file 1: Figs. S5 and 6); moreover, the FA species linked to LCB did not depend on the structure of the administrated LCB (Additional file 1: Fig. S6). Similar to the results of LCB absorption, the amount of ceramide with an LCB 18:2(4E,8E);2OH moiety in chyle peaked at 1 h after administration and then gradually decreased to only trace amounts at 8 h (Fig. 3a). The percentages of ceramides with an LCB 18:2(4E,8Z);2OH moiety or ceramides with an LCB 18:2(4E,8E);2OH moiety absorption into the chyle were compared among groups administrated LCB 18:2(4E,8Z);2OH and LCB 18:2(4E,8E);2OH. As in the case of LCBs, ceramides with an LCB 18:2(4E,8Z);2OH moiety were absorbed more slowly and to a lesser extent than ceramides with an LCB 18:2(4E,8E);2OH moiety (Fig. 3a). In the case of other LCBs, the amount of ceramides with administrated LCBs in the chyle increased at 2–4 h after administration of LCB 18:3(4E,8E,10E);2OH, 2–6 h after administration of LCB 18(9Me):2(4E,8Z);2OH and 3–7 h after administration of LCB 18(9Me):3(4E,8E,10E);2OH (Fig. 3b-d). The absorption of ceramides with an LCB 18(9Me):3(4E,8E,10E);2OH moiety was the slowest among the LCBs tested in this study, and the maximum amount of ceramides detected in chyle recovered 6 h after LCB administration (Fig. 3d). The total absorption percentage of LCB 18:2(4E,8Z);2OH, LCB 18:2(4E,8E);2OH, LCB 18:3(4E,8E,10E);2OH, LCB 18(9Me):2(4E,8Z);2OH, and LCB 18(9Me):3(4E,8E,10E);2OH into chyle as a ceramide was 0.095 ± 0.008%, 0.143 ± 0.023%, 0.030 ± 0.008%, 0.054 ± 0.019%, and 0.088 ± 0.023%, respectively.

**Fig. 4:**
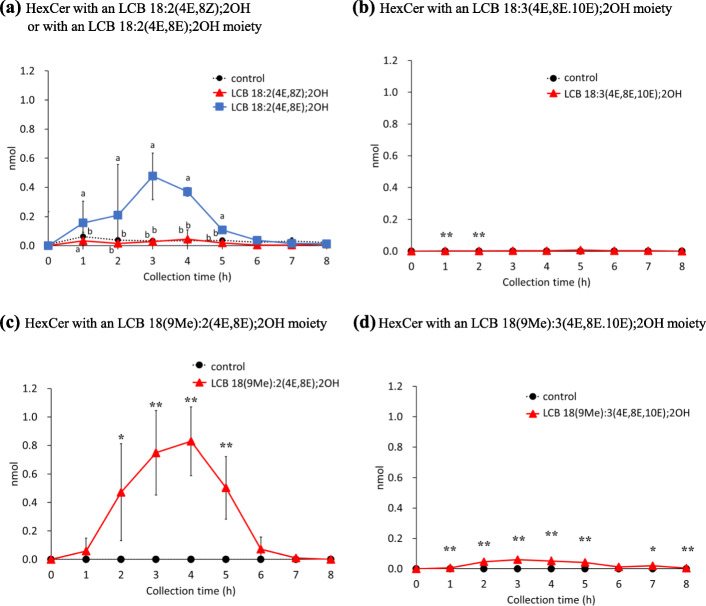
Amounts of hexosylceramides in chyle of rats after enteral administration of long-chain base emulsions. Hexosylceramides with an **a** LCB 18:2(4E,8Z);2OH or LCB 18:2(4E,8E);2OH, **b** LCB 18:3(4E,8E,10E);2OH, **c** LCB 18(9Me):2(4E,8E);2OH, and **d** LCB 18(9Me):3(4E,8E,10E);2OH moiety in the lymph of rats following enteral administration of long-chain base emulsions. Lipid amounts are normalized with recovered lymph volumes and calculated to absolute amount for each collected time. Values are presented as mean ± standard deviation (SD) (*n* = 4–5). Different letters in each time points indicate significant difference at *P* < 0.05. * and ** means significant difference in each time points at *P* < 0.05 and *P* < 0.01. Exact *P* values are listed in Table S2

HexCers metabolized from the administrated LCB in the collected chyle were identified. Interestingly, LCB 18:2(4E,8E);2OH and LCB 18(9Me):2(4E,8Z);2OH were the only LCBs detected in chyle that were converted to HexCers (Fig. 4a, c), which was unlike the observations in case of LCBs or ceramide. HexCers of other LCBs were present only at trace levels (Fig. 4b, d). Only HexCer 18:2(4E,8E);2OH/16:0 and HexCer 18(9Me):2(4E,8E);2OH/16:0 were detected, and no other HexCers linked to FA such as 24:0 and 23:0 were detected in chyle. Changes in the amount of HexCer in the chyle showed a similar trend. The amount of reconstructed HexCer in the chyle was highest at 3 h after administration of LCB 18:2(4E,8E);2OH and at 4 h after administration of LCB 18(9Me):2(4E,8E);2OH (Fig. 4a, c). Both HexCers with LCB 18:2(4E,8E);2OH and LCB 18(9Me):2(4E,8Z);2OH moieties were decreased to trace levels at 6 h after administration (Fig. 4a, c). In addition, the absorption percentages of HexCers as LCB were lower than that of ceramides (Figs. 3a-d and 4a, c). Additionally, it is possible that the transport of HexCer, from the small intestine to chyle, may be slower than that of ceramide. The absorption percentage of HexCers with LCB 18:2(4E,8E);2OH and LCB 18(9Me):2(4E,8Z);2OH into chyle as a HexCer was 0.011 ± 0.004% and 0.017 ± 0.005%, respectively.

### Analysis of the LCB backbones of SM by in-source CID/PRM in ESI-negative mode

To identify and quantify SMs with atypical LCB moieties, lipids extracted from chyle, from rats administered LCBs, were analyzed. In the ESI-positive mode, fragmentation of protonated ions ([M + H]^+^) of SM yielded a major typical product ion (*m/z* 184.0712) derived from phosphocholine; hence, information for LCBs was obtained using this mode (Additional file 1: Fig. S7A). Therefore, the in-source CID/PRM method was established, a combination of in-source CID and post-source CID, in the ESI-negative mode for identification of the LCB backbones of SMs. In this analysis, methyl group liberated by in-source fragmentation of SM ([M-CH_3_]^−^) were observed on the TOF survey scan when the declustering potential was set to − 200 V; hence, [M-CH_3_]^−^ was selected as the precursor ion. The post-source CID of [M-CH_3_]^−^ ions produced the product ion [M-CH_3_-fatty acyl]^−^ (Additional file 1: Fig. S7), and these pairs were selected for the PRM mode (typical TOF-MS spectrums of SM fraction and TOF-MS based XICs which speculated to be demethylated SMs and typical product ion spectrums and XICs of demethylated SMs with atypical LCBs are shown in Additional file 1: Figs. S8 and 9).

**Fig. 5:**
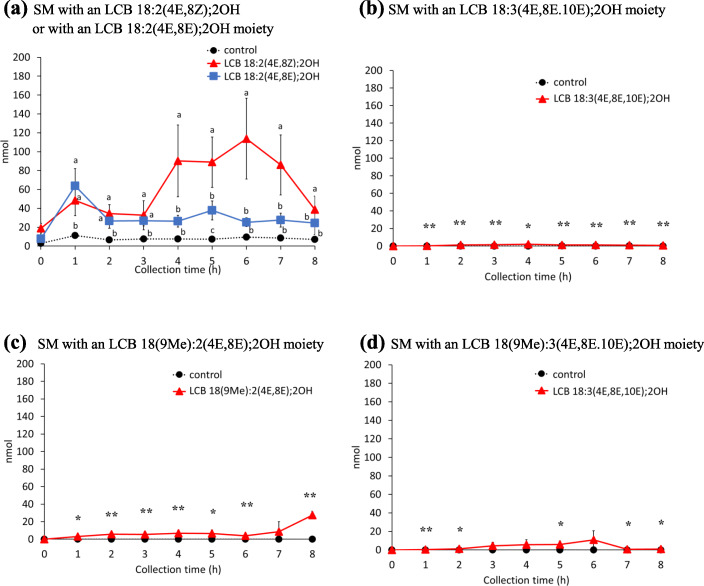
Amounts of sphingomyelins in chyle of rats after enteral administration of long-chain base emulsions. Sphingomyelins with an **a** LCB 18:2(4E,8Z);2OH or LCB 18:2(4E,8E);2OH, **b** LCB 18:3(4E,8E,10E);2OH, **c** LCB 18(9Me):2(4E,8E);2OH, and **d** LCB 18(9Me):3(4E,8E,10E);2OH moiety in the lymph of rats following enteral administration of long-chain base emulsions. Values are presented as mean ± standard deviation (SD) (*n* = 4–5). Lipid amounts are normalized with recovered lymph volumes and calculated to absolute amount for each collected time. Values are presented as mean ± standard deviation (SD) (*n* = 4–5). Different letters in each time points indicate significant difference at *P* < 0.05. * and ** means significant difference in each time points at *P* < 0.05 and *P* < 0.01. Exact *P* values are listed in Table S2

**Fig. 6:**
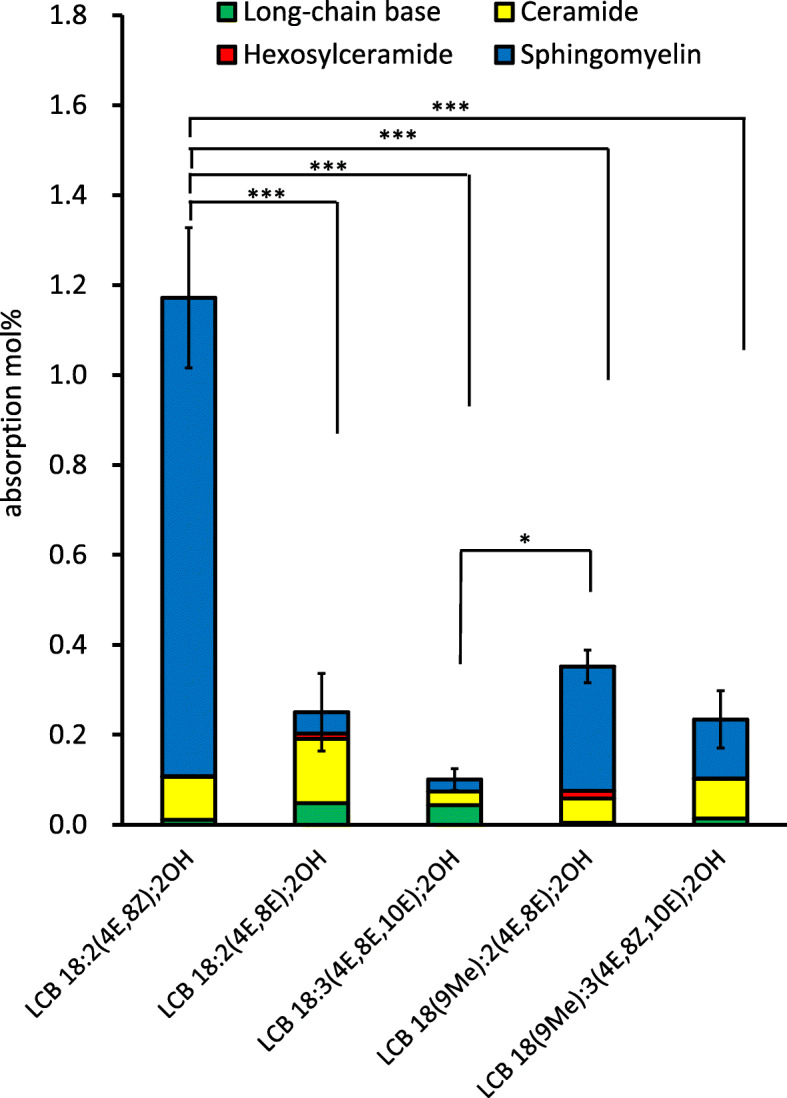
Calculated recovery percentages and sphingolipid class ratios for each long-chain base. Values for the control group were subtracted. * and *** means significant difference in each time points at *P* < 0.05 and *P* < 0.001. Exact *P* values are listed in Table S2

Using this in-source CID/ PRM method in the ESI-negative mode, the levels of SM in the chyle of rats administrated LCBs were measured (Fig. 5). Metabolized SMs from administrated LCBs were detected, especially in the group administered LCB 18:2(4E,8Z);2OH (Fig. 5a). The amount of SMs with an LCB 18:2(4E,8E);2OH moiety in chyle increased at 4–7 h after administration of LCB 18:2(4E,8E);2OH. FA 16:0 was the most common fatty acid in SMs with an LCB 18:2(4E,8E);2OH moiety, as well as ceramides and HexCers with an LCB 18:2(4E,8E);2OH moiety (Figs. 3a, 4a and 5a). Upon comparing the detected SMs with an LCB 18:2(4E,8E);2OH moiety, as in the case of HexCers, the absorption of SM into chyle was observed to occur later than ceramide (Figs. 3a and 5a). In the case of groups administered LCB 18:2(4E,8E);2OH, LCB 18:3(4E,8E,10E);2OH, LCB 18(9Me):2(4E,8Z);2OH, and LCB 18(9Me):3(4E,8E,10E);2OH, the amount of SM detected in chyle was much lower than that in the LCB 18:2(4E,8E);2OH-administered group (Fig. 5b-d). The amount of LCB 18:2(4E,8Z);2OH, LCB 18:2(4E,8E);2OH, LCB 18:3(4E,8E,10E);2OH, LCB 18(9Me):2(4E,8Z);2OH, and LCB 18(9Me):3(4E,8E,10E);2OH adsorbed into chyle as SM was 1.064 ± 0.149%, 0.047 ± 0.068%, 0.026 ± 0.004%, 0.277 ± 0.023%, and 0.131 ± 0.053%, respectively, when integrated up to 8 h after administration. Total absorption percentage of LCB 18:2(4E,8Z);2OH was significantly higher than other LCBs (Fig. 6).

### Uptake and transport of LCB 18:2(4E,8Z);2OH and LCB 18:2(4E,8E);2OH in differentiated Caco-2 cells

**Fig. 7:**
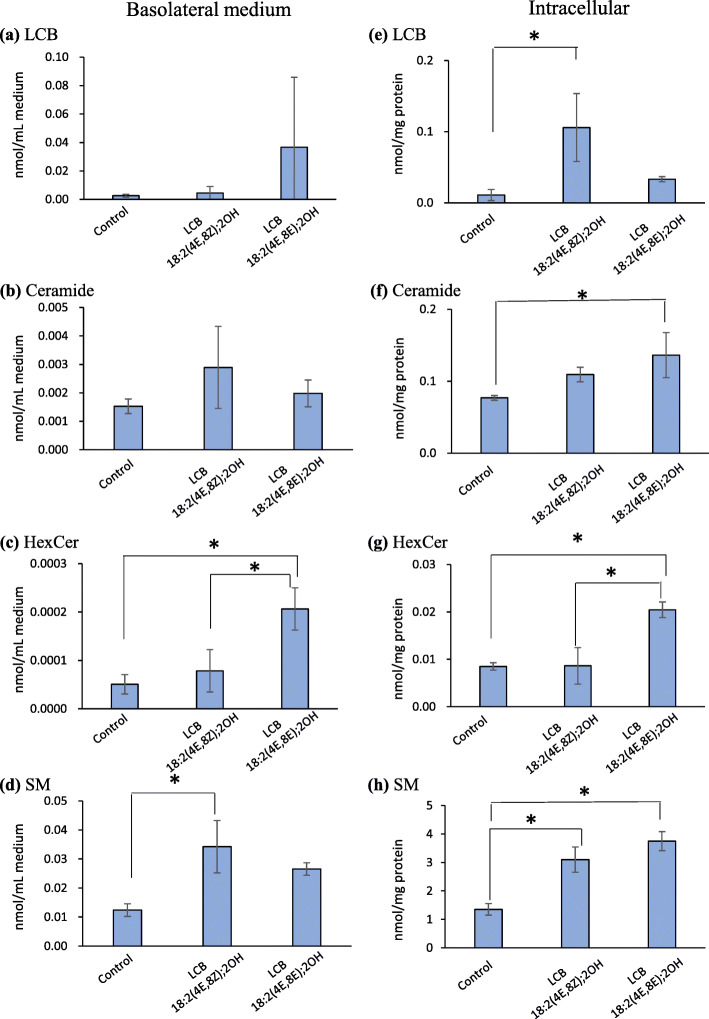
Amount of sphingolipid with an LCB 18:2(4E,8Z);2OH moiety or with an LCB 18:2(4E,8E);2OH moiety. in basolateral medium and Caco-2 cells treated with LCB 18:2(4E,8Z);2OH or LCB 18:2(4E,8E);2OH. **a** Long-chain bases in the basolateral medium. **b** Ceramides in the basolateral medium. **c** Hexosylceramides in the basolateral medium. **d** Sphingomyelins in the basolateral medium. (E) Intracellular long-chain bases. **f** Intracellular ceramides. **g** Intracellular Hexosylceramides. **h** Intracellular sphingomyelins. The Lipid amounts in intracellular and medium are normalized with protein amounts and medium volumes, respectively. Values are presented as mean ± standard deviation (SD) (*n* = 3; *significant difference by Tukey’s multiple comparison test, *P* < 0.05)

In rat chyle, the levels of HexCers and SMs with an LCB 18:2(4E,8Z);2OH or an LCB 18:2(4E,8E);2OH moieties clearly varied with the geometric isomerism of the 8-position of LCB 18:2(4E,8Z);2OH and LCB 18:2(4E,8E);2OH (Figs. 4a and 5a). These LCBs, having the same mass, are considered useful for investigating lipid metabolism and transport. On the other hand, there was no significant difference between LCB 18(9Me):2(4E,8E);2OH and LCB18(9Me):3(4E,8E,10E), thus LCB 18:2(4E,8Z);2OH and LCB 18:2(4E,8E);2OH were used for caco-2 experiments. Thus, the absorption behaviors of LCB 18:2(4E,8Z);2OH and LCB 18:2(4E,8E);2OH were analyzed using differentiated Caco-2 cells, an intestinal epithelial transport system model. Caco-2 cells, which were cultured on a cell culture insert for 21 days and differentiated into intestinal epithelium-like cells, were used for the experiments. The day 22, LCBs dissolved in medium at final concentrations of 10 μM were added to the apical surface of differentiated cells and incubated for 24 h. Lipids were extracted from the medium on the basolateral side and from cells. The LCBs, metabolized to ceramides, HexCers, and SMs were analyzed via LC/MS/MS (Fig. 7, Additional file 1: Fig. S10). There were no significant differences in the amounts of LCBs and ceramides in the medium on the basolateral side, between treatments with LCB 18:2(4E,8Z);2OH or LCB 18:2(4E,8E);2OH (Fig. 7a, b). Levels of HexCers in the basal-side medium were higher in LCB 18:2(4E,8E);2OH-treated cells than LCB 18:2(4E,8Z);2OH-treated cells, and the amounts of SMs in LCB 18:2(4E,8Z);2OH-treated cells were larger than those in LCB 18:2(4E,8E);2OH-treated cells, which was consistent with the rat chyle results. Quantitative analysis revealed that the amount of intracellular LCBs in the LCB 18:2(4E,8Z);2OH-treated cells were significantly higher than those in the control cells, but there was no significant difference between the LCB 18:2(4E,8E);2OH-treated cells and control cells (Fig. 7e). The amount of intracellular ceramides significantly increased only in the LCB 18:2(4E,8E);2OH-treated cells compared with that in the control cells (Fig. 7f), but there was no significant difference between the LCB 18:2(4E,8Z);2OH-treated cells and control cells. The amount of intracellular HexCers significantly increased in the LCB 18:2(4E,8E);2OH-treated cells compared with that in control cells, but there was no change in the amount of HexCers in the LCB 18:2(4E,8Z);2OH-treated cells compared with that in the control cells (Fig. 7g). These results followed a trend similar to that of the experiment on LCB absorption in the chyle of rats. However, unlike the rat experiment, the amount of intracellular SMs of both LCB 18:2(4E,8Z);2OH- and LCB 18:2(4E,8E);2OH-treated cells significantly increased compared with that in the control cells, and there were no significant differences in the amount of intracellular SMs between LCB 18:2(4E,8Z);2OH- and LCB 18:2(4E,8E);2OH-treated cells (Fig. 7h).

## Discussion

To explore the mechanisms underlying the biological effects of dietary sphingolipids, in this study, the absorption and metabolism of atypical LCBs from the digestive tract to the chyle were evaluated in rats. It has been reported that ~ 0.3% of atypical sphingolipids are absorbed into the chyle in rats as LCBs or ceramides, and the amounts of LCBs in chyle are ~ 20 times lower than those of other sphingolipid classes such as ceramide [19, 21]. In addition, the percentage of absorption of LCB 18:1(4E);2OH, a major mammalian LCB, into the chyle is estimated to be 0.73% which calculated to 3 times higher than that of LCB 18:2(4E,8Z);2OH, a plant-derived LCB [19]. In this study, to exclude the efficiency of degradation of sphingolipids in the gastrointestinal tract, the absorption of purified LCBs in rats cannulated for thoracic lymph sampling was examined, and LCBs and sphingolipids metabolized from administrated LCBs in chyle were analyzed via LC/MS/MS. Previous studies have shown that alkyl chain length and the number of double bonds do not have a crucial effect on the ionization efficiency of ESI-MS [29–31]. In addition, ionization intensities of Cer d18:1(4E)/17:0 and synthetic Cers; Cer d18:2(4E,8Z)/16:0 and Cer d18:2(4E,8E)/24:0 were compared (Additional file 1: Fig. S10. Synthesis procedures of Cers with an LCB d18:2 (4E,8Z) moiety were described in Additional file 2. The data of purification and MS analysis for Cers were shown in Additional file 1: Figs. S12-S14). Since there was no significant difference between the each Cer, the effect of LCB and acyl-chain length for ionization efficiency are considered to be little in our analytical methods. In this study, quantification of sphingolipids with atypical LCB moieties were based on the signal intensity of internal standards. The volume of chyle collected per hour were not constant, thus sphingolipid amounts were expressed as absolute molar amounts (Additional file 1: Fig. S3. The data expressed in molar concentrations were shown in Additional file 1: Figs. S15-S17.). The amount of LCB 18:2(4E,8E);2OH in chyle, which has a double bond at C8–C9 in this E form, clearly increased at 1 h after oral administration of LCB 18:2(4E,8E);2OH, and the level was higher than that observed for LCB 18:2(4E,8Z);2OH, which has a double bond at the 8-position in this Z form (Fig. 2a). On the other hand, LCB 18:3(4E,8E,10E);2OH, LCB 18(9Me):2(4E,8Z);2OH, and LCB 18(9Me):3(4E,8E,10E);2OH, which have the same C8–C9 double bond as LCB 18:2(4E,8E);2OH in the E form, appear to be absorbed more slowly in the chyle than LCB 18:2(4E,8E);2OH (Fig. 2). These findings suggest that the absorption rate of LCBs varied, depending on the geometrical isomerism of the C–C double bonds and the presence of the C9 methyl group. The uptake rate of LCBs into epithelial cells may depend on the structure of the LCBs.

Previous studies showed that approximately 0.2–0.3% of GlcCers from maize, whose major LCB is LCB 18:2(4E,8Z);2OH, were absorbed into the chyle of rats [19]. The present analysis showed that the rate of absorption of LCB 18:2(4E,8Z);2OH from the gastrointestinal tract into chyle was ~ 1.2% (Fig. 6). Previous studies have shown that it is also necessary to consider the efficiency of degradation by intestinal sphingolipid digestive enzymes [18, 19]. The absorption percentages calculated in the present work were higher than those in a previous report because purified LCBs were used in the experiments here [19]. The efficiency of sphingolipid digestion is expected to affect absorption, as GlcCers with LCB 18:2(4E,8Z);2OH and LCB 18:2(4E,8Z);2OH exhibited different absorption percentages. Previous studies have reported that triglycerides emulsified to form lipid droplets for oral administration are more easily absorbed than those administered orally as larger lipid droplets or as whole lipids [32, 33]. It is suggested that processing sphingolipids into an easily digestible form may lead to the development of functional foods that can be absorbed efficiently. Up to 1.2% of LCBs were detected in the chyle, but it is unclear where and how the majority of LCBs are present. Some of the LCBs administrated may not be absorbed by the small intestinal epithelium. LCB 18:1(4E);2OH is degraded to FA via the LCB-1-phosphate (LCBP) catalyzed pathway. Since the 1,3-diol-2-amino-4,5-diene moiety is a common structure in LCB 18:1(4E);2OH and atypical LCBs such as LCB 18:2(4E,8Z);2OH, LCB 18:2(4E,8E);2OH, LCB 18:3(4E,8E,10E);2OH, LCB 18(9Me):2(4E,8Z);2OH, and LCB 18(9Me):3(4E,8E,10E);2OH, atypical LCBs may also be degraded to the corresponding FA. The substrate specificities of the enzymes involved in the LCBP lyase-catalyzed pathway may be related to the absorption of LCBs.

Orally administered sphingosine is converted to ceramide and incorporated into chyle [22]. Similar to LCB 18:1(4E);2OH, LCB 18:2(4E,8Z);2OH, LCB 18:2(4E,8E);2OH, LCB 18:3(4E,8E,10E);2OH, LCB 18(9Me):2(4E,8Z);2OH, and LCB 18(9Me):3(4E,8E,10E);2OH are believed to be mainly converted into ceramides after absorption from the gastrointestinal tract and then transferred to the chyle. Ceramides with LCB 18:2(4E,8Z);2OH, LCB 18:2(4E,8E);2OH, LCB 18:3(4E,8E,10E);2OH, LCB 18(9Me):2(4E,8Z);2OH, and LCB 18(9Me):3(4E,8E,10E);2OH moieties were also analyzed by LC/MS/MS. As expected, all the LCBs in this study were detected as ceramides in the rat chyle (Fig. 3). The relationship between absorption percentages and the LCB structure of ceramides displayed the same tendency as that of LCBs. As the absorption percentages of LCBs and ceramides were similar among LCBs, it is speculated that the conversion of incorporated LCBs to ceramides occurs rapidly and that all LCBs are likely to be substrates of ceramide synthase (CerS). In each LCB-administered group, ceramides linked to FA 16:0 were the predominant species in chyle, which are atypical LCBs (Fig. S6). In addition, most HexCer and SM molecular species in chyle were also FA 16:0. These findings suggest that following administration, LCBs were metabolized to ceramides, and these resulting ceramides were used for HexCers and SMs synthesis in intestinal cells. CerS has six isozymes with different substrate specificities; CerS6 has high substrate specificity for coenzyme A linked to the FA 16:0 chain and is the major CerS in mouse intestines [34], and it is a major CerS family member in the rat small intestine.

When SMs were analyzed in ESI-positive mode, a major fragmentation production (*m/z* 184.0712) derived from a phosphocholine moiety was detected (Fig. S7A). SM compounds with the same mass, such as SM 18:1(4E);2OH/24:1 and SM 18:2(4E,8Z);2OH/24:0, were not separated by hydrophilic interaction liquid chromatography. To determine the LCB backbones of SMs, an ion trap mass spectrometer in ESI-negative mode was employed [35]. Alternatively, the ionic products of ceramide generated from SM by in-source fragmentation can be decomposed by post-fragmentation using an atmospheric pressure chemical ionization probe to obtain information regarding LCBs [36]. The addition of alkali metals to the HPLC mobile phase can also yield ions derived from LCBs by CID of SM alkali metal adduct ions (Fig. S7B) [37]. As shown in FigS7A for the analysis of SM 18:1(4E);2OH/17:0, it might have been possible to use identify LCB backbones for SM with an LCB 18:1(4E);2OH moiety; *m/z* = 264.2691 in ESI-positive mode. Similarly, it is expected that it would have been possible to use *m/z* 262.2535 for SMs with an LCB 18:2(4E,8Z);2OH or LCB 18:2(4E,8E);2OH moiety, *m/z* 260.2378 for SMs with an LCB 18:3 (4E,8E,10E);2OH moiety, *m/z* 276.2691 for SMs with an LCB 18(9Me):2(4E,8Z);2OH moiety, *m/z* 274.2535 for SMs with an LCB 18(9Me):3(4E,8E,10E);2OH moiety. To analyze the LCB backbone of SM using a conventional ESI-LC/MS/MS system, a method using a combination of in-source fragmentation and post-source fragmentation in the ESI-negative mode was established. Since [SM-CH_3_]^−^ ions can be observed by ion trap mass spectrometry [35], these ions were selected as precursors. The CID fragment ion of [SM-CH_3_]^−^ was [SM-CH_3_-fatty acid]^−^, which provides information about the LCB structure of SM. This method (in-source CID/PRM) is also useful for quantifying the molecular species of SM involved; hence, the SM species in chyle and cultured cells were analyzed by in-source CID/PRM (Figs. 5 and 7, S10). Comparison of the absorption behavior of LCB metabolites revealed that HexCers and SMs were absorbed slower than ceramides (Figs. 3, 4 and 5). Ceramides are synthesized de novo in the endoplasmic reticulum (ER) and transported to the Golgi by the ceramide transport protein. The transported ceramides are further metabolized to SMs in the Golgi by SM synthase [38]. HexCers synthesis requires the transfer of hexose from uridine diphosphate -hexose to ceramides [39]. Therefore, it has been suggested that differences in the absorption times between HexCer/SM and LCB/ceramide into chyle could be caused by enzymatic reactions. However, it is also possible that differences in absorption times are due to different mechanisms of transport to chyle.

Since the amount of LCBs absorbed depends on their structure, especially the geometric isomerism between LCB 18:2(4E,8Z);2OH and LCB 18:2(4E,8E);2OH, LCB absorption mechanisms were also investigated using Caco-2 cells. On the other hand, in the other LCBs, although there were differences in the lymphatic absorption of HexCer, the effect on the overall absorption percentages were small. In the case of HexCers, LCB 18:2(4E,8E);2OH-treated Caco-2 cells released more HexCers with an LCB 18:2(4E,8E);2OH moiety than the amount of HexCers with an LCB 18:2(4E,8Z);2OH moiety released from LCB 18:2(4E,8Z);2OH-treated Caco-2 cells (Fig. 7c). Compared with the amount of HexCers with an LCB 18:2(4E,8Z);2OH moiety in Caco-2 cells, the amount in LCB 18:2(4E,8E);2OH-treated cells were 1.37-fold higher than that in LCB 18:2(4E,8Z);2OH-treated cells (Fig. 7g). As LCB 18:2(4E,8E);2OH is more easily metabolized to HexCers than LCB 18:2(4E,8Z);2OH, it is presumed that HexCers with an LCB 18:2(4E,8E);2OH moiety accumulated more readily in Caco-2 cells when LCB 18:2(4E,8E);2OH was added than when LCB 18:2(4E,8Z);2OH was added. In the case of LCB 18:2(4E,8E);2OH treatment, which resulted in significantly higher accumulation of HexCer in Caco-2 cells, a larger amount of HexCers was released into the medium compared with LCB 18:2(4E,8Z);2OH treatment. On the other hand, SM levels were significantly higher only when LCB 18:2(4E,8Z);2OH was added, as compared to the control group (Fig. 7d). Although the amount of SMs with an LCB 18:2(4E,8Z);2OH moiety and SMs with an LCB 18:2(4E,8E);2OH moiety in Caco-2 cells was similar for cells treated with both LCB 18:2(4E,8Z);2OH and LCB 18:2(4E,8E);2OH (Fig. 7h), the amount of SMs with an LCB 18:2(4E,8Z);2OH moiety or SMs with an LCB 18:2(4E,8E);2OH moiety released into the medium was greater for the LCB 18:2(4E,8Z);2OH-treated group than for the LCB 18:2(4E,8E);2OH-treated group (Fig. 7d). There was no significant difference in the amounts of SMs with an LCB 18:2(4E,8E);2OH moiety between the LCB 18:2(4E,8E)-treated group and the control group (Fig. 7d). Therefore, it was assumed that SMs with an LCB 18:2(4E,8Z);2OH moiety were more easily transferred to the medium than SMs with an LCB 18:2(4E,8E);2OH moiety. The migration of SMs from cells to the basolateral medium may be related to the structure of the LCB moiety of SMs. The amounts of intracellular HexCers and SMs with an LCB 18:2(4E,8Z);2OH moiety or an LCB 18:2(4E,8E);2OH moiety were increased by LCB 18:2(4E,8Z);2OH or LCB 18:2(4E,8E);2OH treatment, which indicated that the level of SMs was > 100 times greater than that of HexCers (Fig. 7g, h). The transport of HexCers and SMs from Caco-2 cells into the medium was similar to the absorption of HexCers and SMs into chyle (Figs. 4a and 5a). In rat lymphatic cannulation experiments, although LCB 18:2(4E,8E);2OH was more easily metabolized to HexCers than the 8Z form isomer LCB 18:2(4E,8Z);2OH, LCB 18:2(4E,8Z);2OH was predominantly metabolized to SM. Additionally, SMs with an LCB 18:2(4E,8Z);2OH moiety were more easily absorbed into the chyle than SMs with an LCB 18:2(4E,8E);2OH moiety, resulting in a greater amount of LCB 18:2(4E,8Z);2OH being absorbed into the chyle than LCB 18:2(4E,8E);2OH. The double bond at C8–C9 of LCB 18:2(4E,8E);2OH is an E form, which makes this LCB molecule linear, whereas the double bond at C8–C9 of LCB 18:2(4E,8Z);2OH is a Z form, which gives this molecule a bent form. Therefore, it can be concluded that the geometric isomer form of LCBs is a critical factor for their absorption and metabolism. It has been reported that endogenous Cers and SMs exist in chylomicrons [40]. The LCBs used in present study were taken up by small intestinal epithelial cells and metabolized to Cer, SM, and HexCer, which are expected to be incorporated at chylomicrons. There is no direct evidence of this, however, as there are no reports of isolated chylomicrons from mice or rats fed LCB orally. Differences in absorption should also be taken into considerations for differences in distribution to lipid carriers such as chylomicrons. It has been suggested that atypical type LCBs taken up by epithelial cells are exported to the lumenal side by p-glycoprotein [41]. It is necessary to examine the percentage of absorption into the body and the amount of excretion when atypical LCBs are administered to humans in future studies. If the dosage form of LCBs that are easily absorbed can be clarified, and if the amount of absorption and bioactivity differ depending on the type of LCB, it would lead to the development of effective functional foods.

### Study strength and limitation

In the present study, the absorption of purified atypical LCBs into rat chyle was investigated. The total absorption percentage of LCB 18:2(4E,8Z);2OH, which has a Z form double bond at the 8-position, was approximately 1.2%. This percentage was higher than that for any other LCBs, including the geometrical isomer LCB 18:2(4E,8E);2OH, as well as LCBs such as LCB 18:2(4E,8E);2OH, LCB 18(9Me):2(4E,8Z);2OH, LCB 18:3(4E,8E,10E);2OH, and LCB 18(9Me):3(4E,8E,10E);2OH used in this study. This information could be useful for the development of effectively absorbed sphingolipid-containing functional foods. However, it is unclear whether the majority of LCBs that were not absorbed into chyle were excreted or metabolized to other materials. In addition, as all the LCBs used in this study were detected in chyle, any sphingolipids containing LCBs may be useful as a functional food material.

## Conclusions

Atypical LCBs were metabolized to ceramides, HexCers, and SMs in rats and detected in the chyle of rats. Interestingly, the amount of metabolized sphingolipids clearly varied depending on their structure. In particular, geometric isomers at C8–C9 of LCB 18:2(4E,8Z);2OH and LCB 18:2(4E,8E);2OH, which are abundant in higher plants, affect the metabolism and absorption of LCBs. Approximately 90% of the LCB 18:2(4E,8Z);2OH metabolite absorbed into the chyle was SM. Dietary sphingolipids show several beneficial functions, such as skin barrier improvement. The relationship between the absorption of LCBs and their biological activity needs to be clarified in vivo in future studies.

## Supplementary Information

**Additional file 1: Figure S1.** ESI-MS spectra of isolated LCBs from konjac, Tamogi mushroom, and scallop. (A) Blank sample (Solvent). (B) LCB 18:2(4E,8Z);2OH. (C) LCB 18:2(4E,8E);2OH. (D) LCB 18(9Me):2(4E,8E);2OH. (E) LCB 18:3(4E,8E,10E);2OH. (F) LCB 18(9Me):3(4E,8E,10E);2OH. MS spectra were recorded with TripleTOF5600 system. Sample solutions were directly injected TripleTOF5600 system and recorded MS spectrum. The parameters for MS were as follows: positive ion mode for TOF scans; ion spray voltage floating, 5500 V; temperature, 100 °C; declustering potential, 80 V; collision energy, 10 V; ion source gas 1, 15 psi; ion source gas 2, 0 psi; curtain gas, 15 psi; accumulation time, 0.2 s. **Figure S2.** HPLC chromatograms of isolated LCBs from konjac, Tamogi mushroom, and scallop. Signals were monitored by fluorescence detector. (A) Blank sample without OPA. (B) Blank sample with OPA. (C) LCB 18:2(4E,8Z);2OH without OPA. (D) LCB 18:2(4E,8Z);2OH with OPA. (E) LCB 18:2(4E,8E);2OH without OPA. (F) LCB 18:2(4E,8E);2OH with OPA. (G) LCB 18(9Me):2(4E,8E);2OH without OPA. (H) LCB 18(9Me):2(4E,8E);2OH with OPA. (I) LCB 18:3(4E,8E,10E);2OH without OPA. (J) LCB 18:3(4E,8E,10E);2OH with OPA. (K) LCB 18(9Me):3(4E,8E,10E);2OH without OPA. (L) LCB 18(9Me):3(4E,8E,10E);2OH with OPA. **Figure S3.** Cumulative recovery of rat chyle fluid following enteral administration of LCB-containing emulsions. Values are mean ± SD (*n* = 5 for control, *n* = 4 for LCB 18:2(4E,8Z);2OH, *n* = 5 for LCB 18:2(4E,8E);2OH, *n* = 4 for LCB 18:3(4E,8E,10E);2OH, *n* = 4 for LCB 18(9Me):2(4E,8Z);2OH, and *n* = 5 for LCB 18(9Me):3(4E,8E,10E);2OH). **Figure S4.** Extracted ion chromatograms (XIC) of LCBs. Lipids were extracted from chyle of rats administrated atypical LCBs. (A) LCB 18:2(4E,8Z);2OH administrated rat. (B) LCB 18:2(4E,8E);2OH administrated rat. (C) LCB 18:3(4E,8E,10E);2OH administrated rat. (D) LCB 18(9Me):2(4E,8Z);2OH administrated rat. (E) LCB 18(9Me):3(4E,8E,10E);2OH) administrated rat. **Figure S5.** XIC of ceramides with atypical LCBs and hexosylceramides with atypical LCBs. Lipids were extracted from chyle of rats administrated atypical LCBs. (A) LCB 18:2(4E,8Z);2OH administrated rat. (B) LCB 18:2(4E,8E);2OH administrated rat. (C) LCB 18:3(4E,8E,10E);2OH administrated rat. (D) LCB 18(9Me):2(4E,8Z);2OH administrated rat. (E) LCB 18(9Me):3(4E,8E,10E);2OH) administrated rat. **Figure S6.** Changes in the amount of ceramides species. (A) ceramides with an LCB 18:2(4E,8Z);2OH moiety, (B) ceramides with an LCB 18:2(4E,8E);2OH moiety, (C) ceramides with an LCB 18:3(4E,8E,10E);2OH moiety, (D) ceramides with an LCB 18(9Me):2(4E,8Z);2OH moiety and (E) ceramides with an LCB 18(9Me):3(4E,8E,10E);2OH moiety in the chyle of rats following enteral administration of long-chain bases. **Figure S7.** Product ion spectra of SM by ESI-positive mode (A, B) or demethylated SM by ESI-negative mode (C). (A) Product ion spectra of protonated SM 18:1(4E);2OH/17:0 on NaCl-free conditions. (B) Product ion spectra of sodium adducted SM 18:1(4E);2OH/17:0 on NaCl-present conditions. (C) Product ion spectra of demethylated SM 18:1(4E);2OH/17:0. **Figure S8.** TOF survey scan spectra of SMs fraction and XICs which speculated to demethylated SM by insource CID on ESI-negative mode. (A) TOF survey scan spectra of lipids extracted from LCB 18:2(4E,8Z);2OH administered rat chyle. (B) TOF survey scan spectra of lipids extracted from LCB 18:2(4E,8E);2OH administered rat chyle. (C) TOF survey scan spectra of lipids extracted from LCB 18:3(4E,8E,10E);2OH administered rat chyle. (D) TOF survey scan spectra of lipids extracted from LCB 18(9Me):2(4E,8E) administered rat chyle. (E) TOF survey scan spectra of lipids extracted from LCB 18(9Me):3(4E,8E,10E);2OH administered rat chyle. (F) XICs of *m/z* 685.5311, *m/z* 783.6401, *m/z* 797.6565 and *m/z* 701.5612. Since these ions were speculated to be demethylated SM 18:2(4E,8Z);2OH/16:0, demethylated SM 18:2(4E,8Z);2OH/23:0, demethylated SM 18:2(4E,8Z);2OH/24:0 and demethylated SM 18:1(4E);2OH/17:0, respectively, to obtain product ion spectrum CID experiments were carried out. (G) XICs of *m/z* 685.5351, *m/z* 783.6442, *m/z* 797.6594 and *m/z* 701.5638. Since these ions were speculated to be demethylated SM 18:2(4E,8E);2OH/16:0, demethylated SM 18:2(4E,8E);2OH/23:0, demethylated SM 18:2(4E,8E);2OH/24:0 and demethylated SM 18:1(4E);2OH/17:0, respectively, to obtain product ion spectrum CID experiments were carried out. (H) XICs of *m/z* 683.5362, *m/z* 781.6481, *m/z* 795.6627 and *m/z* 701.5814. Since these ions were speculated to be demethylated SM 18:3(4E,8E,10E);2OH/16:0, demethylated SM 18:3(4E,8E10E);2OH/23:0, demethylated SM 18:3(4E,8E10E);2OH/24:0 and demethylated SM 18:1(4E);2OH/17:0, respectively, to obtain product ion spectrum CID experiments were carried out. (I) XICs of *m/z* 699.5501, *m/z* 797.6606, *m/z* 811.6827 and *m/z* 701.5631. Since these ions were speculated to be demethylated SM 18(9Me):2(4E,8E);2OH/16:0, demethylated SM 18(9Me):2(4E,8E);2OH/23:0, demethylated SM 18(9Me):2(4E,8E);2OH/24:0 and demethylated SM 18:1(4E);2OH/17:0, respectively, to obtain product ion spectrum CID experiments were carried out. (J) XICs of *m/z* 697.5340, *m/z* 795.6440, *m/z* 809.6595 and *m/z* 701.5641. Since these ions were speculated to be demethylated SM 18(9Me):3(4E,8E,10E);2OH/16:0, demethylated SM 18(9Me):3(4E,8E10E);2OH/23:0, demethylated SM 18(9Me):3(4E,8E10E);2OH/24:0 and demethylated SM 18:1(4E);2OH/17:0, respectively, to obtain product ion spectrum CID experiments were carried out. **Figure S9.** Typical product ion spectrum of demethylated SMs with an atypical LCB moieties and XICs of SMs based on insource CID/PRM on ESI-negative mode. (A) Product ion spectra of demethylated SM 18:2(4E,8Z);2OH/16:0. (B) Product ion spectra of demethylated SM 18:2(4E,8E);2OH/16:0. (C) Product ion spectra of demethylated SM 18(9Me):2(4E,8E);2OH/16:0. (D) Product ion spectra of demethylated SM 18:3(4E,8E,10E);2OH/16:0. (E) Product ion spectra of demethylated SM 18(9Me):3(4E,8E,10E);2OH/16:0. (F) Product ion spectra of demethylated SM 18:1(4E);2OH/17:0. (L) Product ion spectra of demethylated SM 18:2(4E,8Z);2OH/16:0. (L) Product ion spectra of demethylated SM 18:2(4E,8E);2OH/16:0. (M) Product ion spectra of demethylated SM 18:3(4E,8E,10E);2OH/16:0. (N) Product ion spectra of demethylated SM 18:2(4E,8Z);2OH/16:0. (O) Product ion spectra of demethylated SM 18:2(4E,8E);2OH/16:0. **Figure S10.** Molecular species in the basolateral medium and intracellular sphingolipids with an LCB 18:2(4E,8Z);2OH moiety or LCB 18:2(4E,8E);2OH moiety in Caco-2 cells treated with LCB 18:2(4E,8Z);2OH or LCB 18:2(4E,8E);2OH. for 24 h. (A) Ceramides in the basolateral medium. (B) Hexosylceramides in the basolateral medium. (C) Sphingomyelins in the basolateral medium. (D) Ceramides in cells. (E) Hexosylceramides in cells. (F) Sphingomyelins in cells. Lipid amounts of basolateral media and cells were normalized with medium volumes and cell protein amounts, respectively. Values are presented as mean ± standard deviation (SD) (*n* = 3; *significant difference by Tukey’s multiple comparison test, *P* < 0.05). **Figure S11.** Comparison of the detection intensity of Cer 18:1(4E);2OH/17:0, Cer 18:2(4E,8Z);2OH/16:0 and Cer 18:2(4E,8Z);2OH/24:0. Three Cers were dissolved with acetonitrile/methanol = 19:1 (v/v/) at final concentration of 0.25 μM and 10 μL of Cers solutions were injected onto LC-MS/MS. The experiment was repeated three times. Statistical significances were analyzed by one-way ANOVA folled by Tukey’s multiple comparison test as post hoc test. **Figure S12.** Chromatograms of preparative and analytical HPLC for Cer 18:2(4E,8Z);2OH/16:0. (A) Chromatogram of preparative HPLC. The peak indicated by the asterisks was collected and confirmed to Cer 18:2(4E,8Z);2OH/16:0 by ESI-MS/MS analysis. (B) Analytical HPLC chromatogram of blank sample (solvent). (C) Analytical HPLC chromatogram of purified Cer 18:2(4E,8Z);2OH/16:0. **Figure S13.** Chromatograms of preparative and analytical HPLC for Cer 18:2(4E,8Z);2OH/24:0. (A) Chromatogram of preparative HPLC. The peak indicated by the asterisk was collected and confirmed to Cer 18:2(4E,8Z);2OH/24:0 by ESI-MS/MS analysis. (B) Analytical HPLC chromatogram of blank sample (solvent). (C) Analytical HPLC chromatogram of purified Cer 18:2(4E,8Z);2OH/24:0. **Figure S14.** ESI-MS spectrum of synthetic Cer 18:2(4E,8Z);2OH/16:0 and Cer 18:2(4E,8Z);2OH/24:0. (A) TOF MS spectrum of Cer 18:2(4E,8Z);2OH/16:0. (B) product ion spectrum of Cer 18:2(4E,8Z);2OH/16:0. (C) TOF MS spectrum of Cer 18:2(4E,8Z);2OH/24:0. (D) Product ion spectrum of Cer 18:2(4E,8Z);2OH/24:0. **Figure S15.** Molar concentrations of ceramides in chyle of rats after enteral administration of long-chain base emulsions. (A) Cers with an LCB 18:2(4E,8Z);2OH moiety or an LCB 18:2(4E,8E);2OH moiety, (B) Cers with an LCB 18:3(4E,8E,10E);2OH, (C) Cers with an LCB 18(9Me):2(4E,8E);2OH moiety, and (D) Cers with an LCB 18(9Me):3(4E,8E,10E);2OH moiety in the lymph of rats following enteral administration of long-chain base emulsions. Lipid amounts were normalized with collected chyle volumes. Values are presented as mean ± standard deviation. **Figure S16.** Molar concentrations of hexosylceramides in chyle of rats after enteral administration of long-chain base emulsions. (A) HexCers with an LCB 18:2(4E,8Z);2OH moiety or an LCB 18:2(4E,8E);2OH moiety, (B) HexCers with an LCB 18:3(4E,8E,10E);2OH, (C) HexCers with an LCB 18(9Me):2(4E,8E);2OH moiety, and (D) HexCers with an LCB 18(9Me):3(4E,8E,10E);2OH moiety in the lymph of rats following enteral administration of long-chain base emulsions. Lipid amounts were normalized with collected chyle volumes. Values are presented as mean ± standard deviation (SD) (*n* = 4–5). **Figure S17.** Molar concentrations of shingomyelins in chyle of rats after enteral administration of long-chain base emulsions. (A) SMs with an LCB 18:2(4E,8Z);2OH moiety or an LCB 18:2(4E,8E);2OH moiety, (B) SMs with an LCB 18:3(4E,8E,10E);2OH, (C) SMs with an LCB 18(9Me):2(4E,8E);2OH moiety, and (D) SMs with an LCB 18(9Me):3(4E,8E,10E);2OH moiety in the lymph of rats following enteral administration of long-chain base emulsions. Lipid amounts were normalized with collected chyle volumes. Values are presented as mean ± standard deviation (SD).

**Additional file 2.** Synthesis of Ceramides.

**Additional file 3: Table S1.** Molar amount of all analyzed lipids.

**Additional file 4: Table S2.** Mean and standard deviation values of Figs. 2, 3, 4, 5, 6 and 7 with exact *P* values.

## Data Availability

The dataset supporting the conclusions of this article is available upon request from the corresponding author after publication.

## Abbreviations

LCB: Long-chain base
GlcCer: Glucosylceramide
LC/MS/MS: Liquid chromatography tandem mass spectrometry
FA: Fatty acid
SM: Sphingomyelin
CPE: Ceramide phosphoethanolamine
CAEP: Ceramide 2-aminoethyphosphonate
MS: Mass spectrometry
HexCer: Hexosylceramide
FBS: Fetal bovine serum
NEAA: Non-essential amino acid solution
DMEM: Dulbecco’s Modified Eagle Medium
P/S: Penicillin-streptomycin solution
HPLC: High-performance liquid chromatography
OPA: Ortho-phthalaldehyde
DMSO: Dimethyl sulfoxide
ESI: Electrospray ionization
PRM: Parallel reaction monitoring
TOF: Time-of-flight
CID: Collision-induced dissociation
ANOVA: Analysis of variance
CerS: Ceramide synthase
ER: Endoplasmic reticulum
