# Supplementary material for: Structure-dependent absorption of atypical sphingoid long-chain bases from digestive tract into lymph
Source: Lipids Health Dis. 2021 Mar 1;20:24. doi: 10.1186/s12944-021-01448-2 (PMC7919070; doi:10.1186/s12944-021-01448-2)
Supplement: Supplementary file 1 — Additional file 1: Figure S1. ESI-MS spectra of isolated LCBs from konjac, Tamogi mushroom, and scallop. (A) Blank sample (Solvent). (B) LCB 18:2(4E,8Z);2OH. (C) LCB 18:2(4E,8E);2OH. (D) LCB 18(9Me):2(4E,8E);2OH. (E) LCB 18:3(4E,8E,10E);2OH. (F) LCB 18(9Me):3(4E,8E,10E);2OH. MS spectra were recorded with TripleTOF5600 system. Sample solutions were directly injected TripleTOF5600 system and recorded MS spectrum. The parameters for MS were as follows: positive ion mode for TOF scans; ion spray voltage floating, 5500 V; temperature, 100 °C; declustering potential, 80 V; collision energy, 10 V; ion source gas 1, 15 psi; ion source gas 2, 0 psi; curtain gas, 15 psi; accumulation time, 0.2 s. Figure S2. HPLC chromatograms of isolated LCBs from konjac, Tamogi mushroom, and scallop. Signals were monitored by fluorescence detector. (A) Blank sample without OPA. (B) Blank sample with OPA. (C) LCB 18:2(4E,8Z);2OH without OPA. (D) LCB 18:2(4E,8Z);2OH with OPA. (E) LCB 18:2(4E,8E);2OH without OPA. (F) LCB 18:2(4E,8E);2OH with OPA. (G) LCB 18(9Me):2(4E,8E);2OH without OPA. (H) LCB 18(9Me):2(4E,8E);2OH with OPA. (I) LCB 18:3(4E,8E,10E);2OH without OPA. (J) LCB 18:3(4E,8E,10E);2OH with OPA. (K) LCB 18(9Me):3(4E,8E,10E);2OH without OPA. (L) LCB 18(9Me):3(4E,8E,10E);2OH with OPA. Figure S3. Cumulative recovery of rat chyle fluid following enteral administration of LCB-containing emulsions. Values are mean ± SD (n = 5 for control, n = 4 for LCB 18:2(4E,8Z);2OH, n = 5 for LCB 18:2(4E,8E);2OH, n = 4 for LCB 18:3(4E,8E,10E);2OH, n = 4 for LCB 18(9Me):2(4E,8Z);2OH, and n = 5 for LCB 18(9Me):3(4E,8E,10E);2OH). Figure S4. Extracted ion chromatograms (XIC) of LCBs. Lipids were extracted from chyle of rats administrated atypical LCBs. (A) LCB 18:2(4E,8Z);2OH administrated rat. (B) LCB 18:2(4E,8E);2OH administrated rat. (C) LCB 18:3(4E,8E,10E);2OH administrated rat. (D) LCB 18(9Me):2(4E,8Z);2OH administrated rat. (E) LCB 18(9Me):3(4E,8E,10E);2OH) administrated rat. Figure S5. XIC of ceramides [file 12944_2021_1448_MOESM1_ESM.pdf]

Supplemental information for "Structure-dependent absorption of atypical sphingoid long-chain bases from the digestive tract into lymph"

Additional file 1

Figure S1.

(A) Blank sample (Solvent)

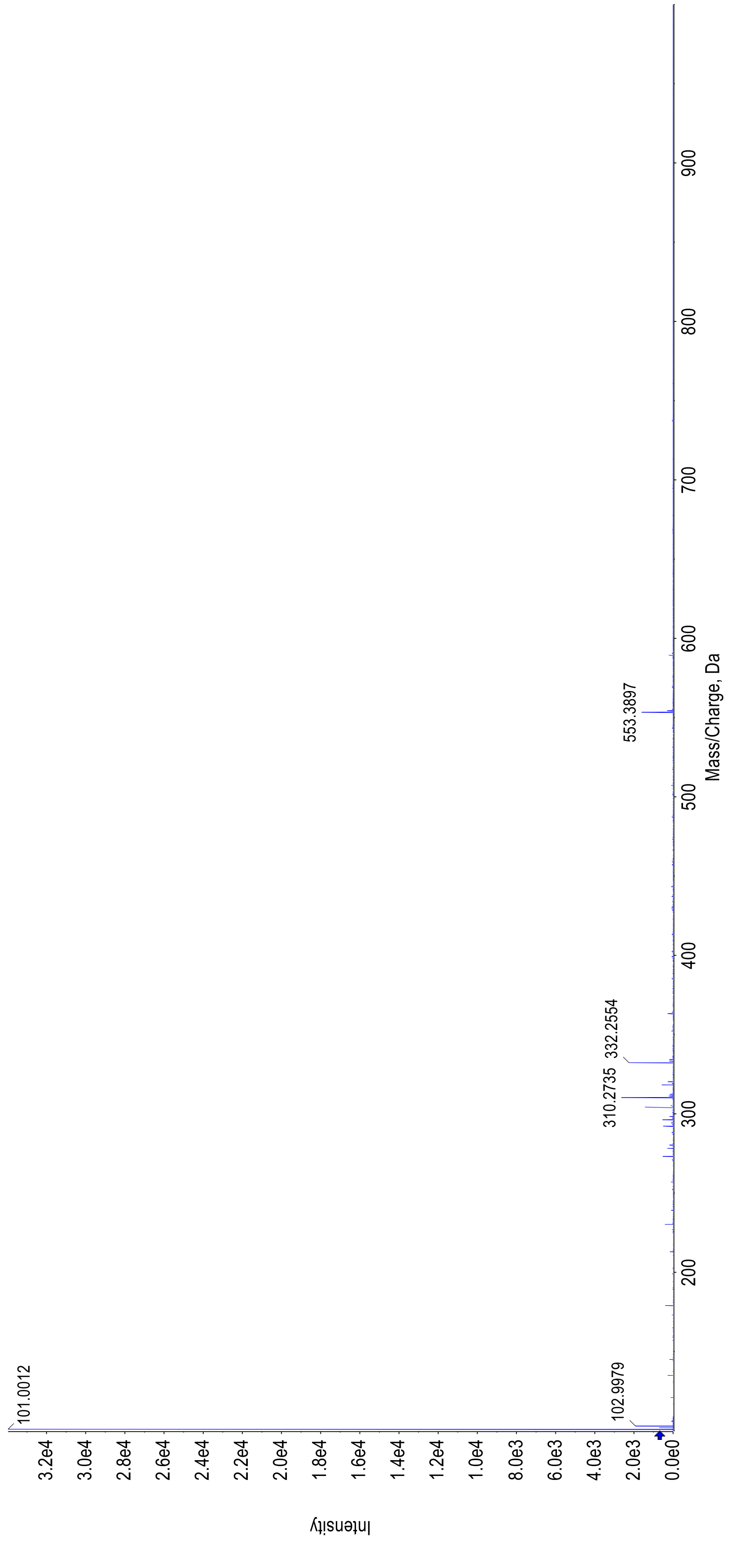

Figure S1. Continue

(B) LCB 1:2(4E,8Z);2OH

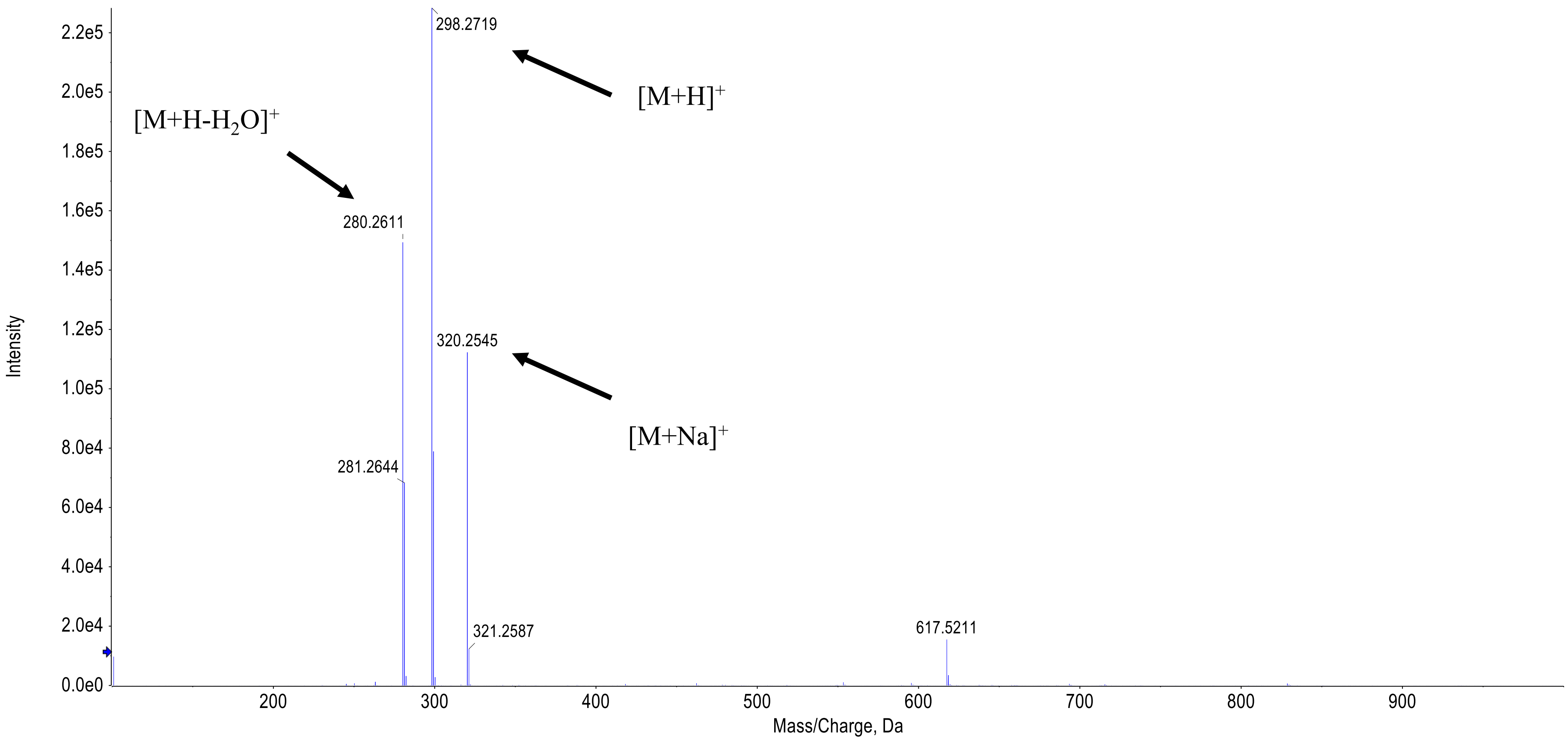

Figure S1. Continue

(C) LCB 18:2(4E,8E);2OH

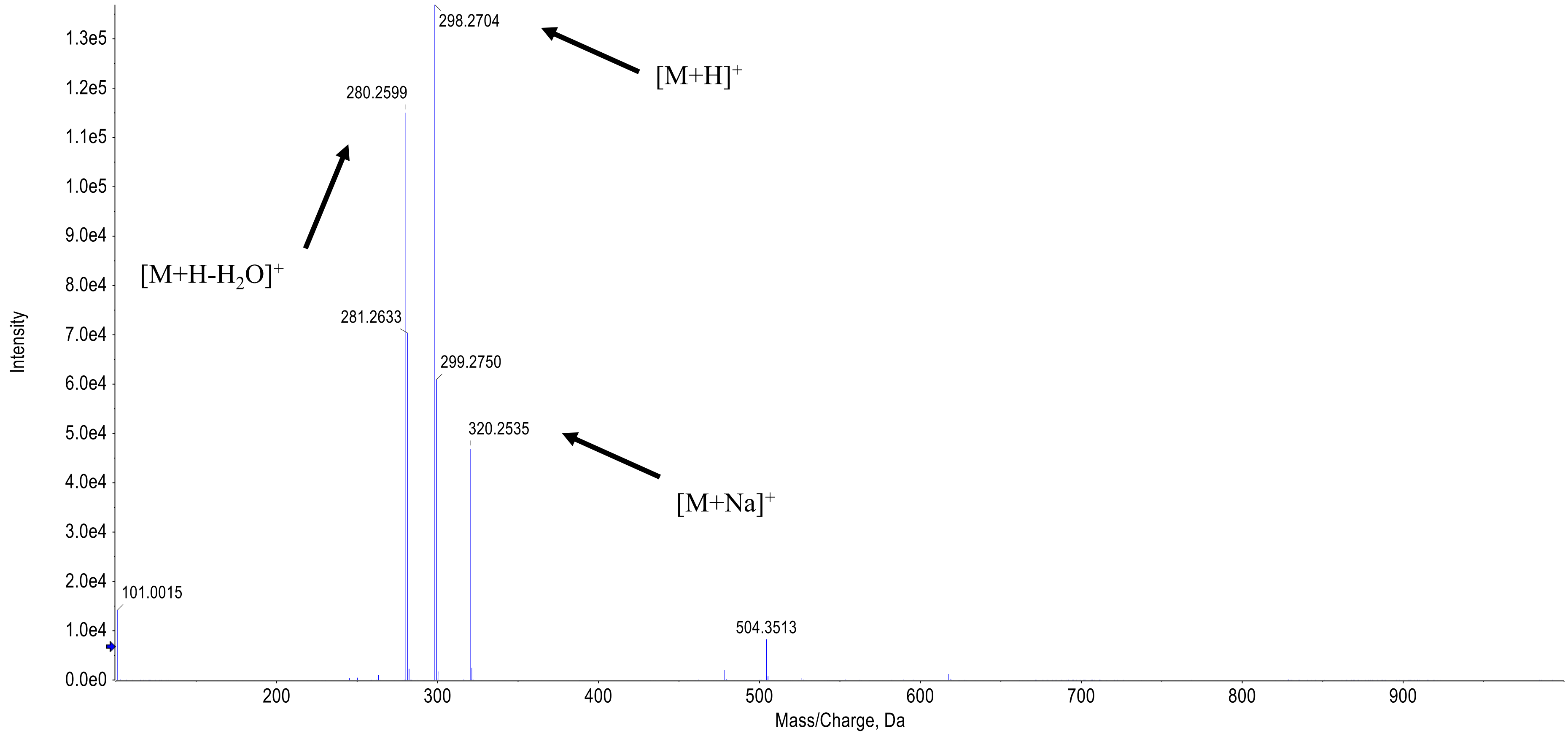

Figure S1. Continue

(D) LCB 18:3(4E,8E,10E)

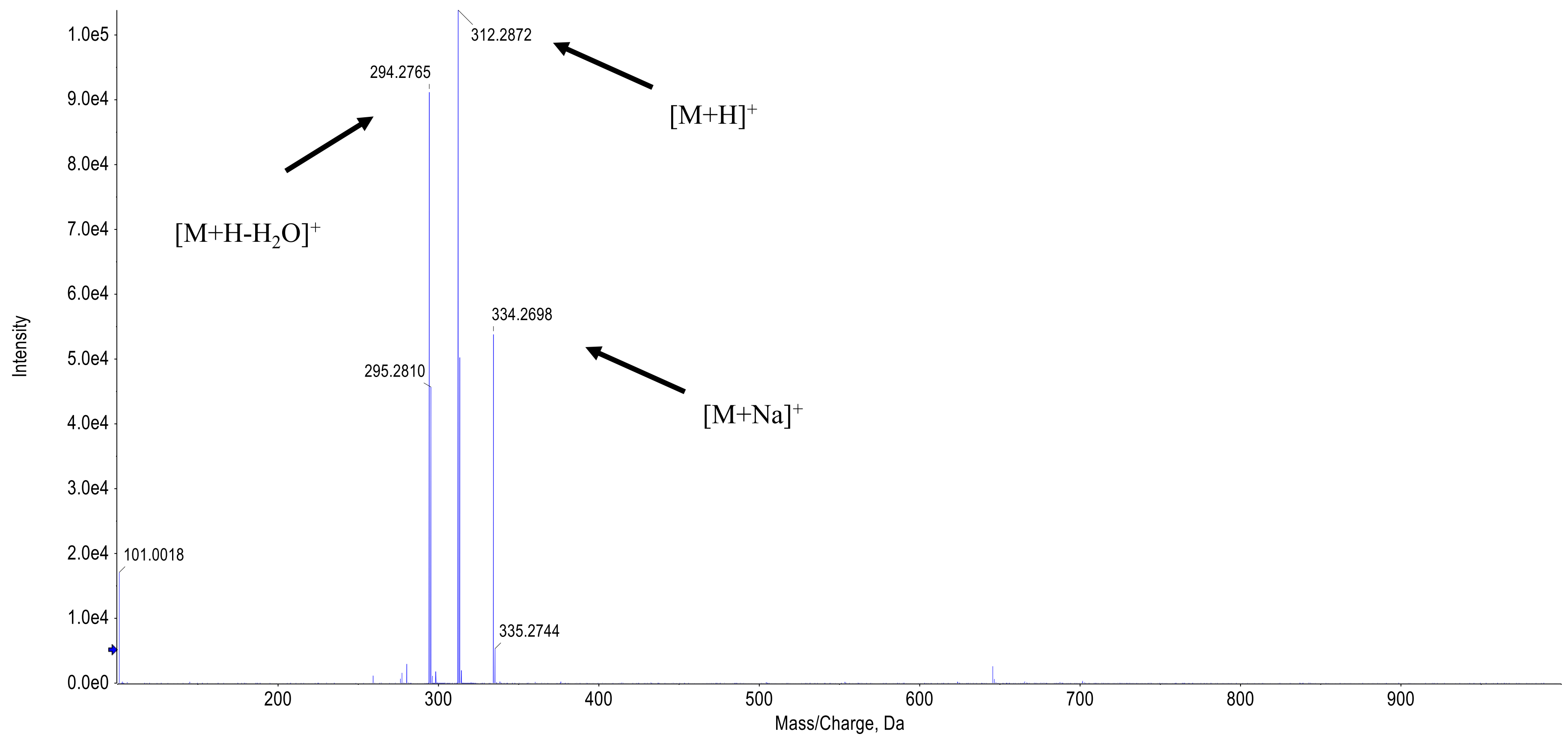

Figure S1. Continue

(E) LCB 18(9Me):2(4E,8E);2OH

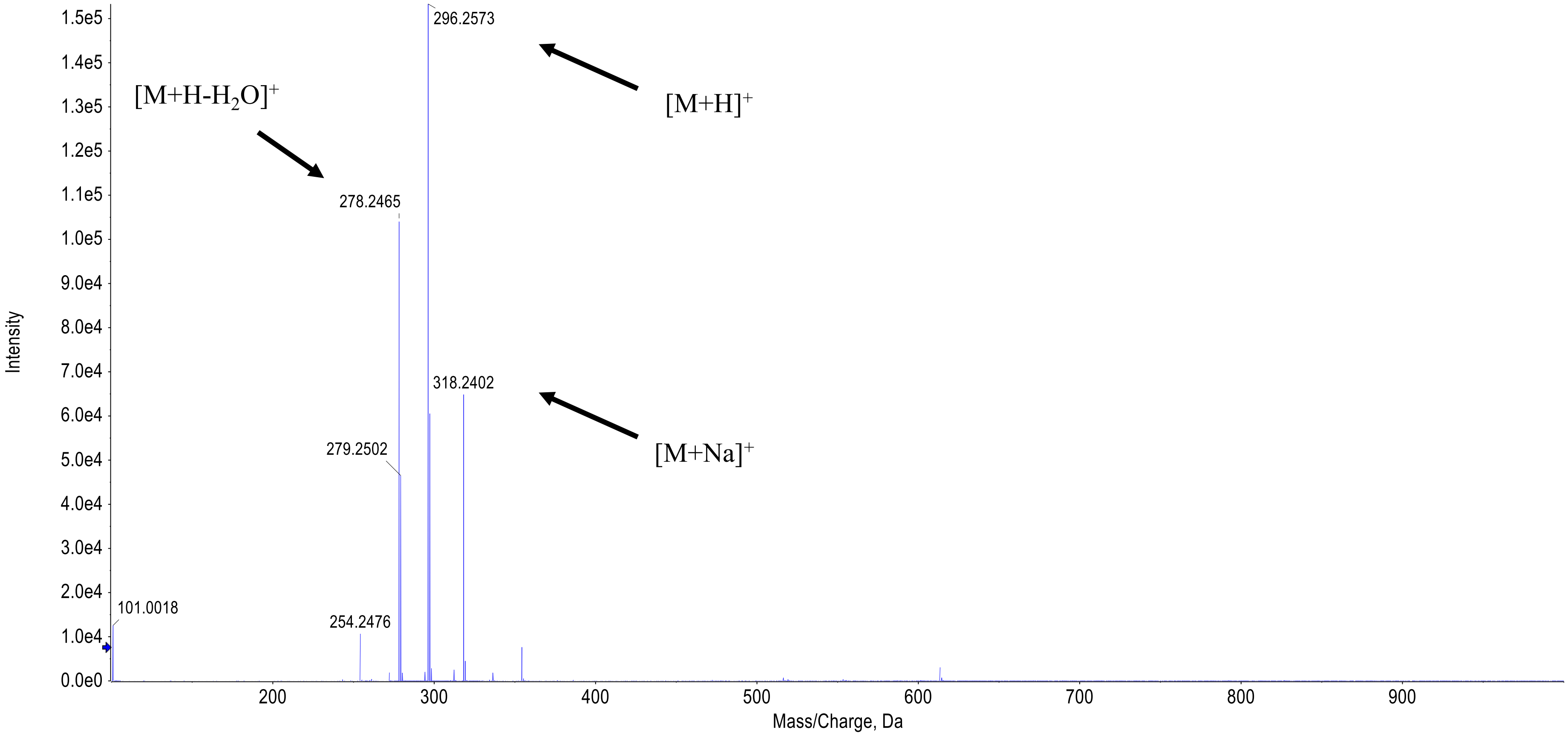

Figure S1. Continue

(F) LCB 18(9Me):2(4E,8E);2OH

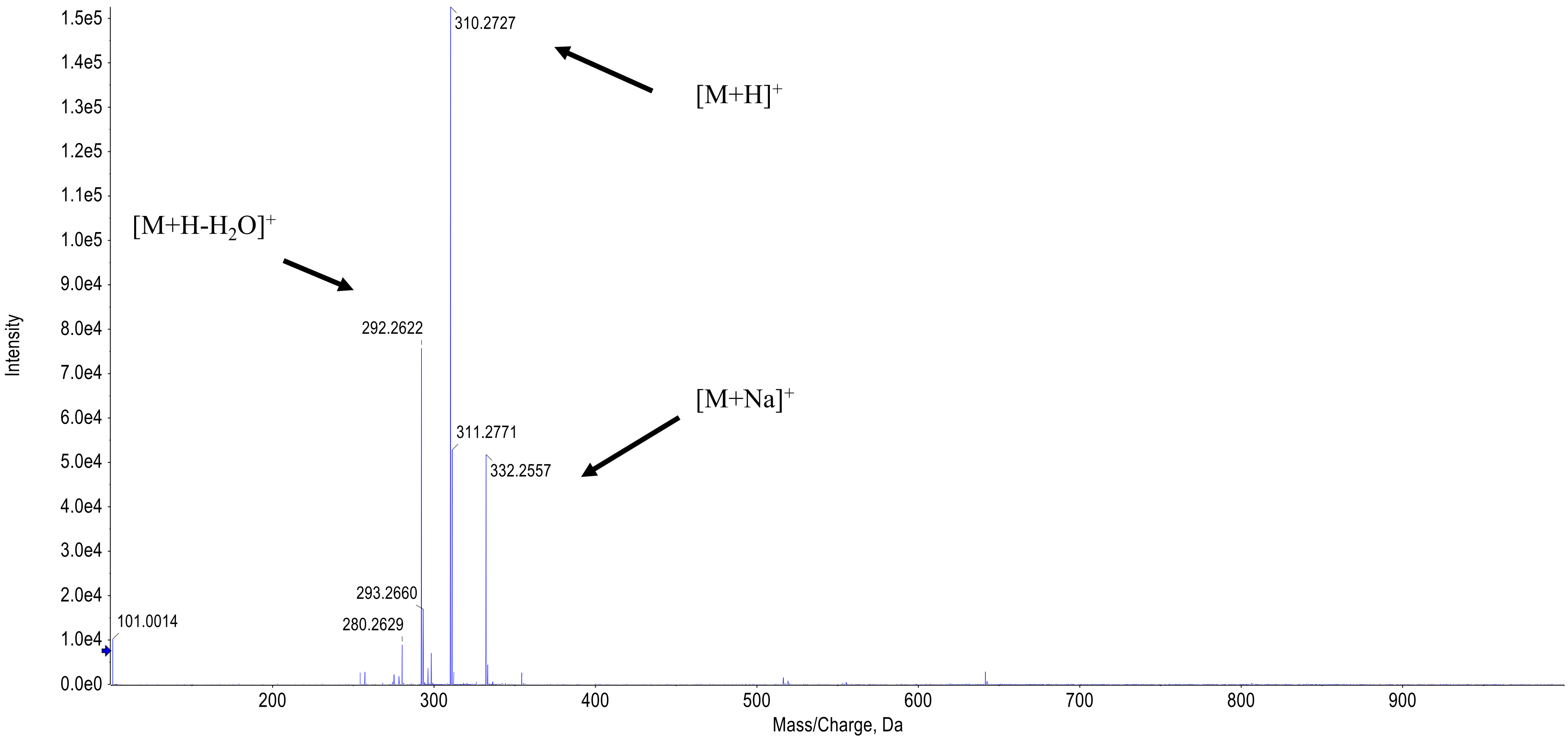

Figure S1: ESI-MS spectra of isolated LCBs from konjac, Tamogi mushroom, and scallop. (A) Blank sample (Solvent). (B) LCB 18:2(4E,8Z);2OH. (C) LCB 18:2(4E,8E);2OH. (D) LCB 18(9Me):2(4E,8E);2OH. (E) LCB 18:3(4E,8E,10E);2OH. (F) LCB 18(9Me):3(4E,8E,10E);2OH. MS spectra were recorded with TripleTOF5600 system. Sample solutions were directly injected TripleTOF5600 system and recorded MS spectrum. The parameters for MS were as follows: positive ion mode for TOF scans; ion spray voltage floating, 5500 V; temperature, 100° C; declustering potential, 80 V; collision energy, 10 V; ion source gas 1, 15 psi; ion source gas 2, 0 psi; curtain gas, 15 psi; accumulation time, 0.2

Figure S2.

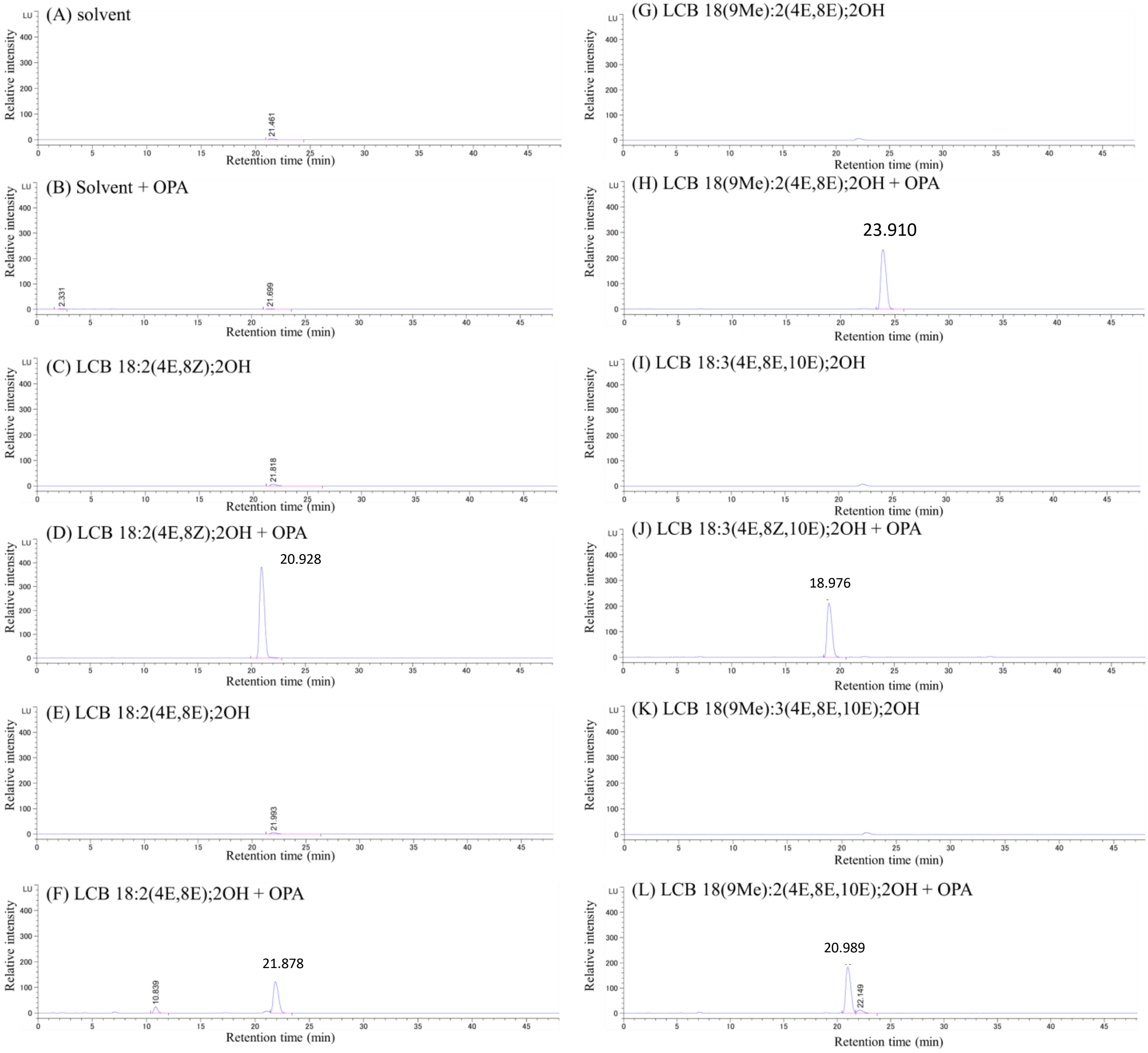

Figure S2: HPLC chromatograms of isolated LCBs from konjac, Tamogi mushroom, and scallop. Signals were monitored by fluorescence detector. (A) Blank sample without OPA. (B) Blank sample with OPA. (C) LCB 18:2(4E,8Z);2OH without OPA. (D) LCB 18:2(4E,8Z);2OH with OPA. (E) LCB 18:2(4E,8E);2OH without OPA. (F) LCB 18:2(4E,8E);2OH with OPA. (G) LCB 18(9Me):2(4E,8E);2OH without OPA. (H) LCB 18(9Me):2(4E,8E);2OH with OPA. (I) LCB 18:3(4E,8E,10E);2OH without OPA. (J) LCB 18:3(4E,8E,10E);2OH with OPA. (K) LCB 18(9Me):3(4E,8E,10E);2OH without OPA. (L) LCB 18(9Me):3(4E,8E,10E);2OH with OPA.

Figure S3.

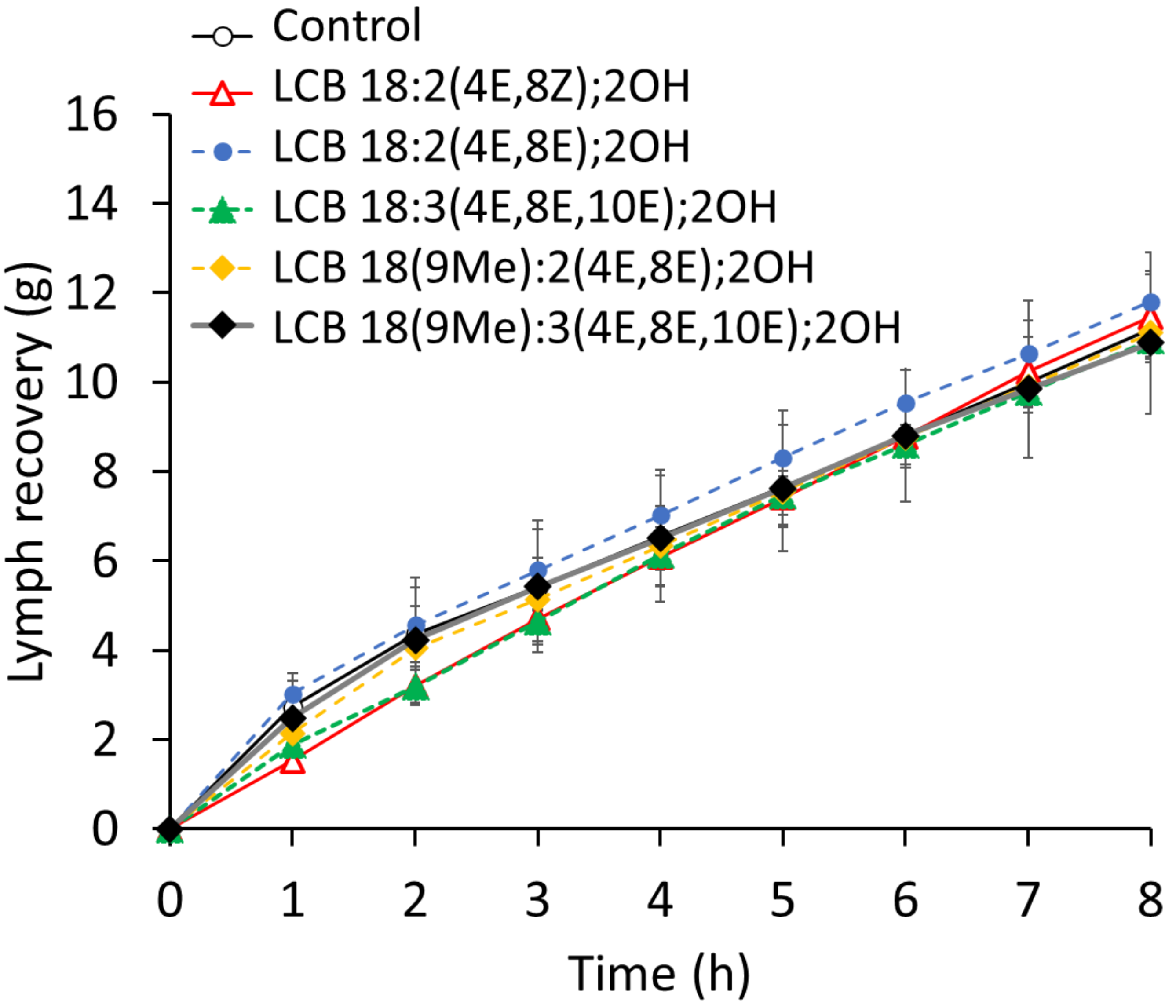

Figure S3: Cumulative recovery of rat chyle fluid following enteral administration of LCB-containing emulsions. Values are mean ± SD (n = 5 for control, n = 4 for LCB 18:2(4E,8Z);2OH, n = 5 for LCB 18:2(4E,8E);2OH, n = 4 for LCB 18:3(4E,8E,10E);2OH, n = 4 for LCB 18(9Me):2(4E,8Z);2OH, and n = 5 for LCB 18(9Me):3(4E,8E,10E);2OH).

Figure S4.

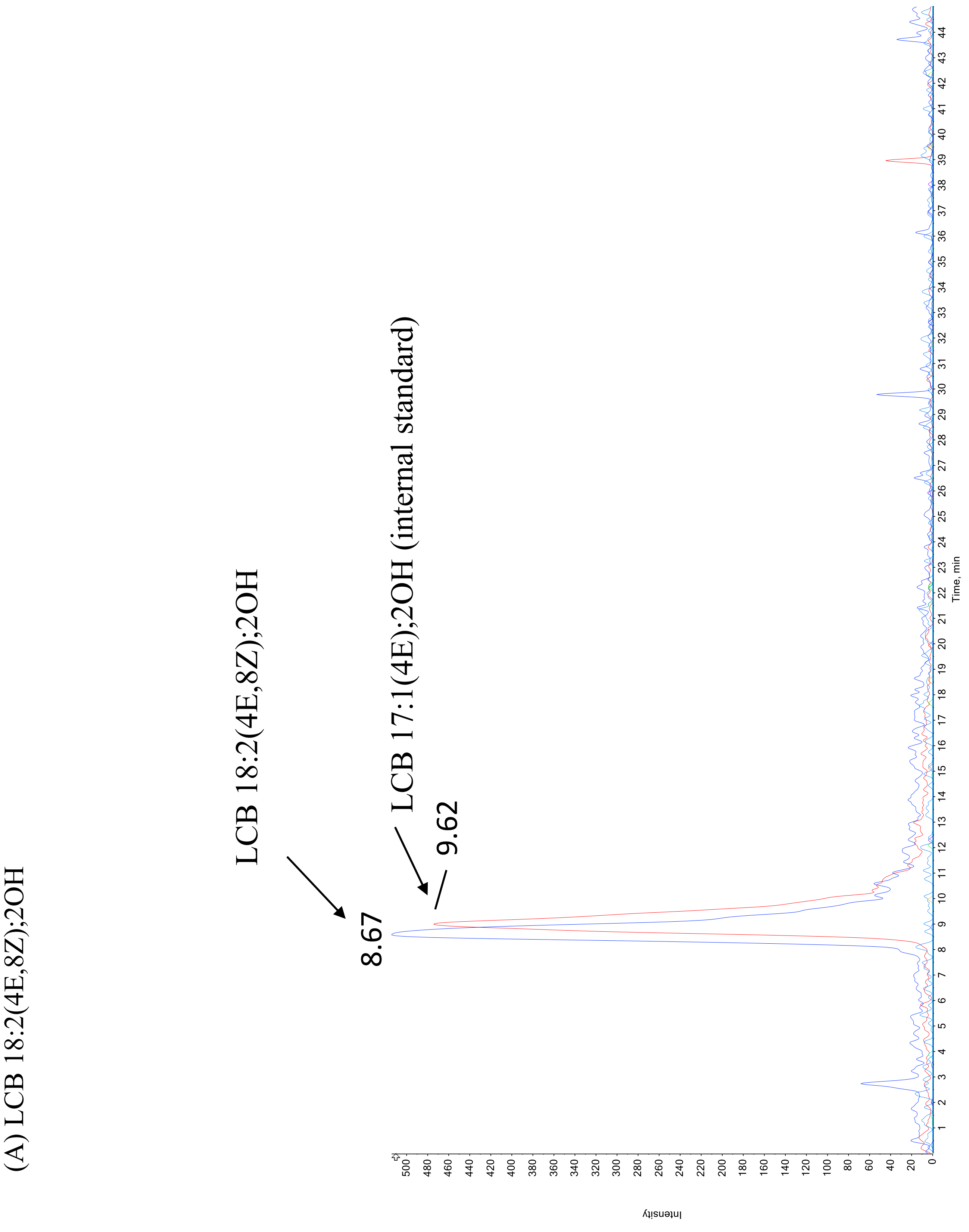

(A) LCB 18:2(4E,8Z);2OH
LCB 18:2(4E,8Z);2OH
8.67
LCB 17:1(4E);2OH (internal standard)
9.62
Intensity
Time, min

Figure S4. Continue.

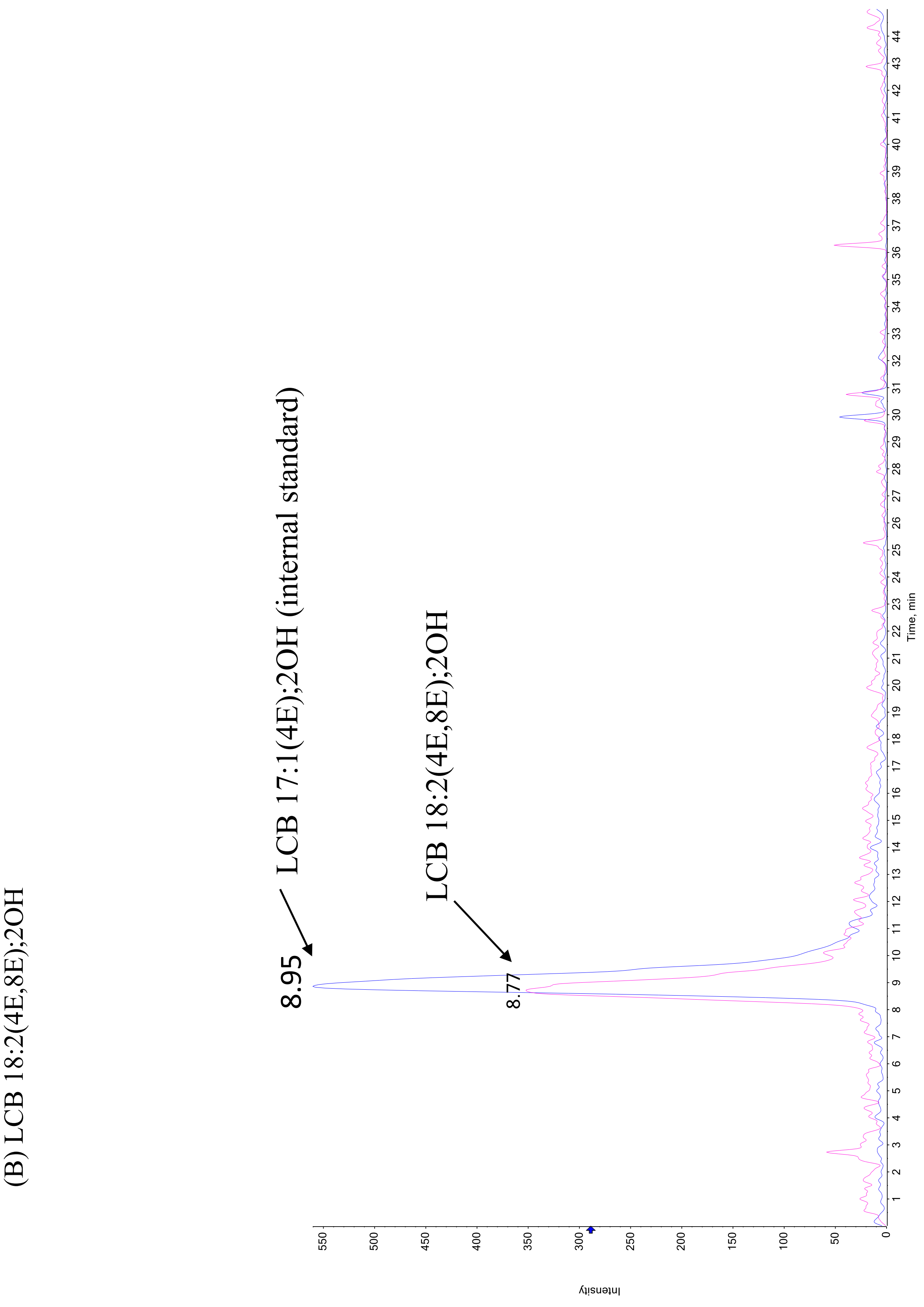

Figure S4. Continue.

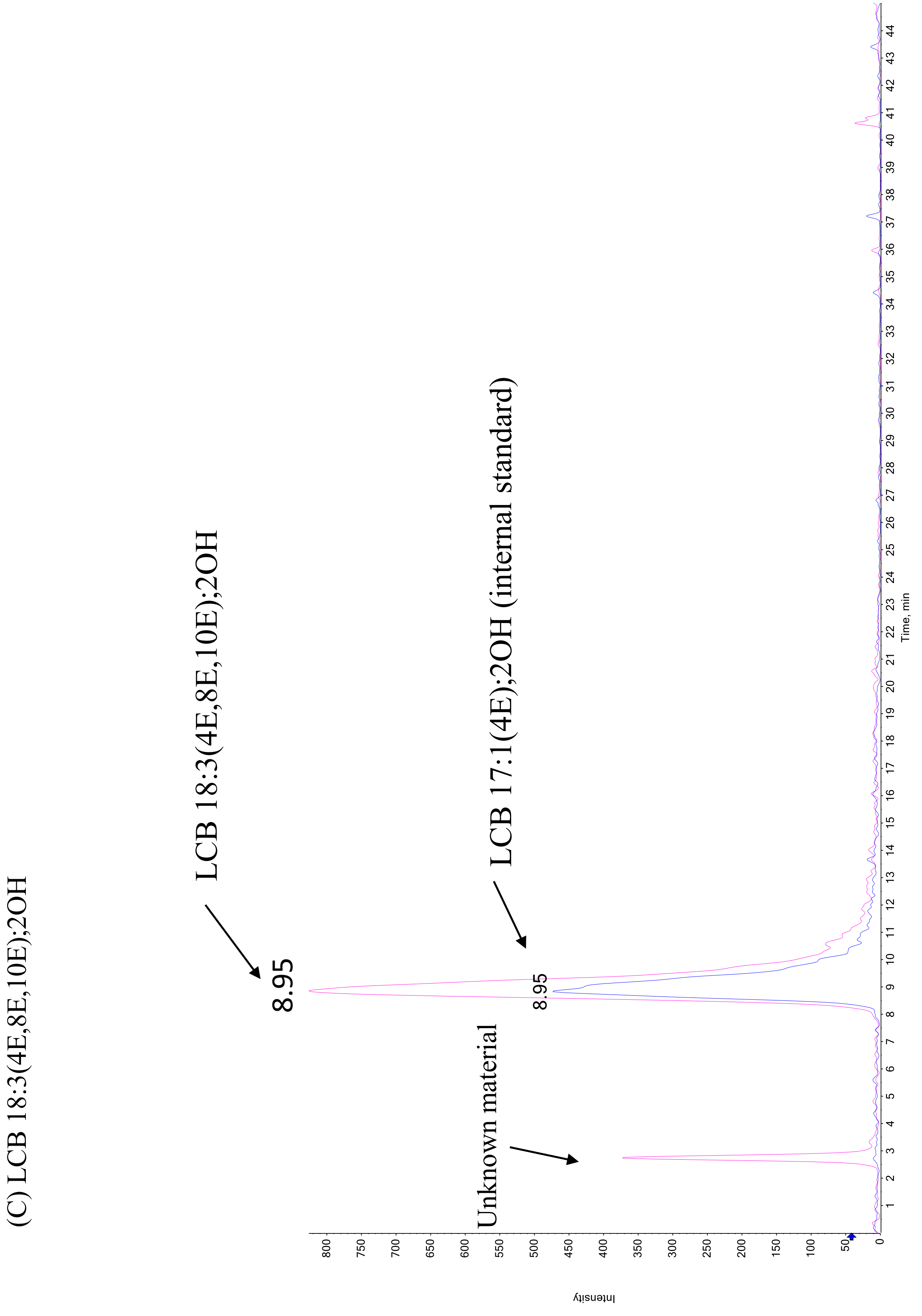

Figure S4. Continue.

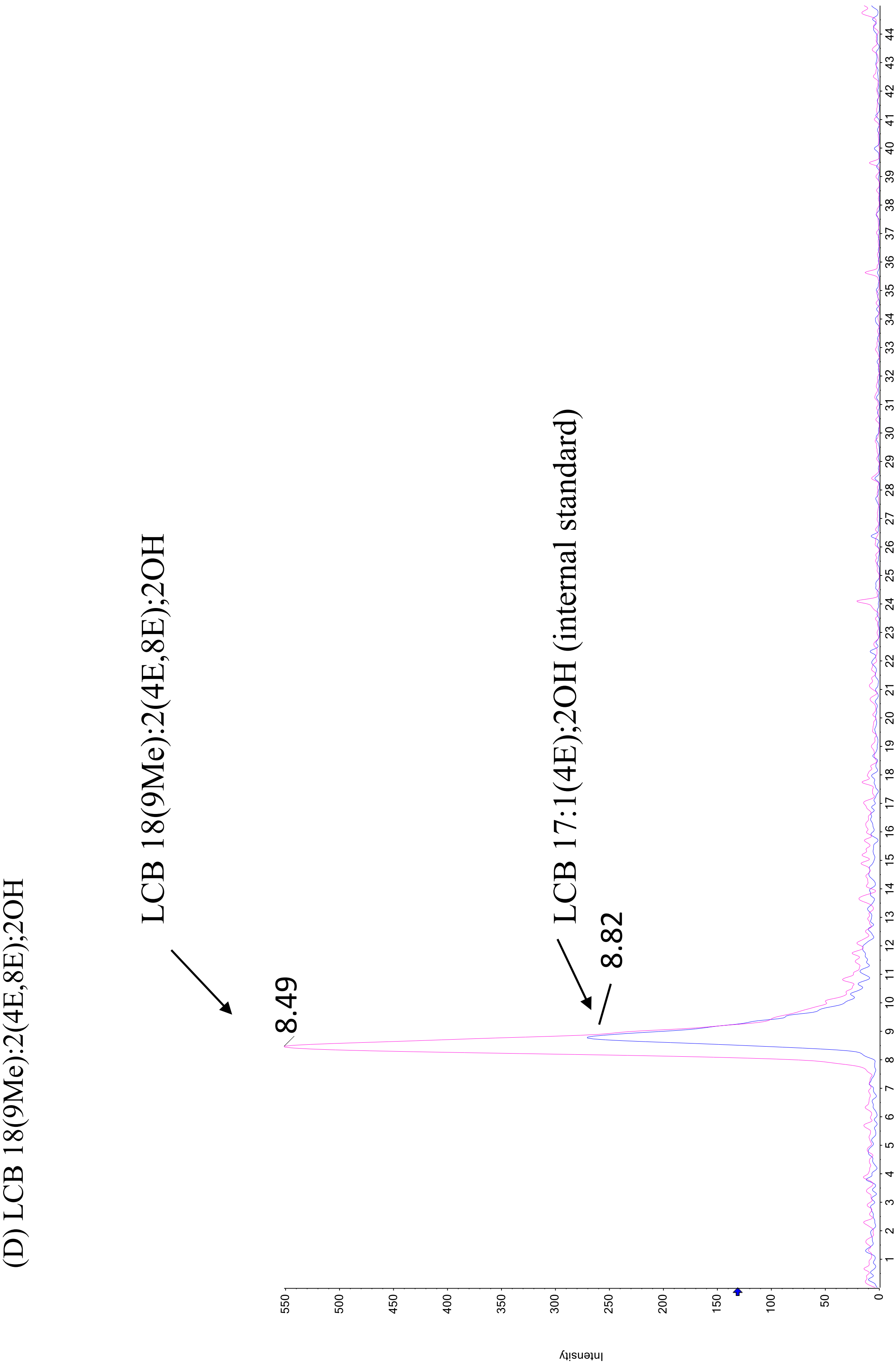

Figure S4. Continue.

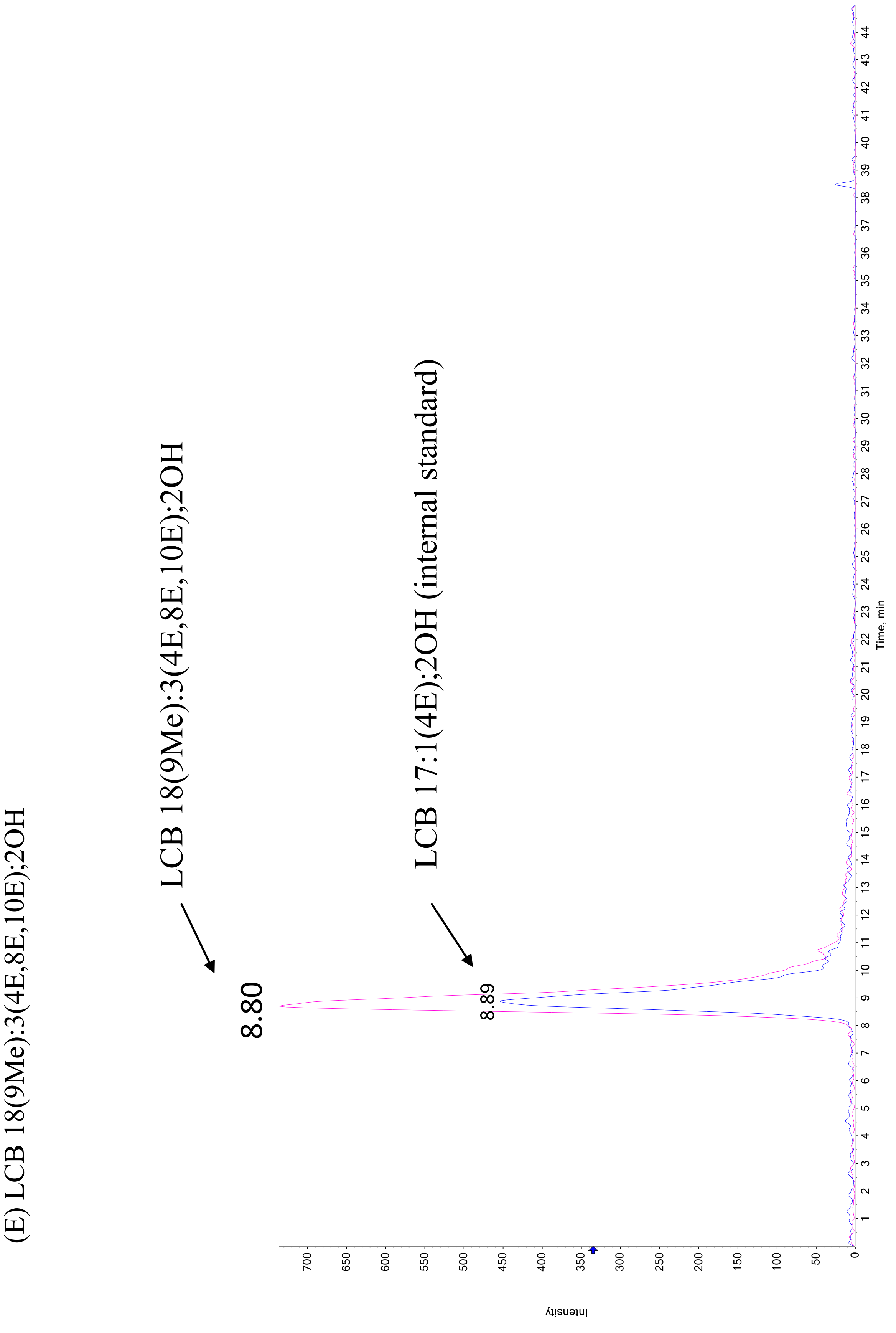

Figure S4: Extracted ion chromatograms (XIC) of LCBs. Lipids were extracted from chyle of rats administrated atypical LCBs. (A) LCB 18:2(4E,8Z);2OH administrated rat. (B) LCB 18:2(4E,8E);2OH administrated rat. (C) LCB 18:3(4E,8E,10E);2OH administrated rat. (D) LCB 18(9Me):2(4E,8Z);2OH administrated rat. (E) LCB 18(9Me):3(4E,8E,10E);2OH) administrated rat.

Figure S5.

(A) Ceramides and hexosylceramides with an LCB 18:2(4E,8Z);2OH moiety

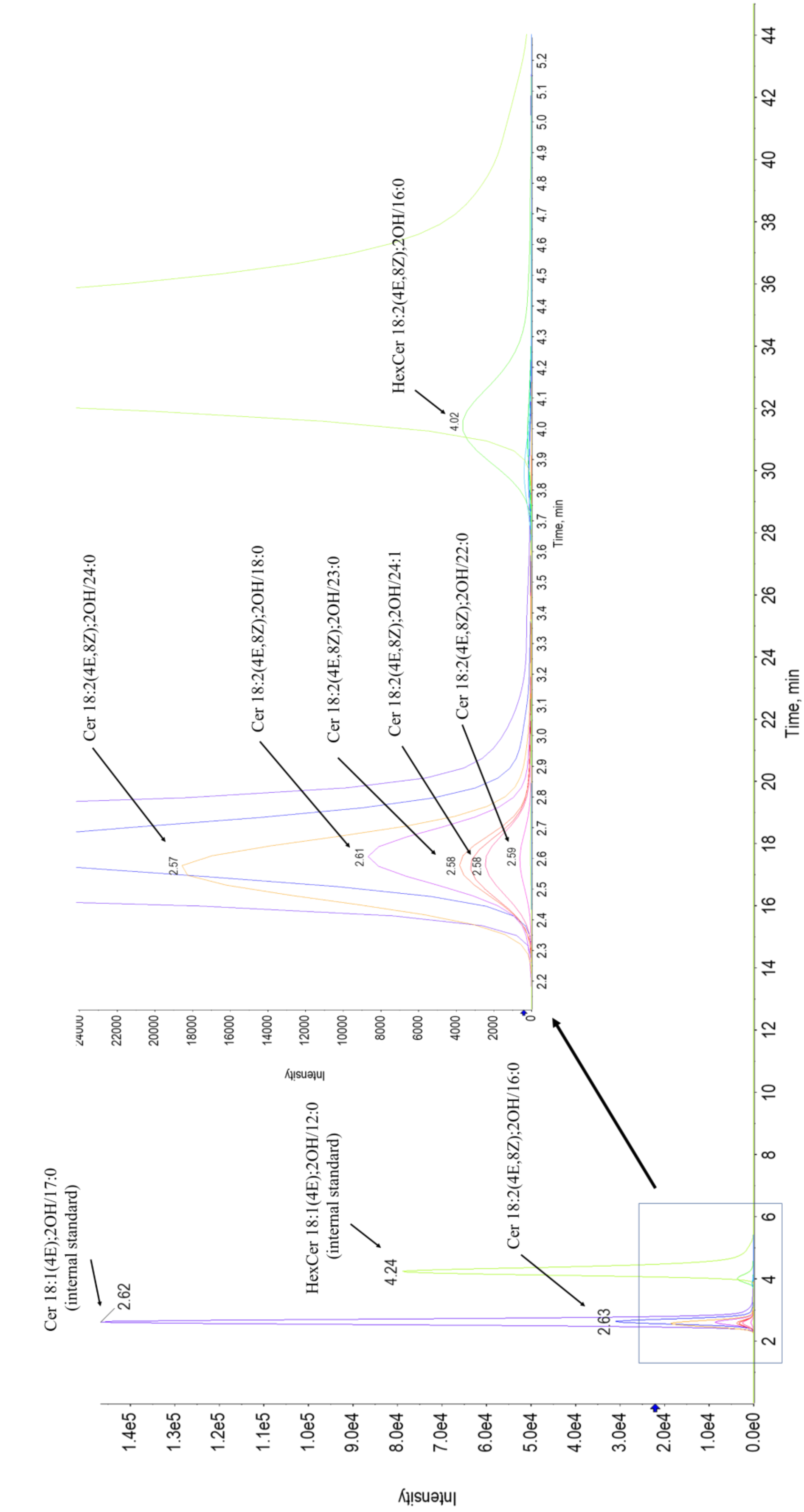

Figure S5. Continue.

(B) Ceramides and hexosylceramides with an LCB 18:2(4E,8E);2OH moiety

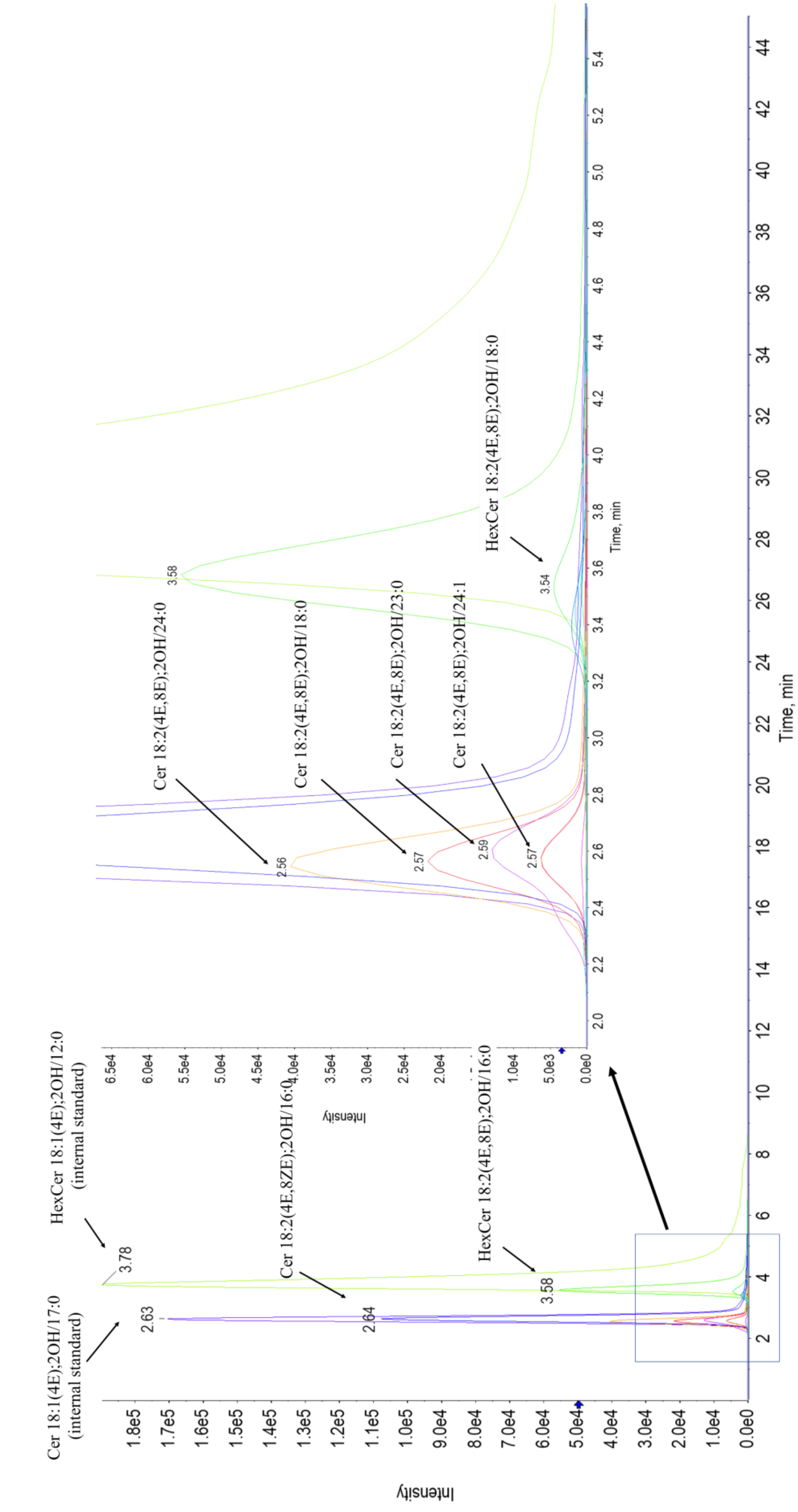

Figure S5. Continue.

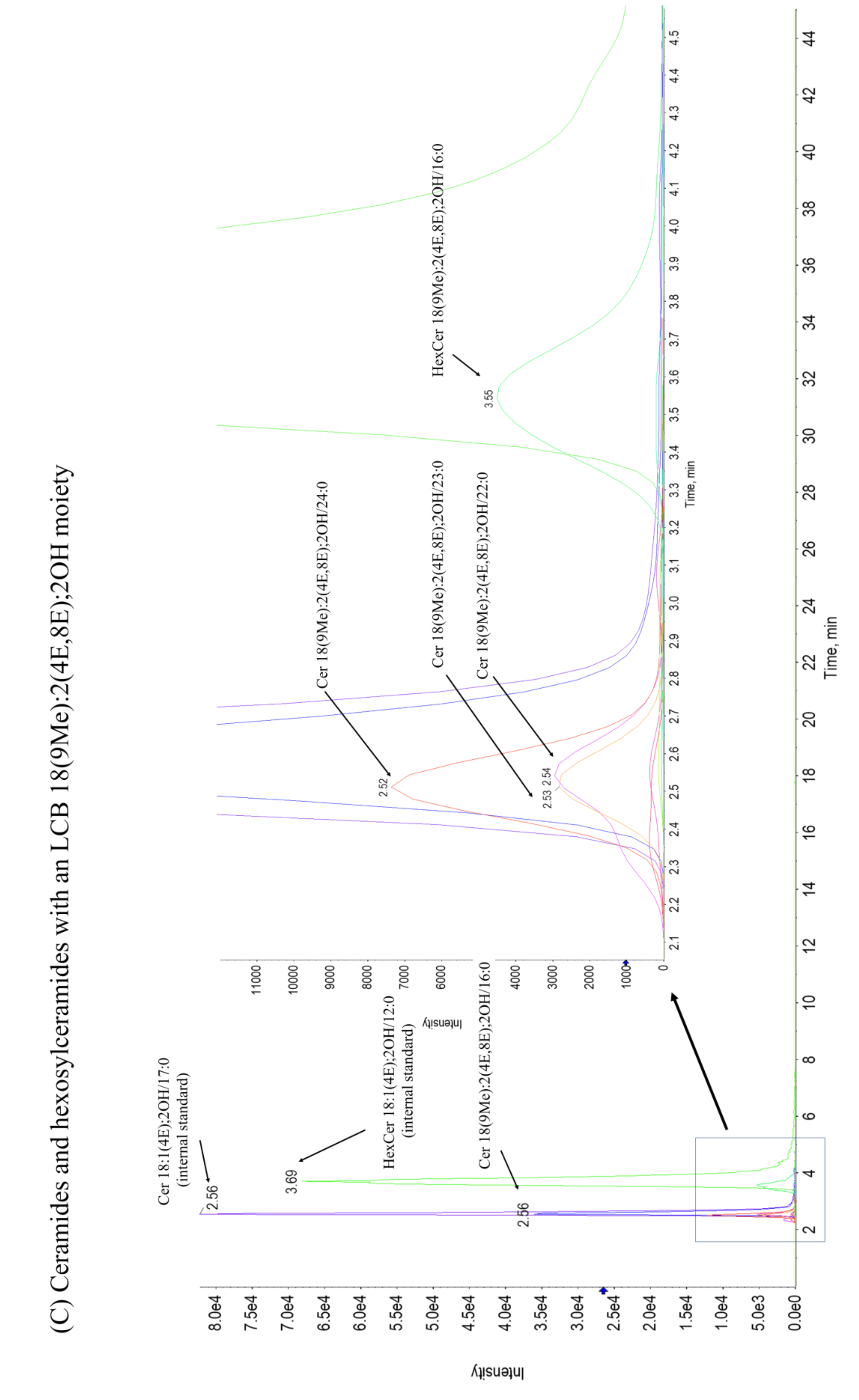

Figure S5. Continue.

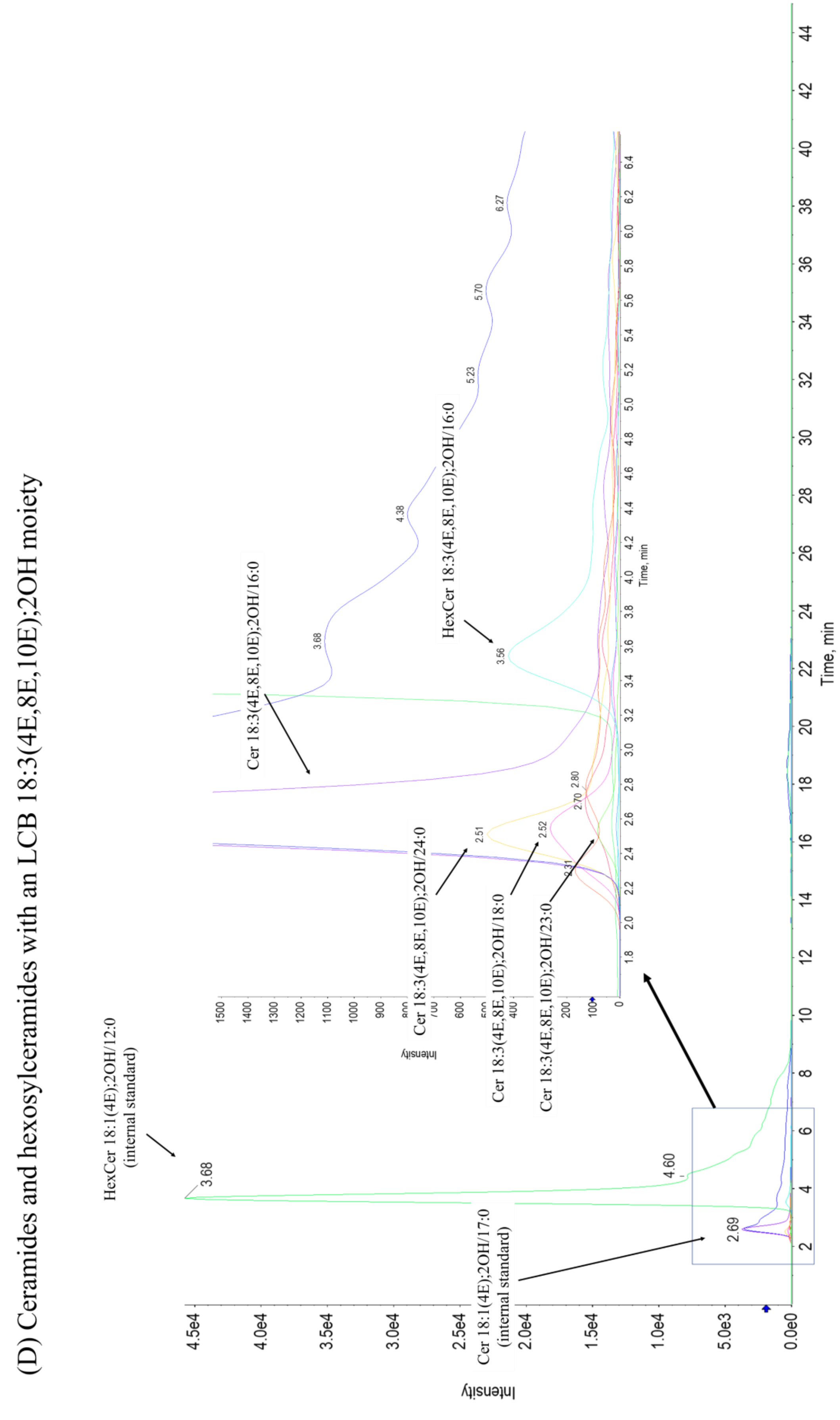

Figure S5. Continue.

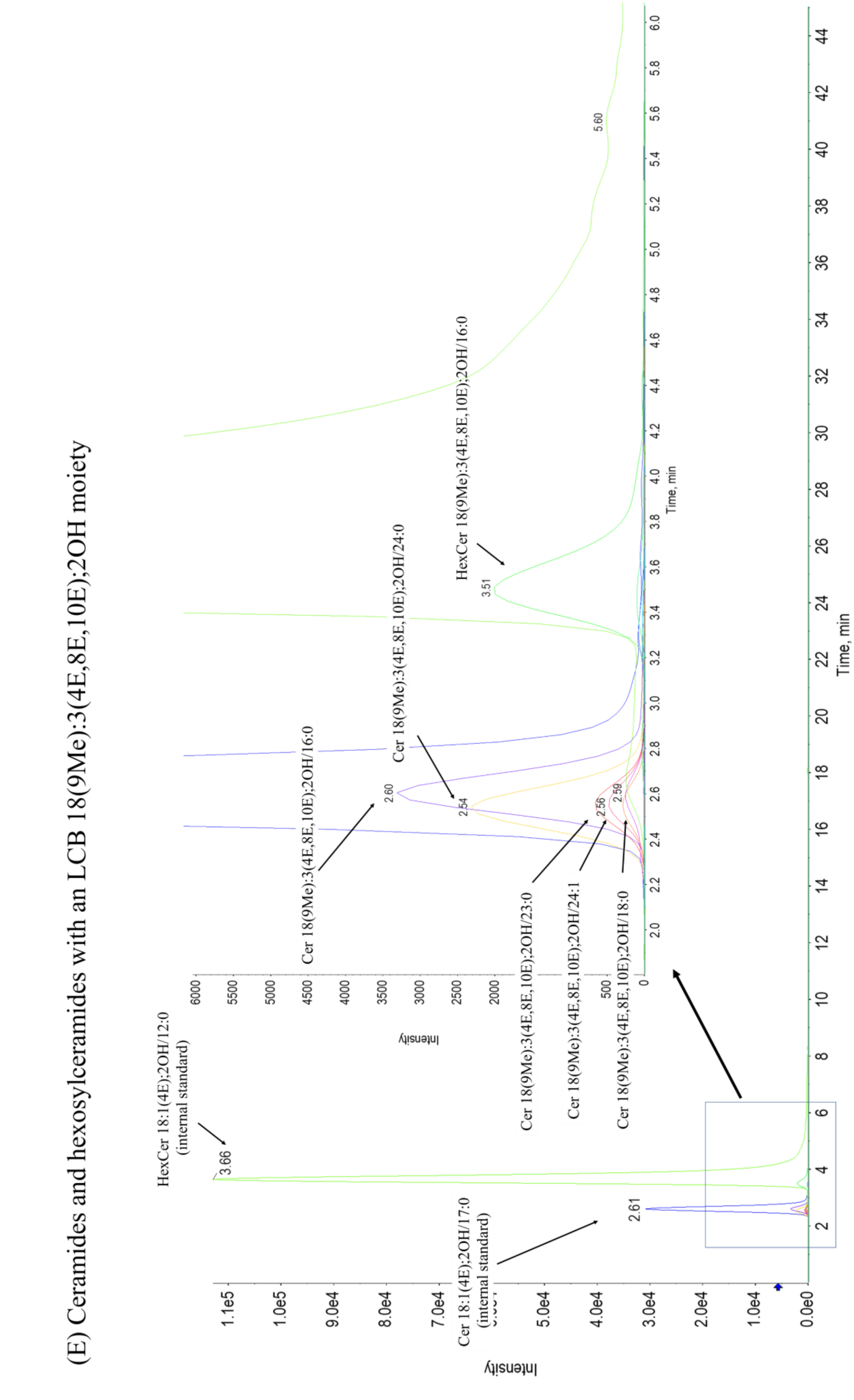

Figure S5: XIC of ceramides with atypical LCBs and hexosylceramides with atypical LCBs. Lipids were extracted from chyle of rats administrated nonmammalian LCBs. (A) LCB 18:2(4E,8Z);2OH administrated rat. (B) LCB 18:2(4E,8E);2OH administrated rat. (C) LCB 18:3(4E,8E,10E);2OH administrated rat. (D) LCB 18(9Me):2(4E,8Z);2OH administrated rat. (E) LCB 18(9Me):3(4E,8E,10E);2OH) administrated rat.

Figure S6.

(A) ceramides with an LCB 18:2(4E,8Z);2OH moiety

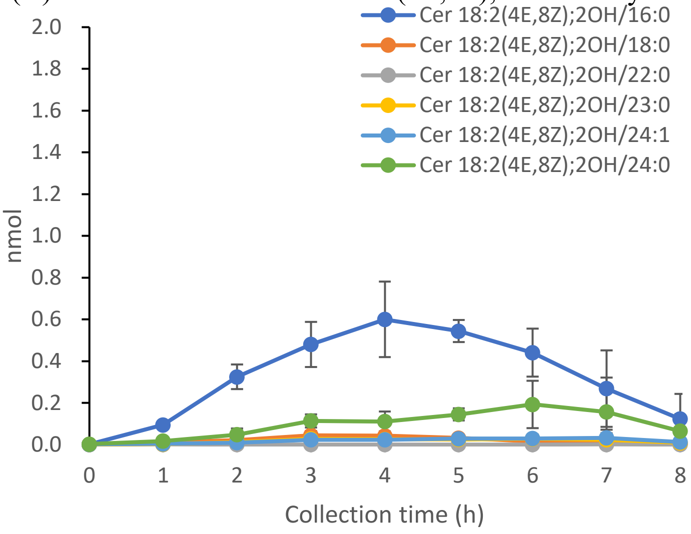

(B) ceramides with an LCB 18:2(4E,8E);2OH moiety

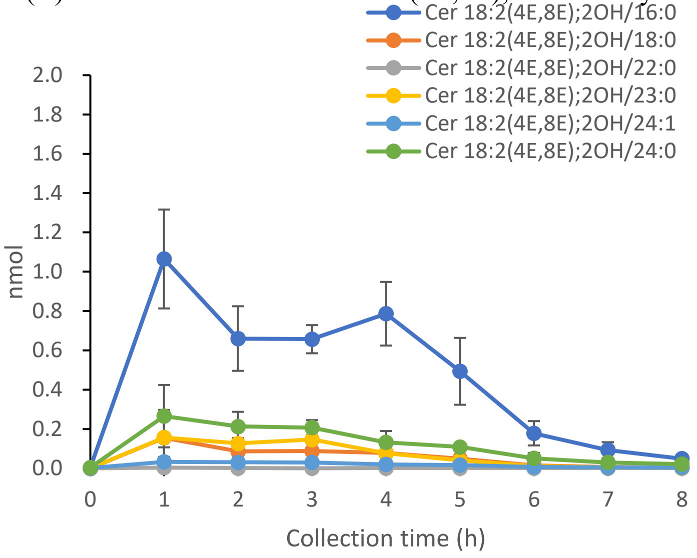

(C) ceramides with an LCB 18:3(4E,8E,10E);2OH moiety

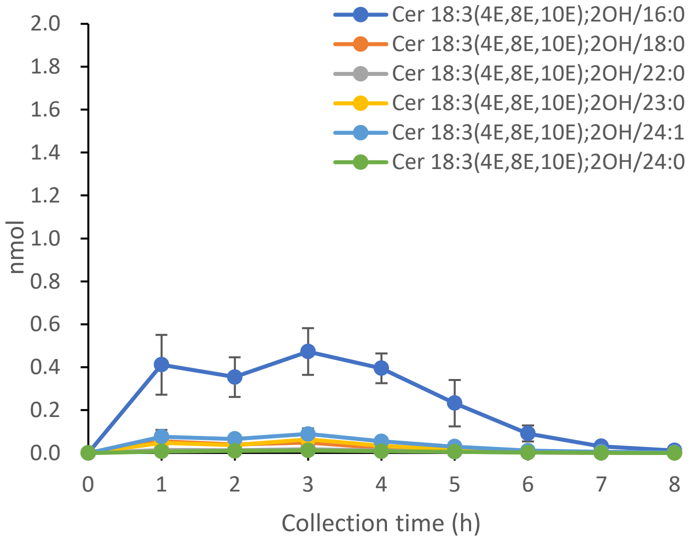

(D) ceramides with an LCB 18(9Me):2(4E,8Z);2OH

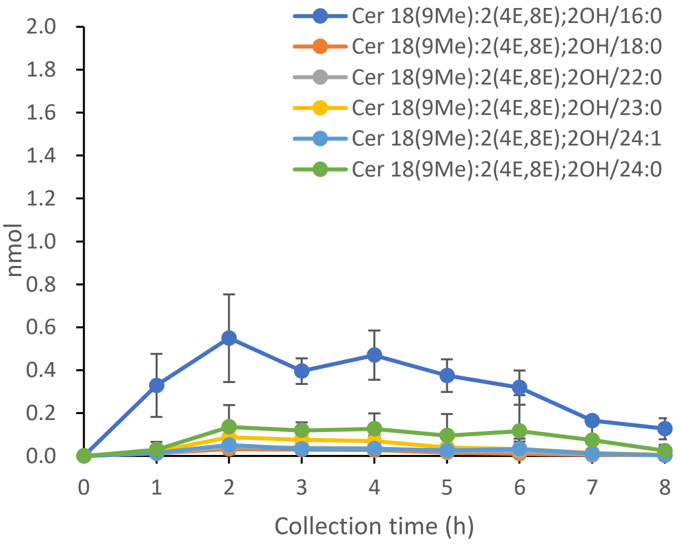

(E) ceramides with an LCB 18(9Me):3(4E,8E,10E);2OH

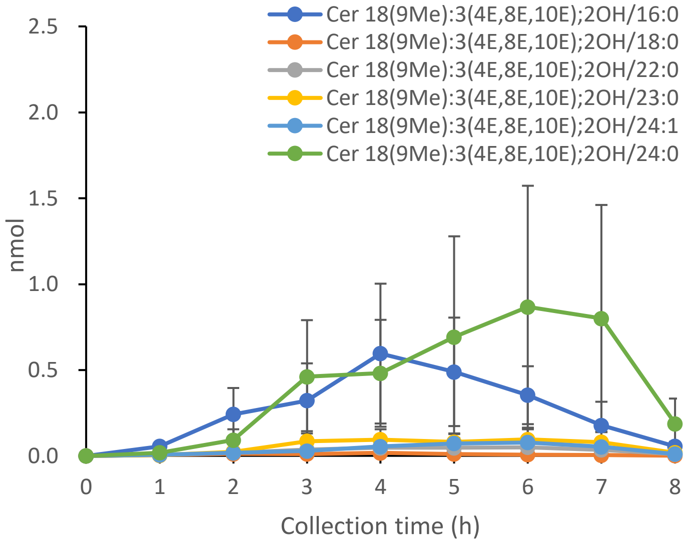

Figure S6: Changes in the amount of ceramides species (A) ceramides with an LCB 18:2(4E,8Z);2OH moiety, (B) ceramides with an LCB 18:2(4E,8E);2OH moiety, (C) ceramides with an LCB 18:3(4E,8E,10E);2OH moiety, (D) ceramides with an LCB 18(9Me):2(4E,8Z);2OH moiety and (E) ceramides with an LCB 18(9Me):3(4E,8E,10E);2OH moiety in the chyle of rats following enteral administration of long-chain bases.

Figure S7.

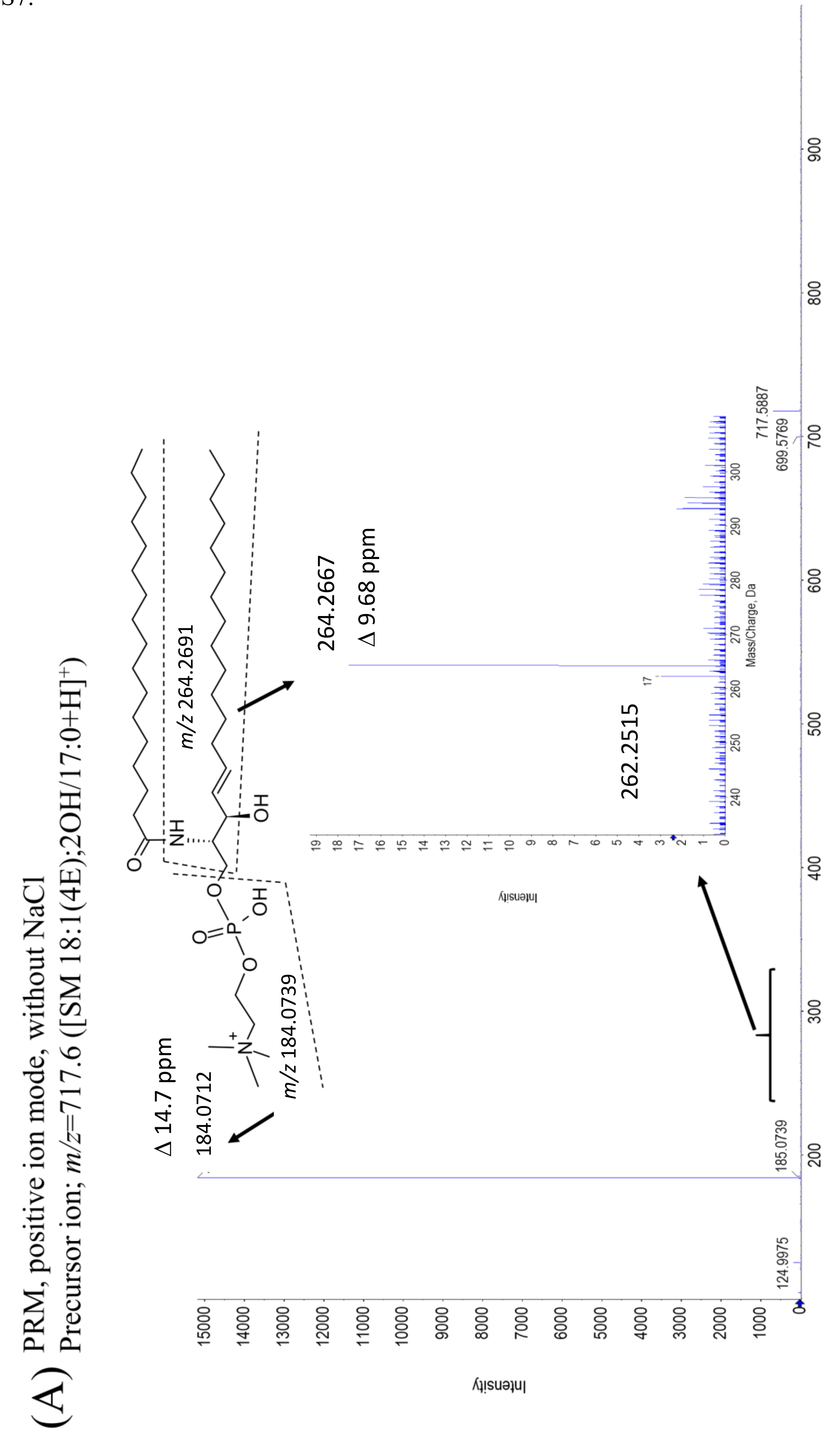
(A) PRM, positive ion mode, without NaCl
Precursor ion; m/z=717.6 ([SM 18:1(4E);2OH/17:0+H]+)
Δ 14.7 ppm
184.0712
m/z 184.0739
m/z 264.2691
264.2667
Δ 9.68 ppm
262.2515
124.9975
185.0739
699.5769
717.5887
Intensity
Mass/Charge, Da

Figure S7. Continue.

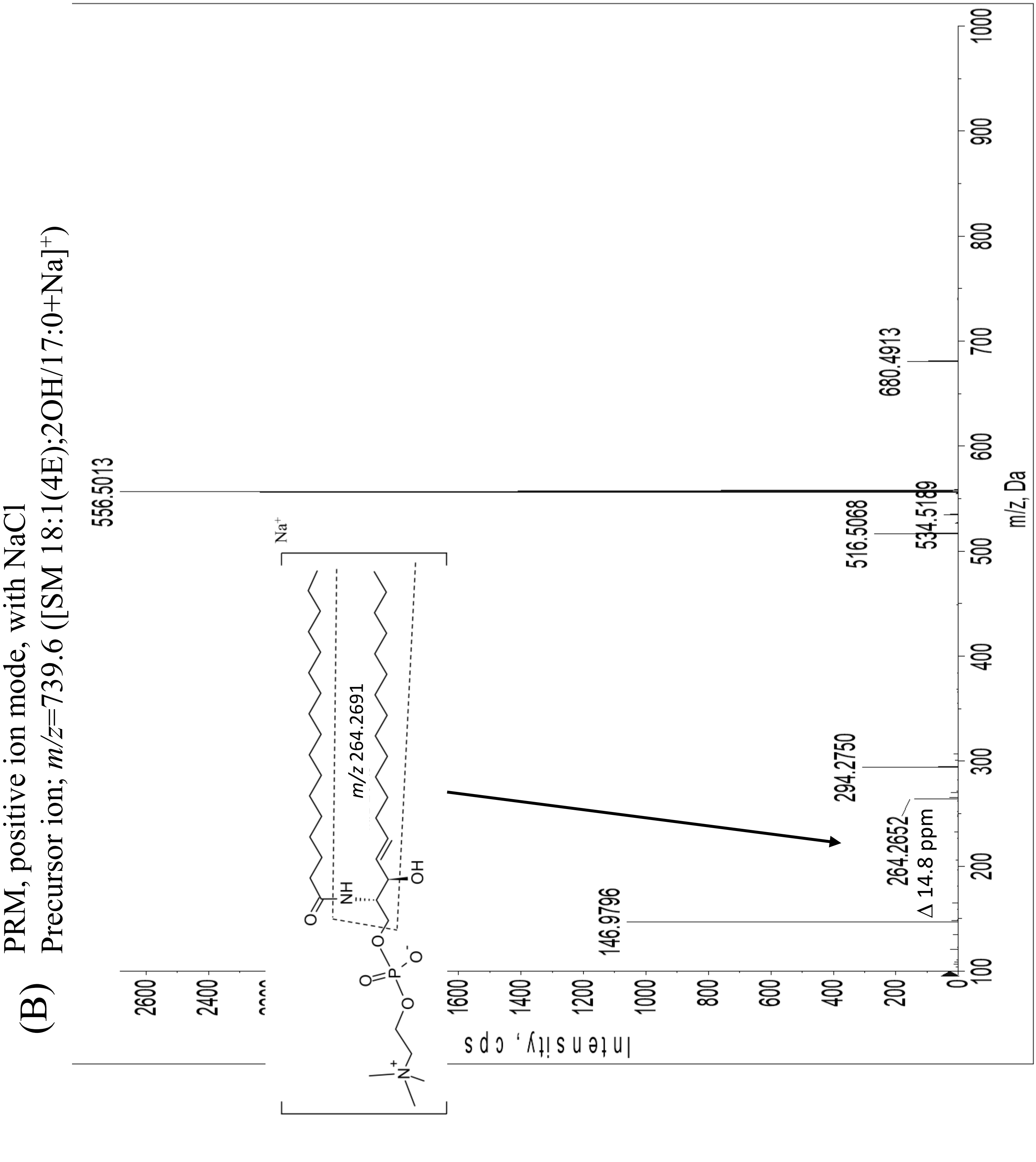

Figure S7. Continue.

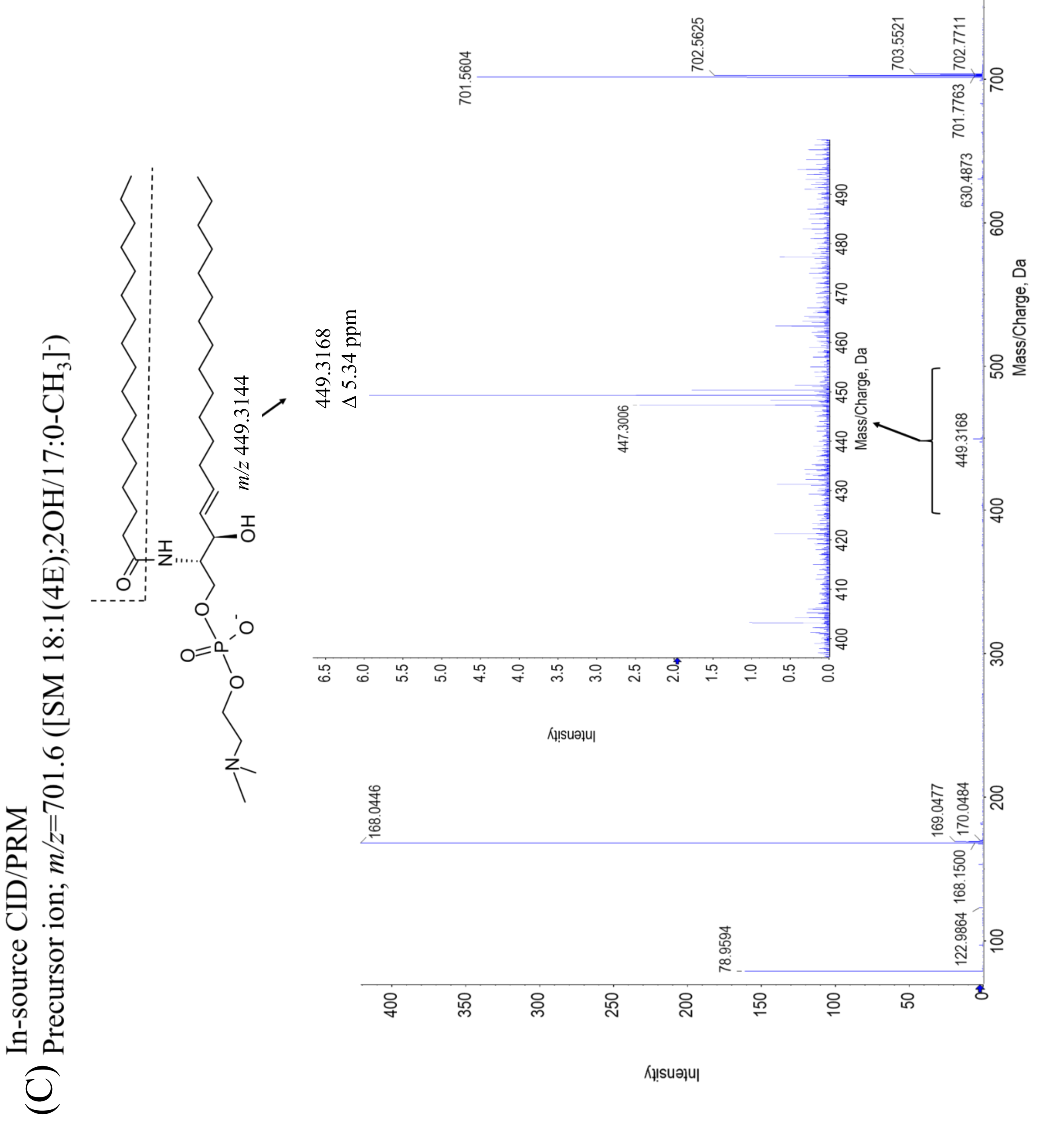

Figure S7: Product ion spectra of SM by ESI-positive mode (A,B) or demethylated SM by ESI-negative mode (C). (A) Product ion spectra of protonated SM 18:1(4E);2OH/17:0 on NaCl-free conditions. (B) Product ion spectra of sodium adducted SM 18:1(4E);2OH/17:0 on NaCl-present conditions. (C) Product ion spectra of demethylated SM 18:1(4E);2OH/17:0.

Figure S8.

(A) TOF survey scan of LCB 18:2(4E,8Z);2OH administered group

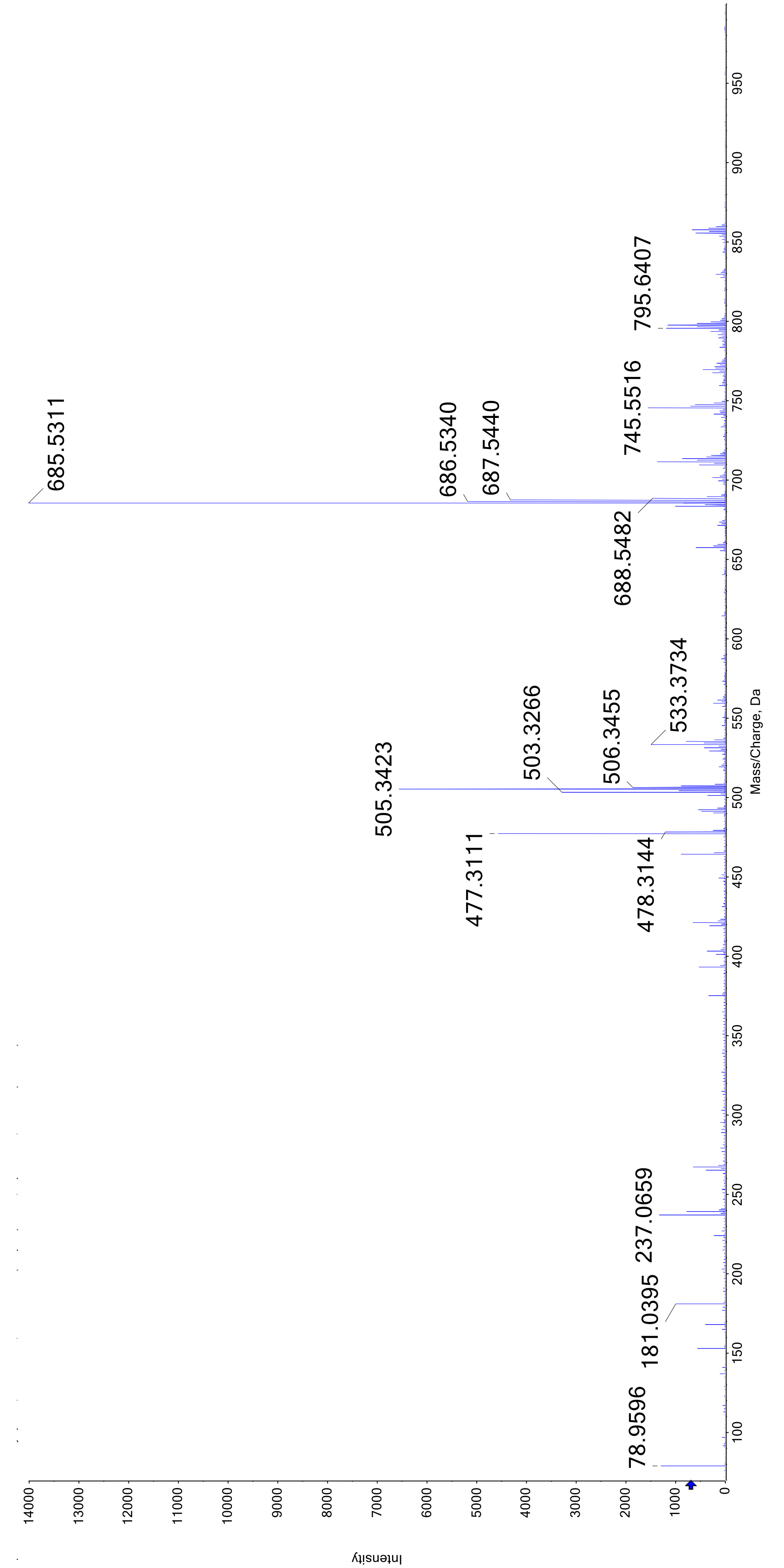

Figure S8. Continue.

(B) TOF survey scan of LCB 18:2(4E,8E);2OH administered group

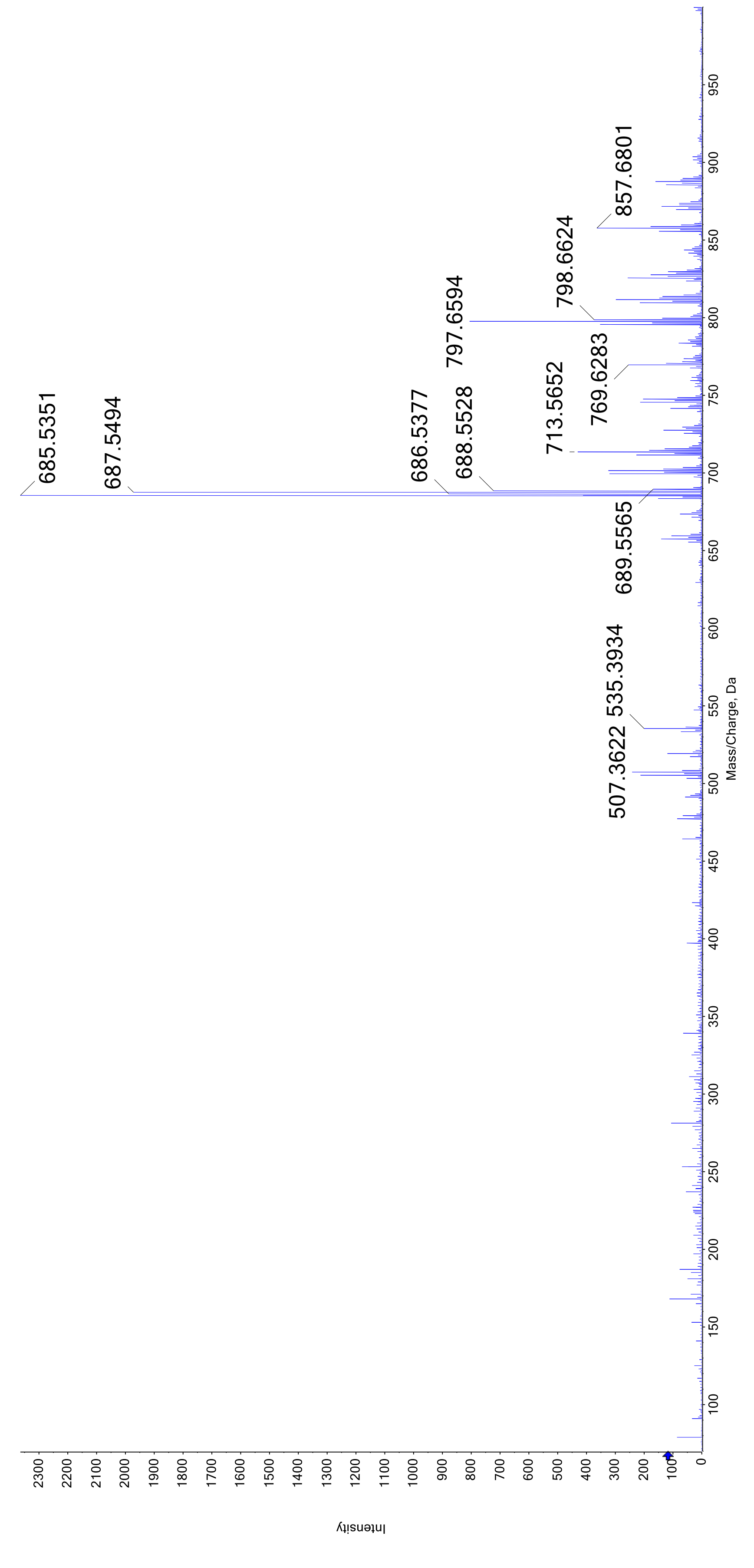

Figure S8. Continue.

(C) TOF survey scan of LCB 18:3(4E,8E,10E);2OH administered group

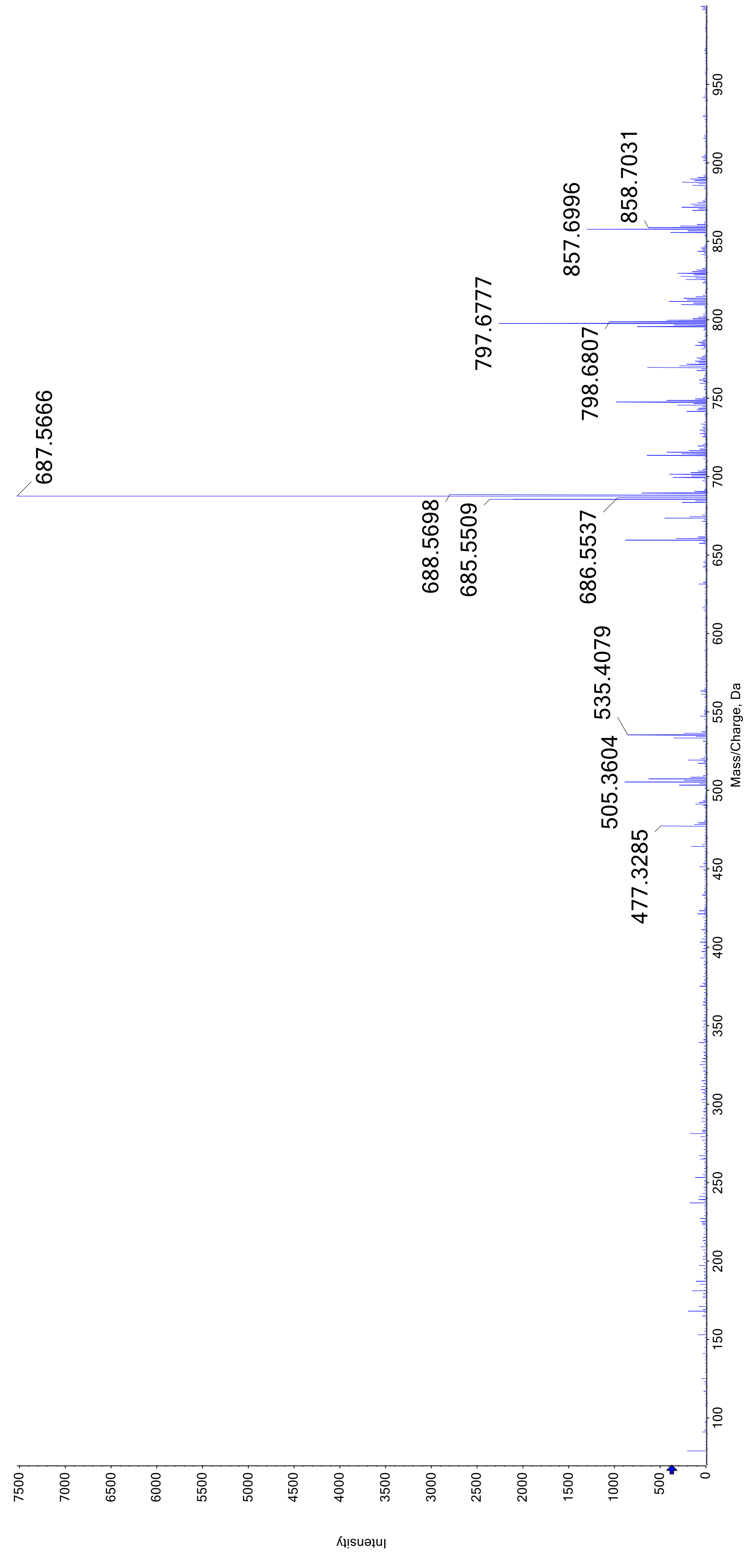

Figure S8. Continue.

(D) TOF survey scan of LCB 18(9Me):2(4E,8E);2OH administered group

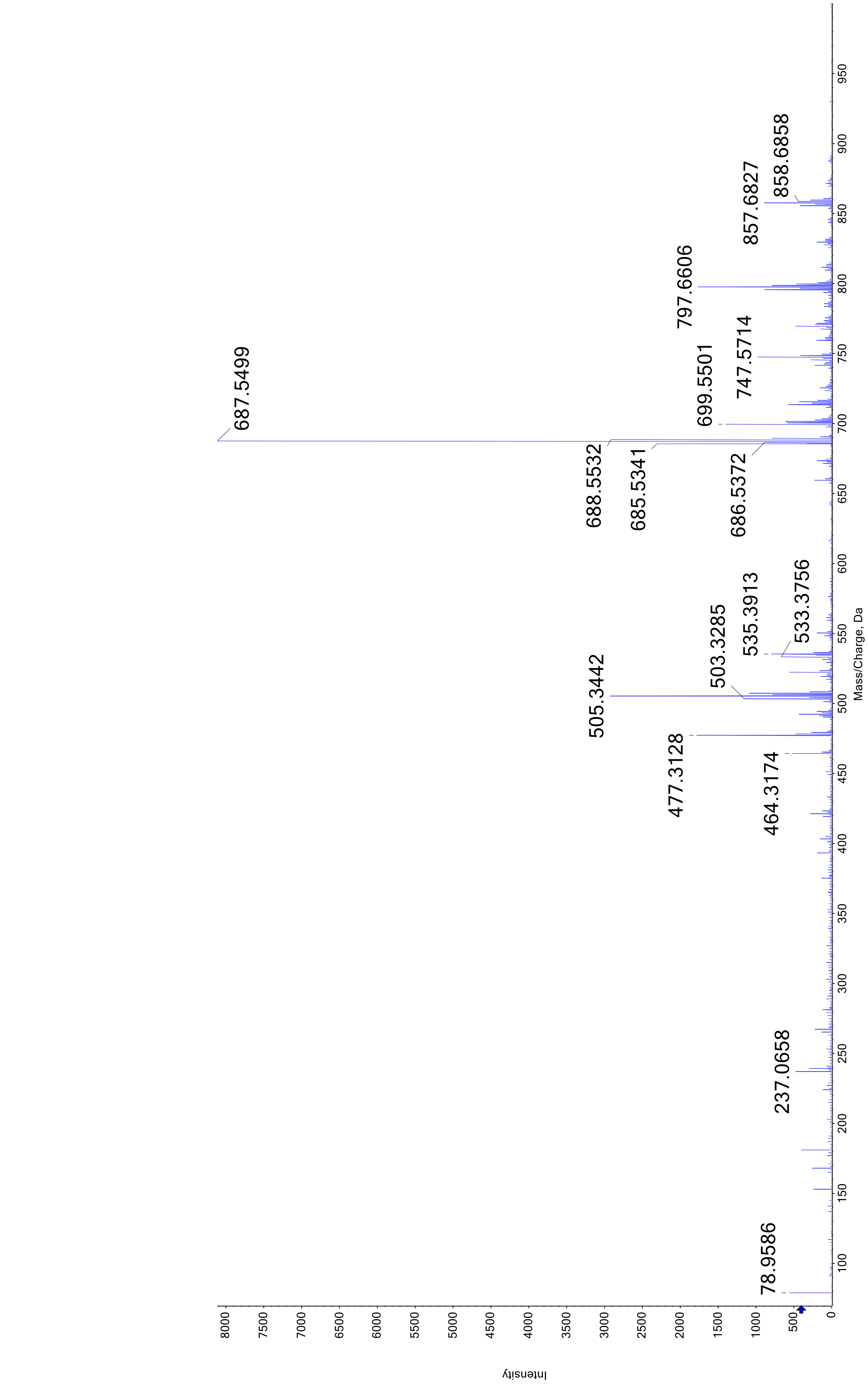

Figure S8. Continue.

(E) TOF survey scan of LCB 18(9Me):3(4E,8E,10E);2OH administered group

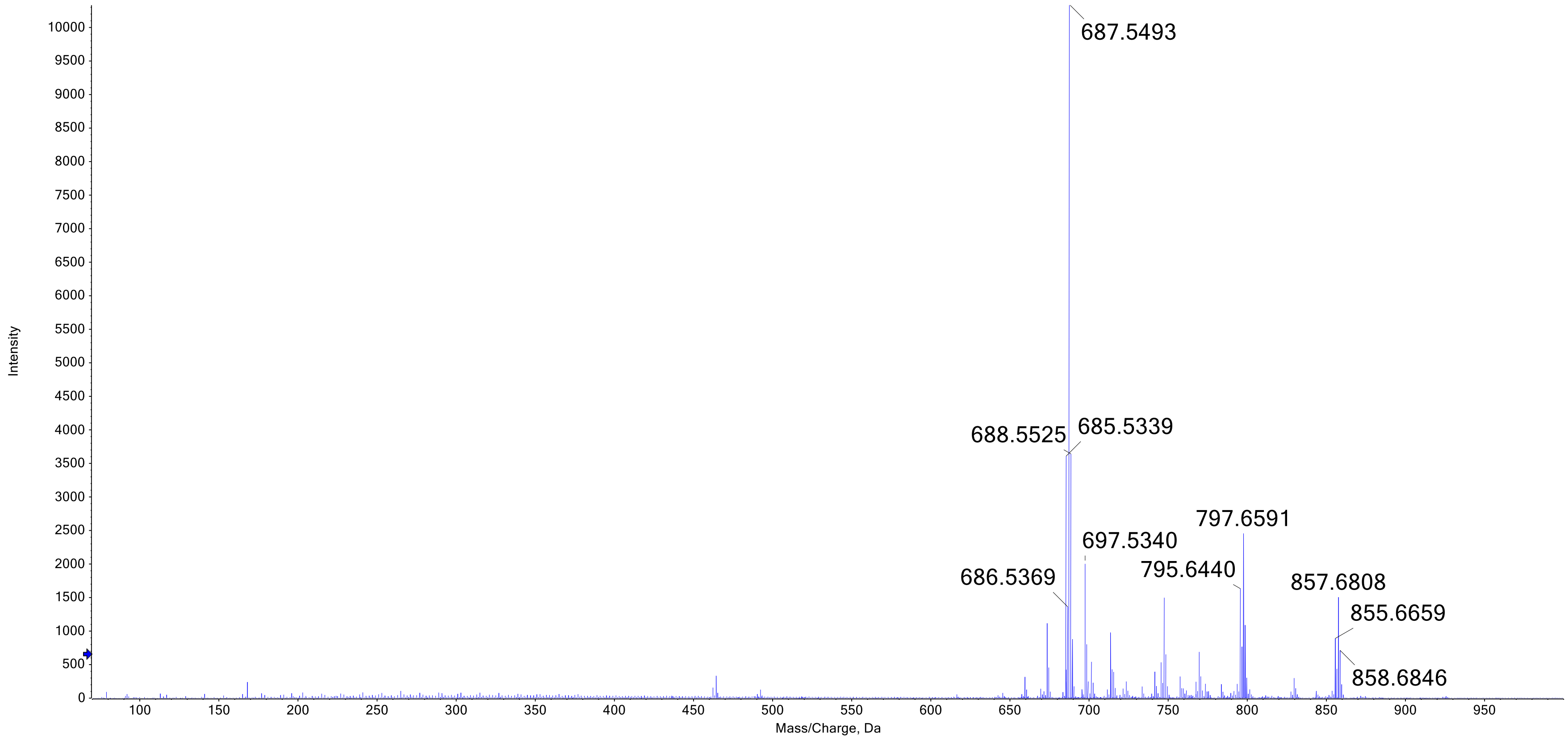

Figure S8. Continue.

(F) TOF MS based XIC of LCB 18:2(4E,8Z);2OH administered group

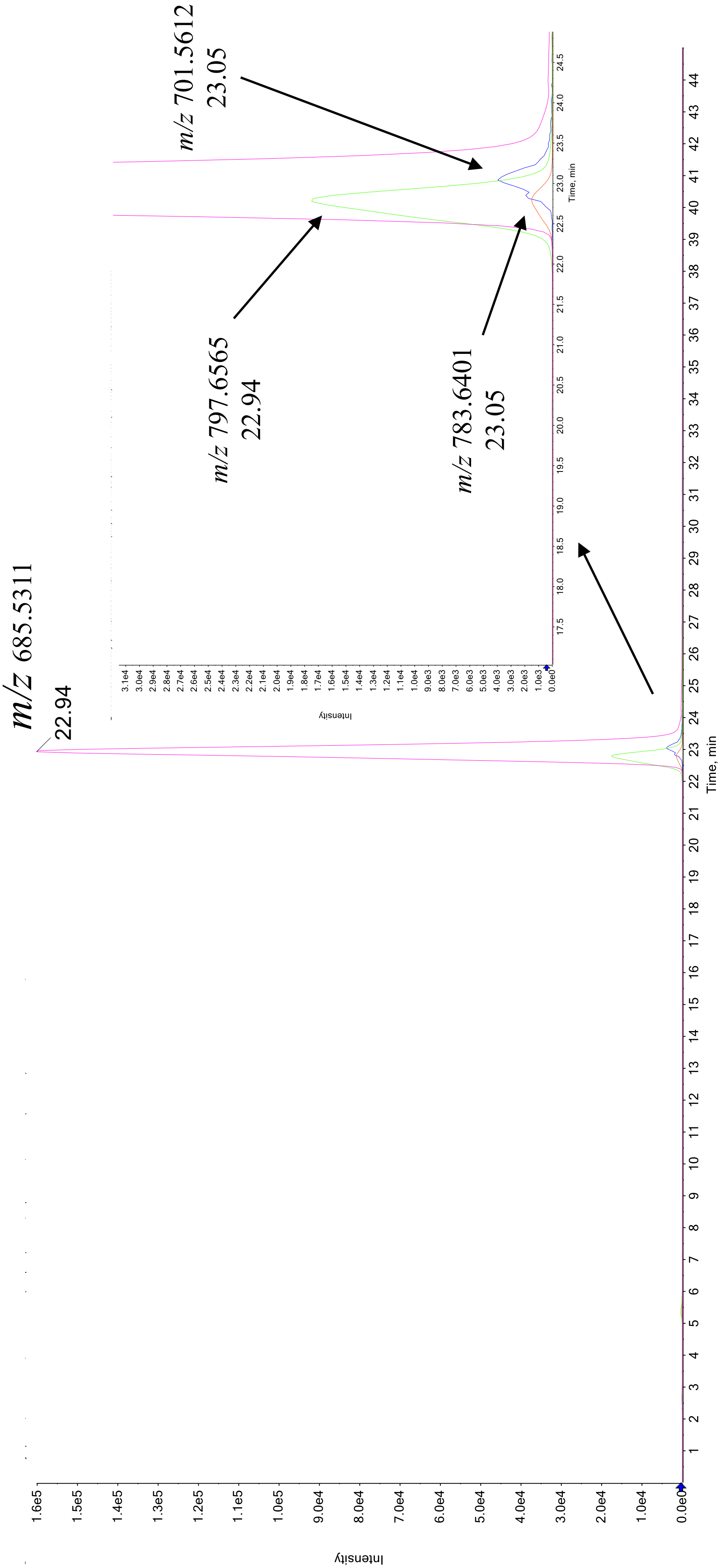

Figure S8. Continue.

(G) TOF MS based XIC of LCB 18:2(4E,8E);2OH administered group

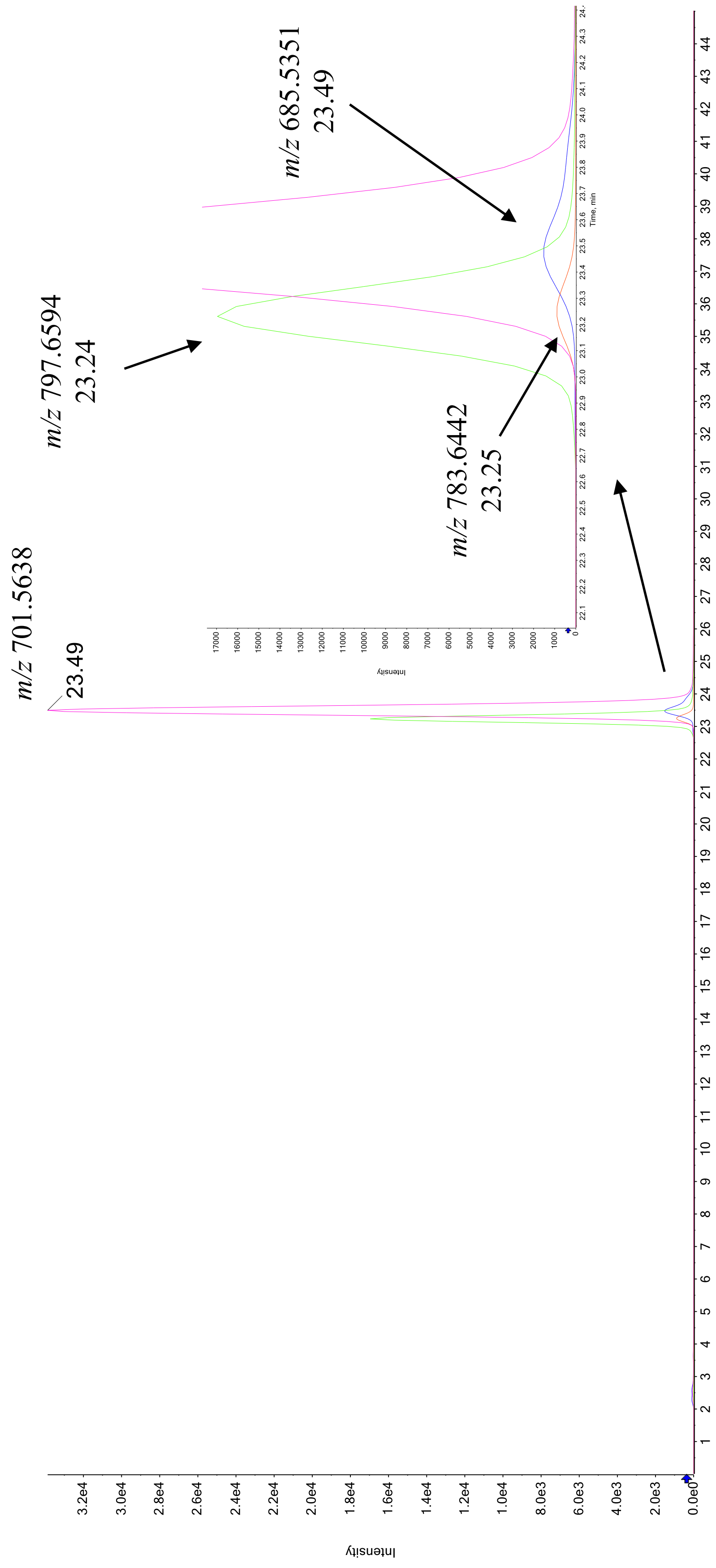

Figure S8. Continue.

(H) TOF MS based XIC of LCB 18:3(4E,8E,10E);2OH administered group

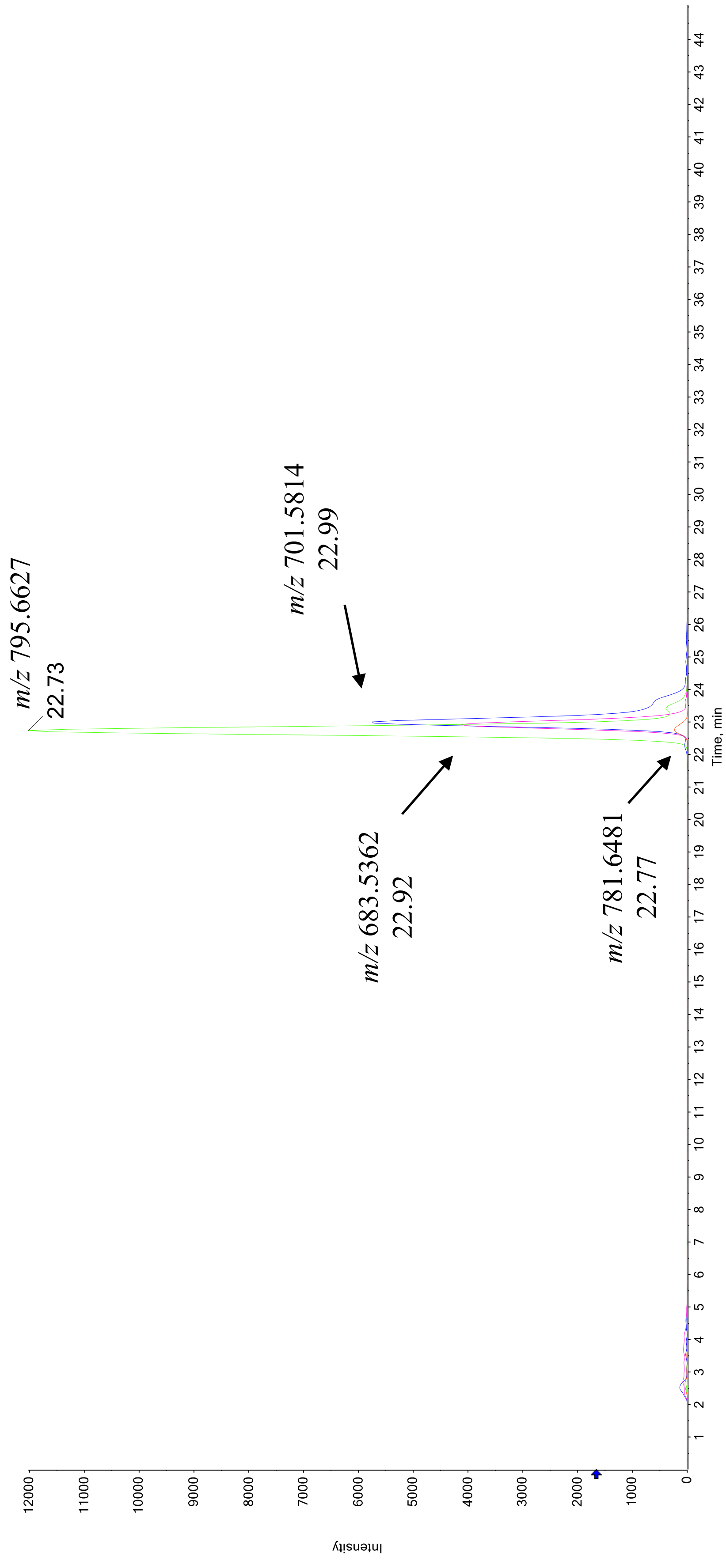

Figure S8. Continue.

(I) TOF MS based XIC of LCB 18(9Me):2(4E,8E);2OH administered group

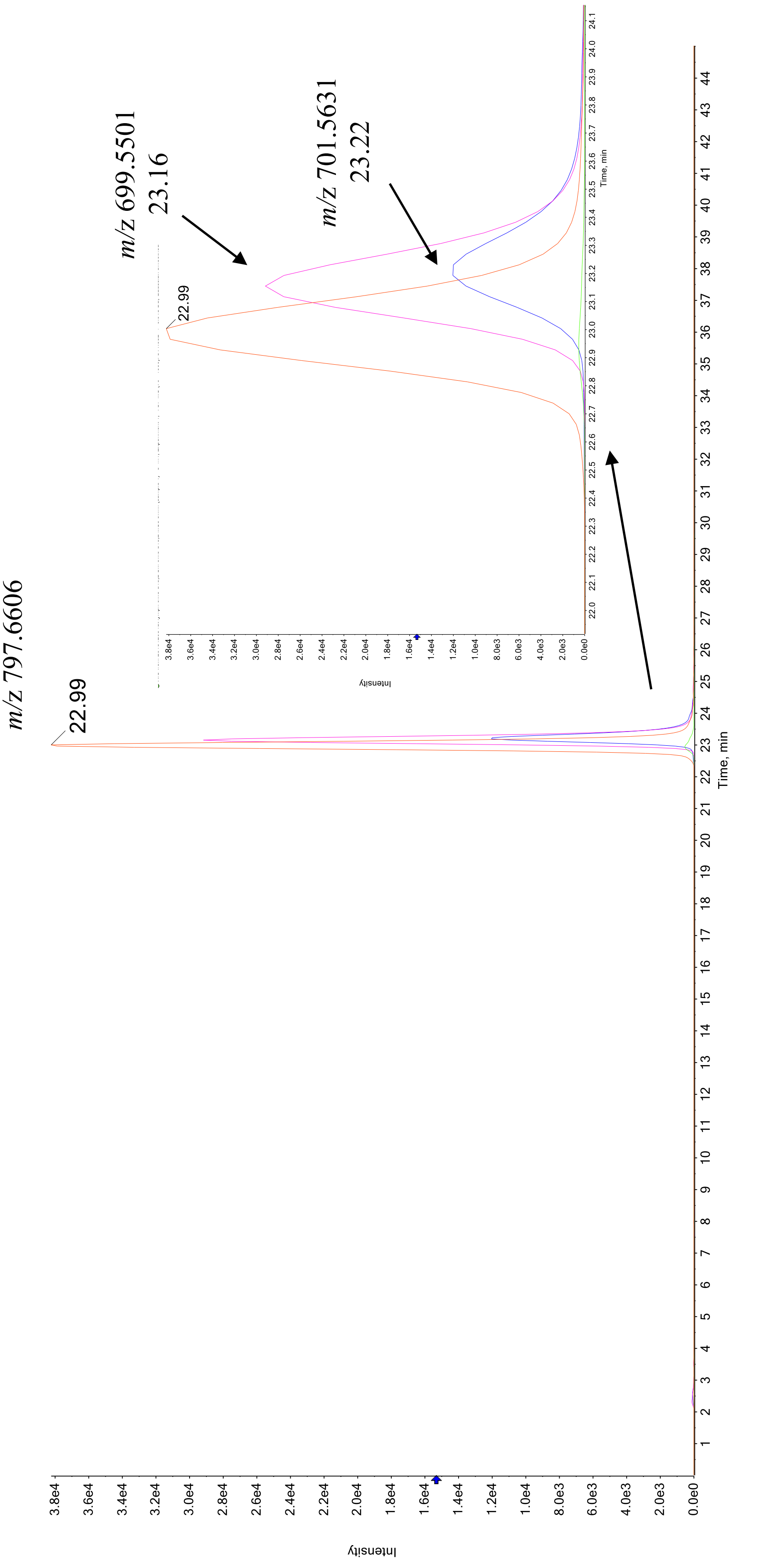

Figure S8. Continue.

(J) TOF MS based XIC of LCB 18(9Me):3(4E,8E,10E);2OH administered group

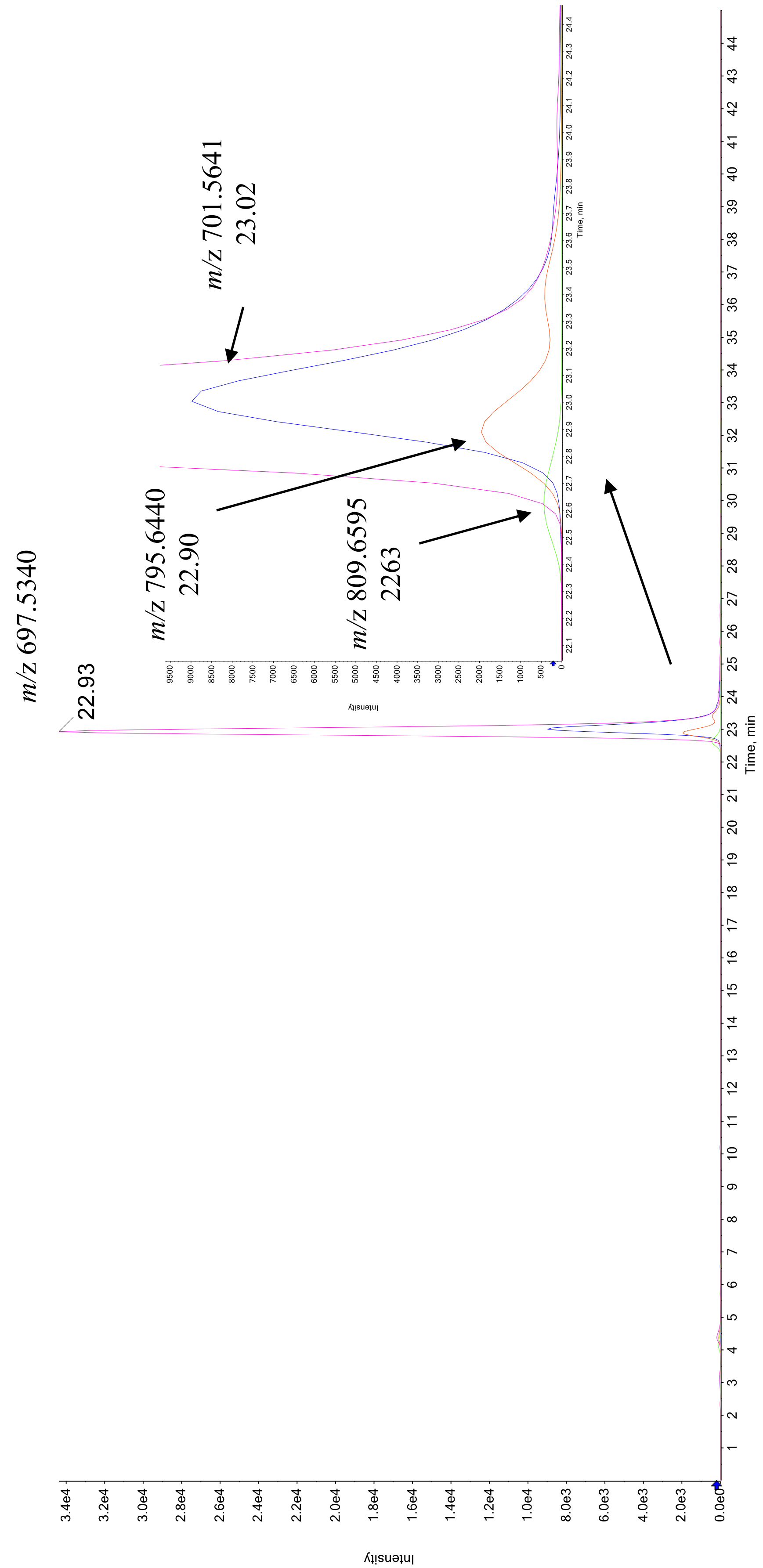

Figure S8: TOF survey scan spectra of SMs fraction and XICs which speculated to demethylated SM by insource CID on ESI-negative mode. (A) TOF survey scan spectra of lipids extracted from LCB 18:2(4E,8Z);2OH administered rat chyle. (B) TOF survey scan spectra of lipids extracted from LCB 18:2(4E,8E);2OH administered rat chyle. (C) TOF survey scan spectra of lipids extracted from LCB 18:3(4E,8E,10E);2OH administered rat chyle. (D) TOF survey scan spectra of lipids extracted from LCB 18(9Me):2(4E,8E) administered rat chyle. (E) TOF survey scan spectra of lipids extracted from LCB 18(9Me):3(4E,8E,10E);2OH administered rat chyle. (F) XICs of m/z 685.5311, m/z 783.6401, m/z 797.6565 and m/z 701.5612. Since these ions were speculated to be demethylated SM 18:2(4E,8Z);2OH/16:0, demethylated SM 18:2(4E,8Z);2OH/23:0, demethylated SM 18:2(4E,8Z);2OH/24:0 and demethylated SM 18:1(4E);2OH/17:0, respectively, to obtain product ion spectrum CID experiments were carried out. (G) XICs of m/z 685.5351, m/z 783.6442, m/z 797.6594 and m/z 701.5638. Since these ions were speculated to be demethylated SM 18:2(4E,8E);2OH/16:0, demethylated SM 18:2(4E,8E);2OH/23:0, demethylated SM 18:2(4E,8E);2OH/24:0 and demethylated SM 18:1(4E);2OH/17:0, respectively, to obtain product ion spectrum CID experiments were carried out. (H) XICs of m/z 683.5362, m/z 781.6481, m/z 795.6627 and m/z 701.5814. Since these ions were speculated to be demethylated SM 18:3(4E,8E,10E);2OH/16:0, demethylated SM 18:3(4E,8E10E);2OH/23:0, demethylated SM 18:3(4E,8E10E);2OH/24:0 and demethylated SM 18:1(4E);2OH/17:0, respectively, to obtain product ion spectrum CID experiments were carried out. (I) XICs of m/z 699.5501, m/z 797.6606, m/z 811.6827 and m/z 701.5631. Since these ions were speculated to be demethylated SM 18(9Me):2(4E,8E);2OH/16:0, demethylated SM 18(9Me):2(4E,8E);2OH/23:0, demethylated SM 18(9Me):2(4E,8E);2OH/24:0 and demethylated SM 18:1(4E);2OH/17:0, respectively, to obtain product ion spectrum CID experiments were carried out. (J) XICs of m/z 697.5340, m/z 795.6440, m/z 809.6595 and m/z 701.5641. Since these ions were speculated to be demethylated SM 18(9Me):3(4E,8E,10E);2OH/16:0, demethylated SM 18(9Me):3(4E,8E10E);2OH/23:0, demethylated SM 18(9Me):3(4E,8E10E);2OH/24:0 and demethylated SM 18:1(4E);2OH/17:0, respectively, to obtain product ion spectrum CID experiments were carried out.

Figure S9.

(A) In-source CID/PRM
Precursor ion; m/z=685.5 ([SM 18:2(4E,8Z);2OH/16:0-CH_3]$^-$)

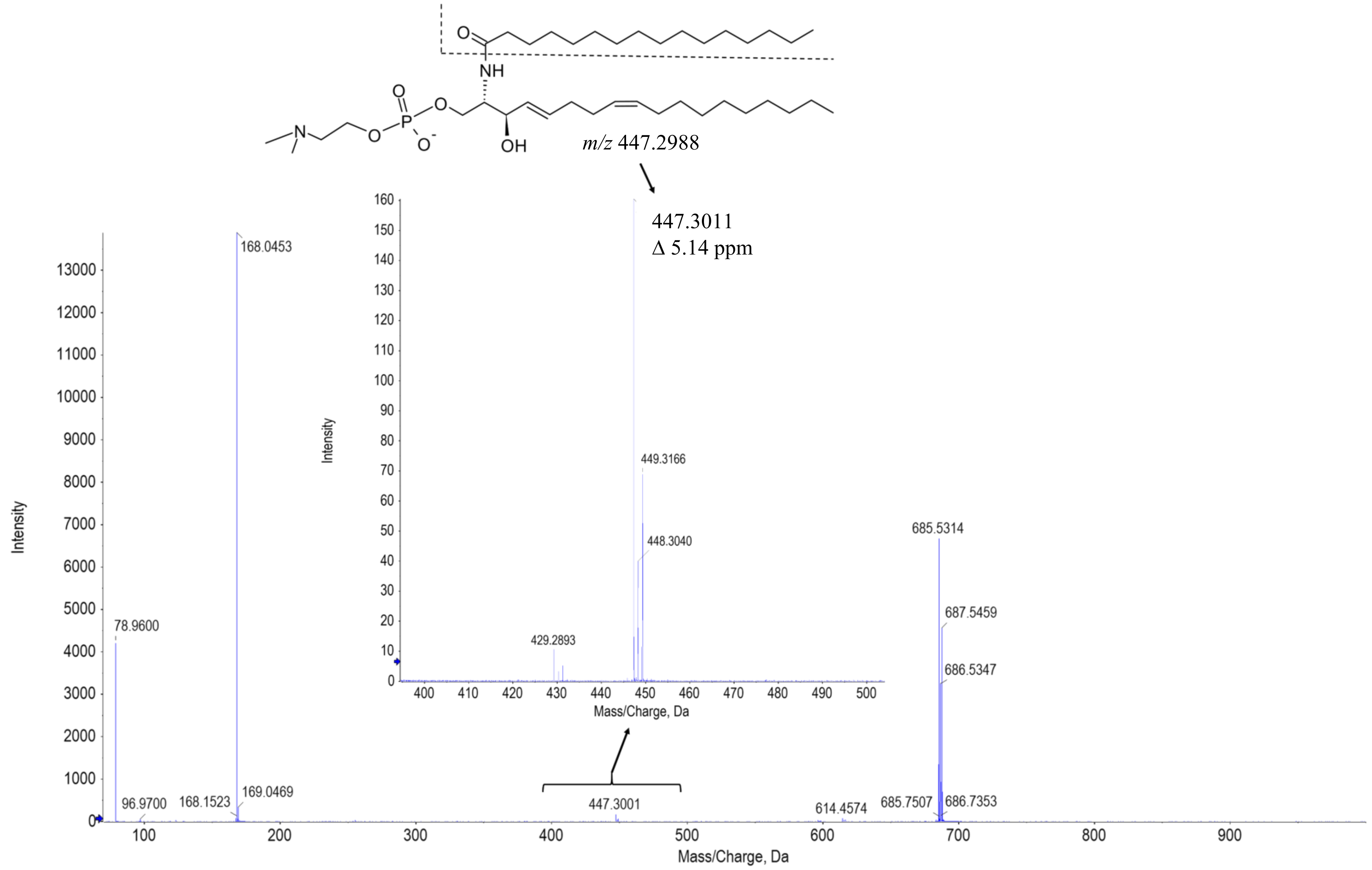

Figure S9. Continue.

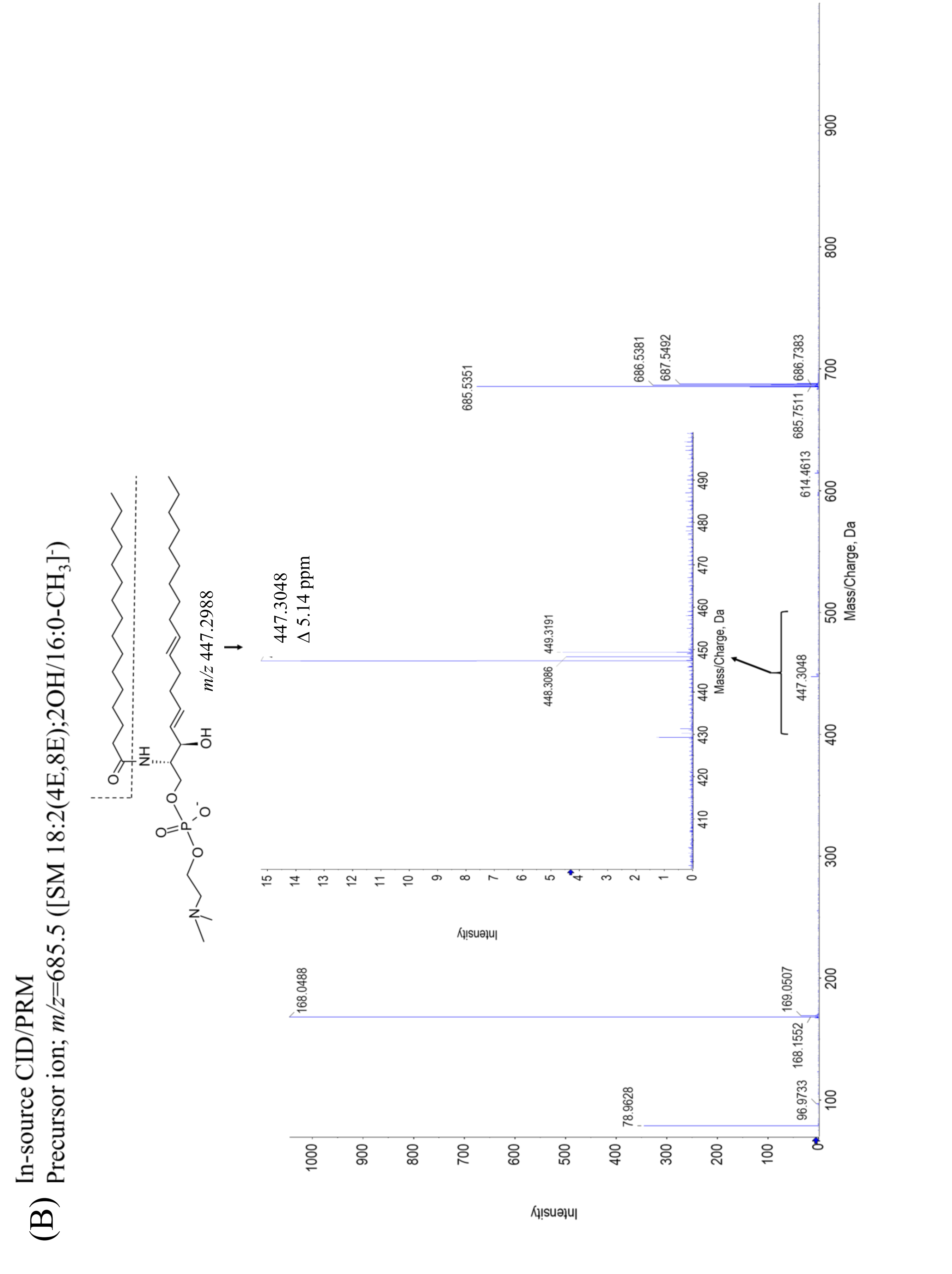

Figure S9. Continue.

(C) In-source CID/PRM
Precursor ion; m/z=685.5 ([SM 18(9Me):2(4E,8E);2OH/16:0-CH_3]$^-$)

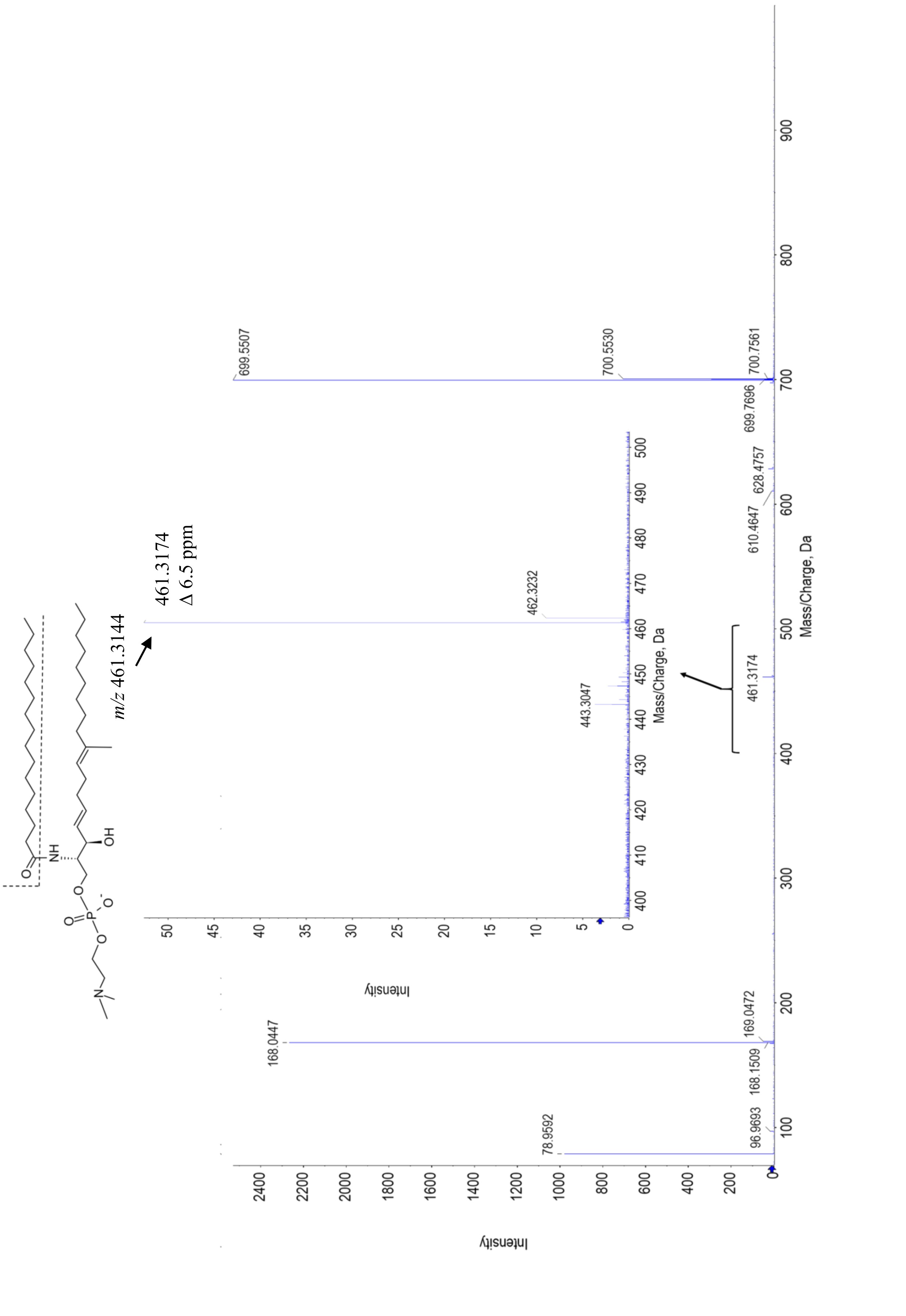

Figure S9. Continue.

(D) In-source CID/PRM
Precursor ion; m/z=683.5 ([SM 18:3(4E,8E,10E);2OH/16:0-CH_3]$^-$)

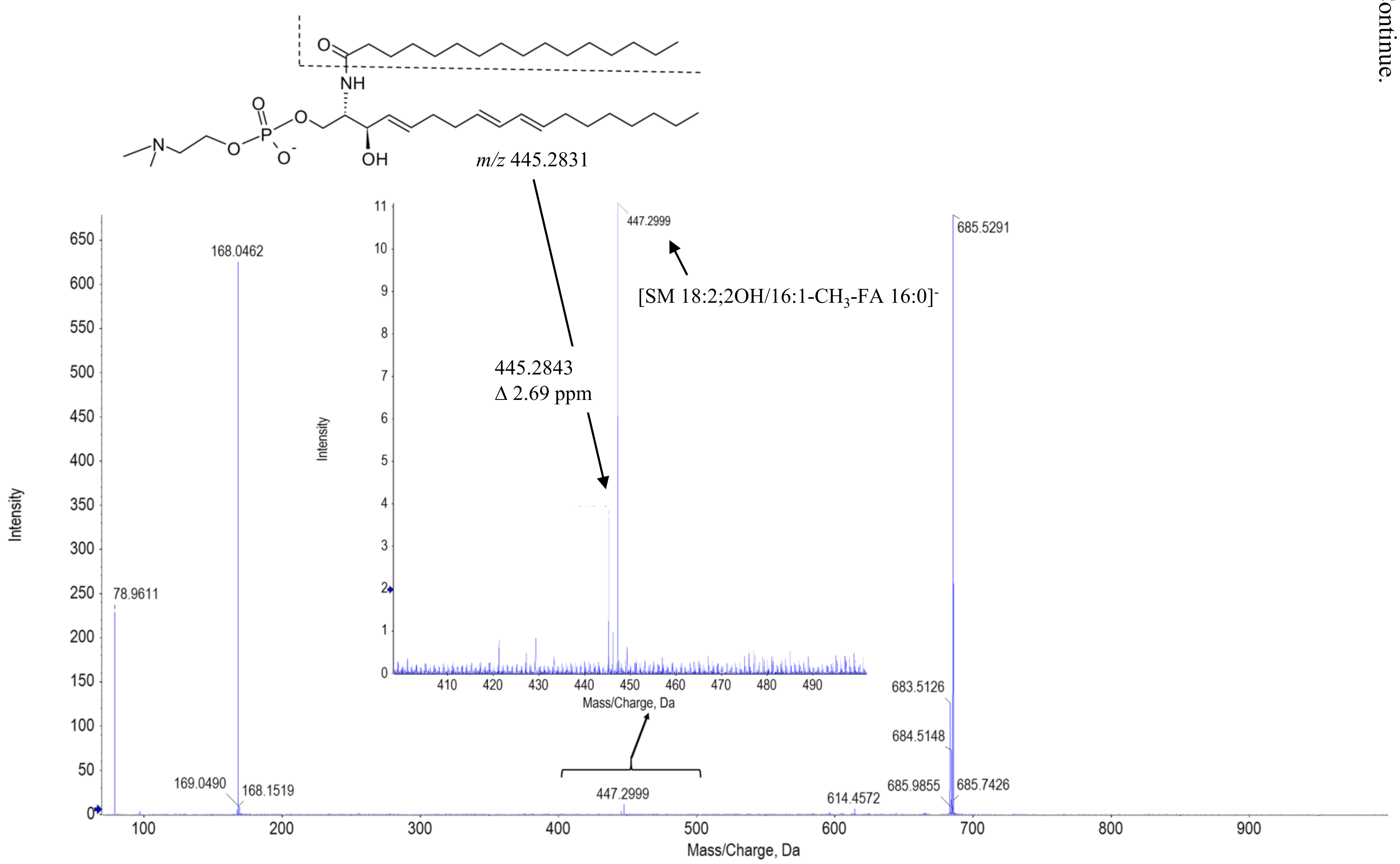

Figure S9. Continue.

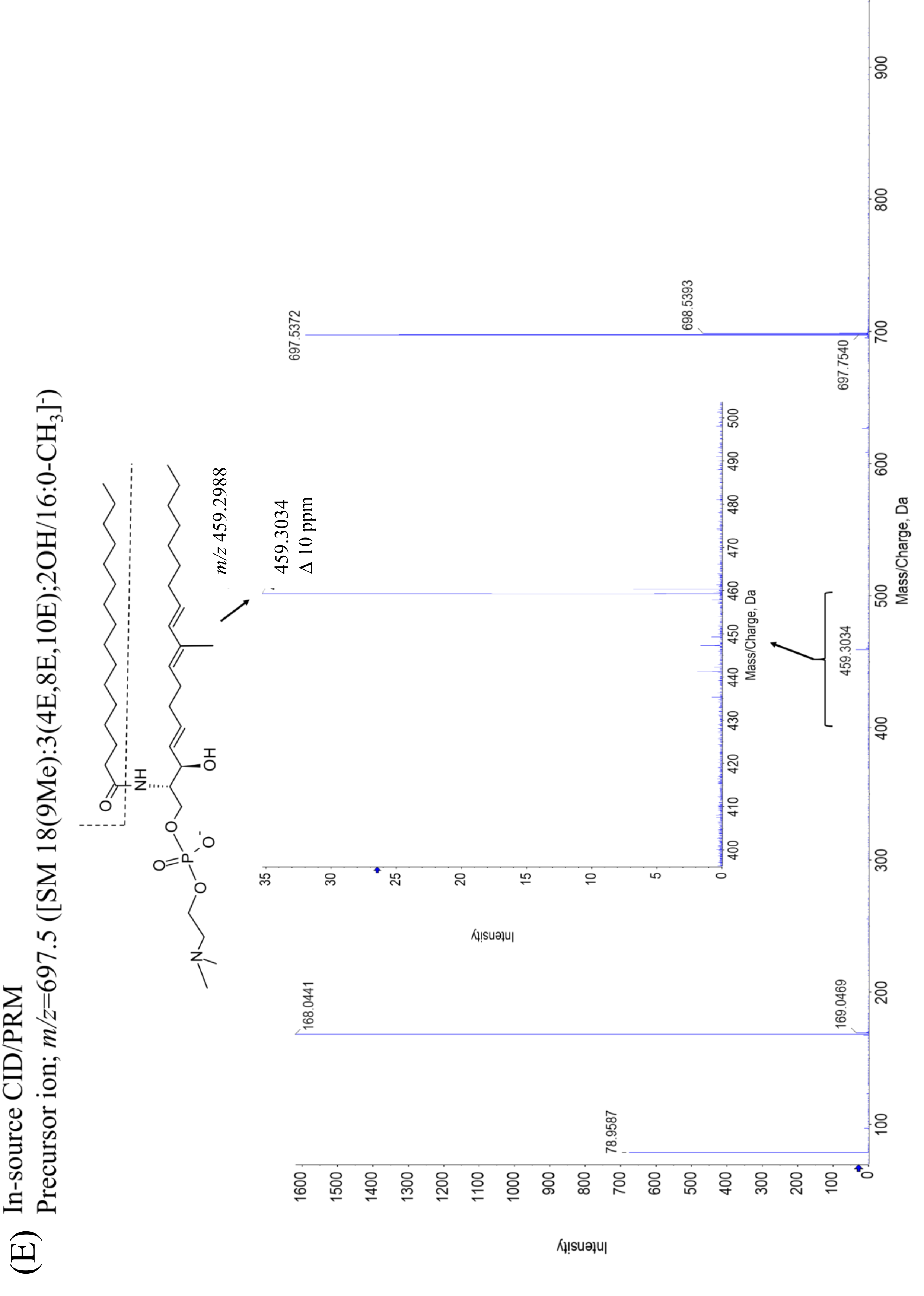

Figure S9. Continue.

(F) XICs of SMs with an LCB 18:2(4E,8Z);2OH

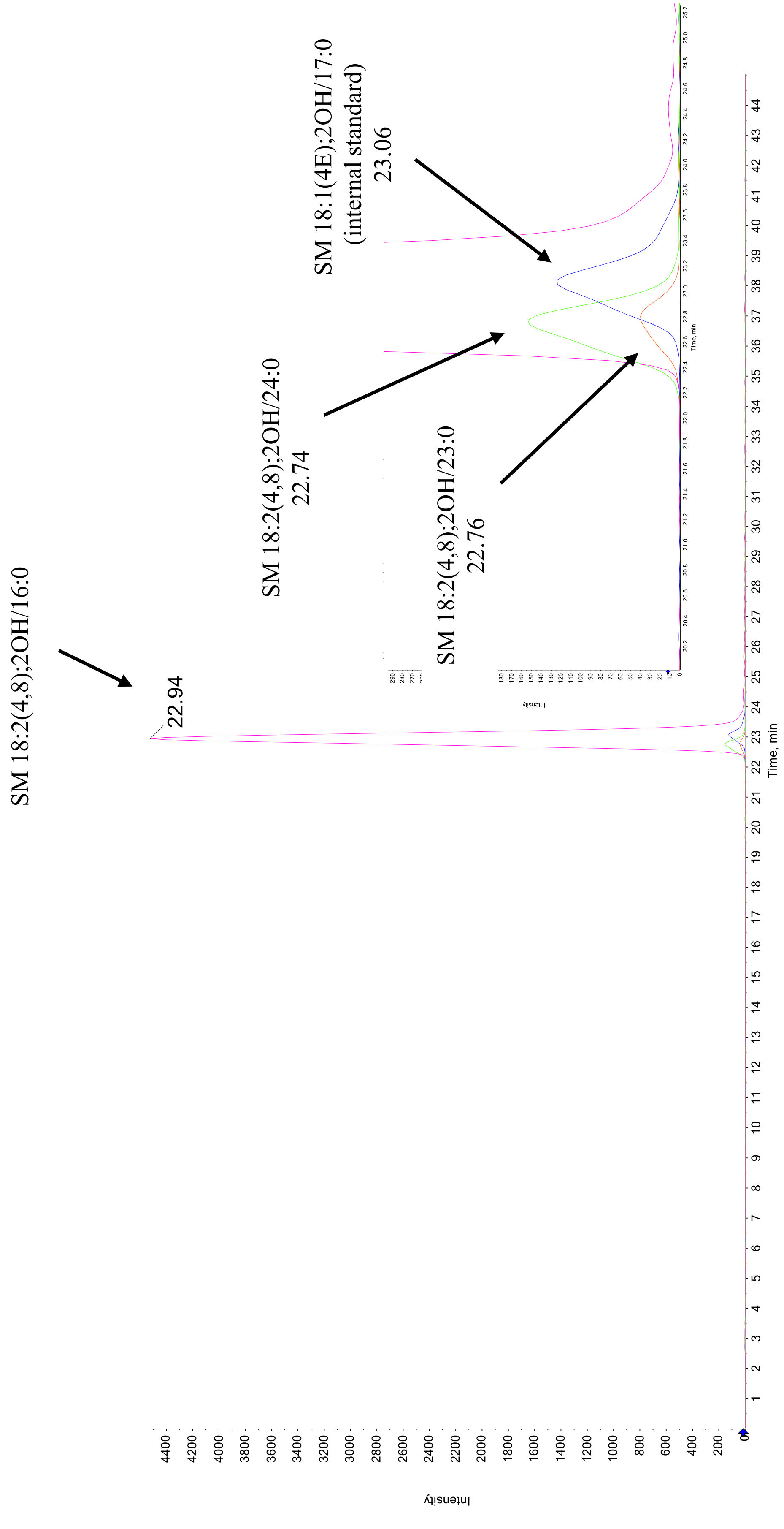

Figure S9. Continue.

(G) XICs of SMs with an LCB 18:2(4E,8E);2OH

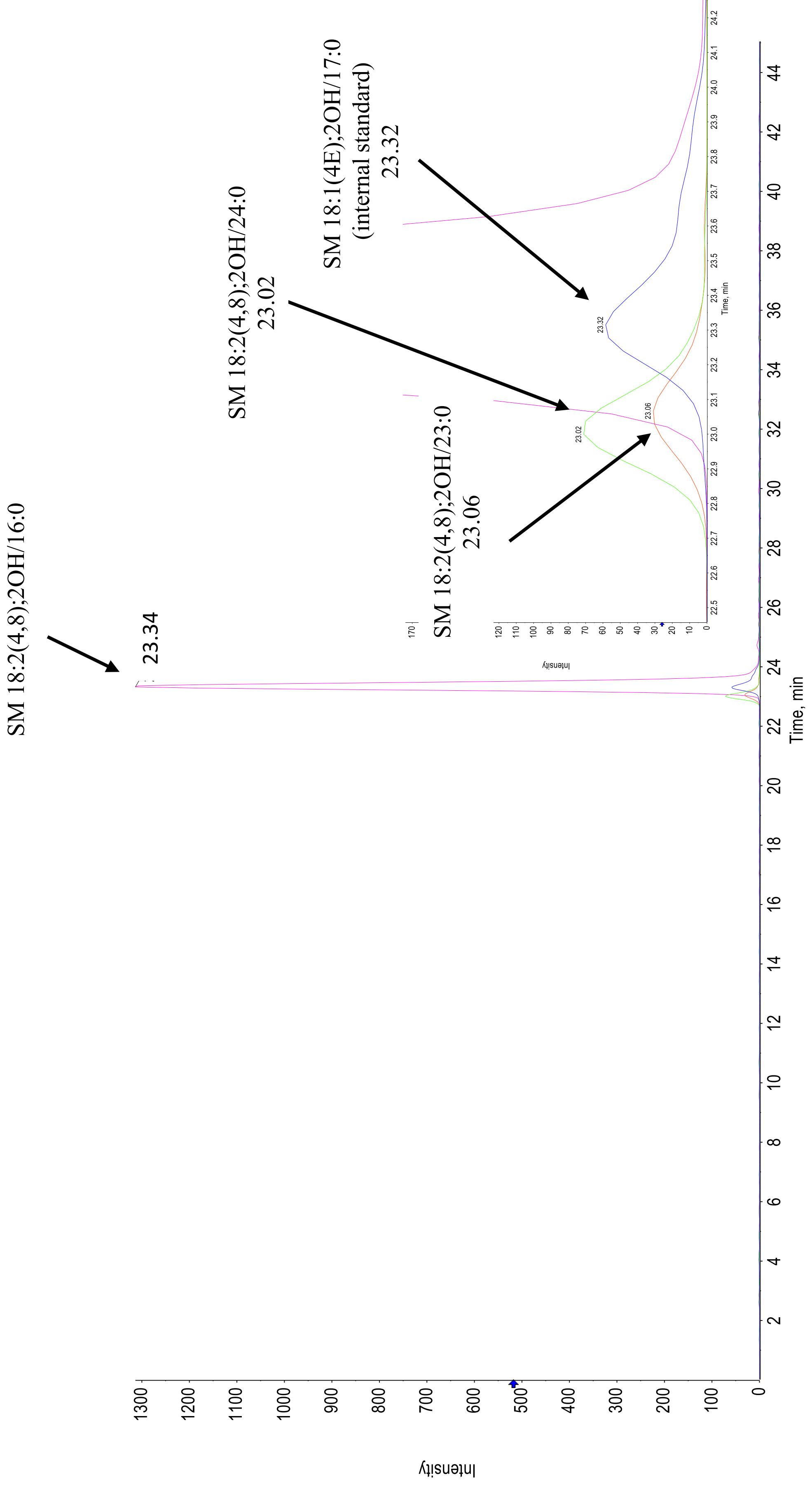

Figure S9. Continue.

(H) XICs of SMs with an LCB 18:3(4E,8E,10E);2OH

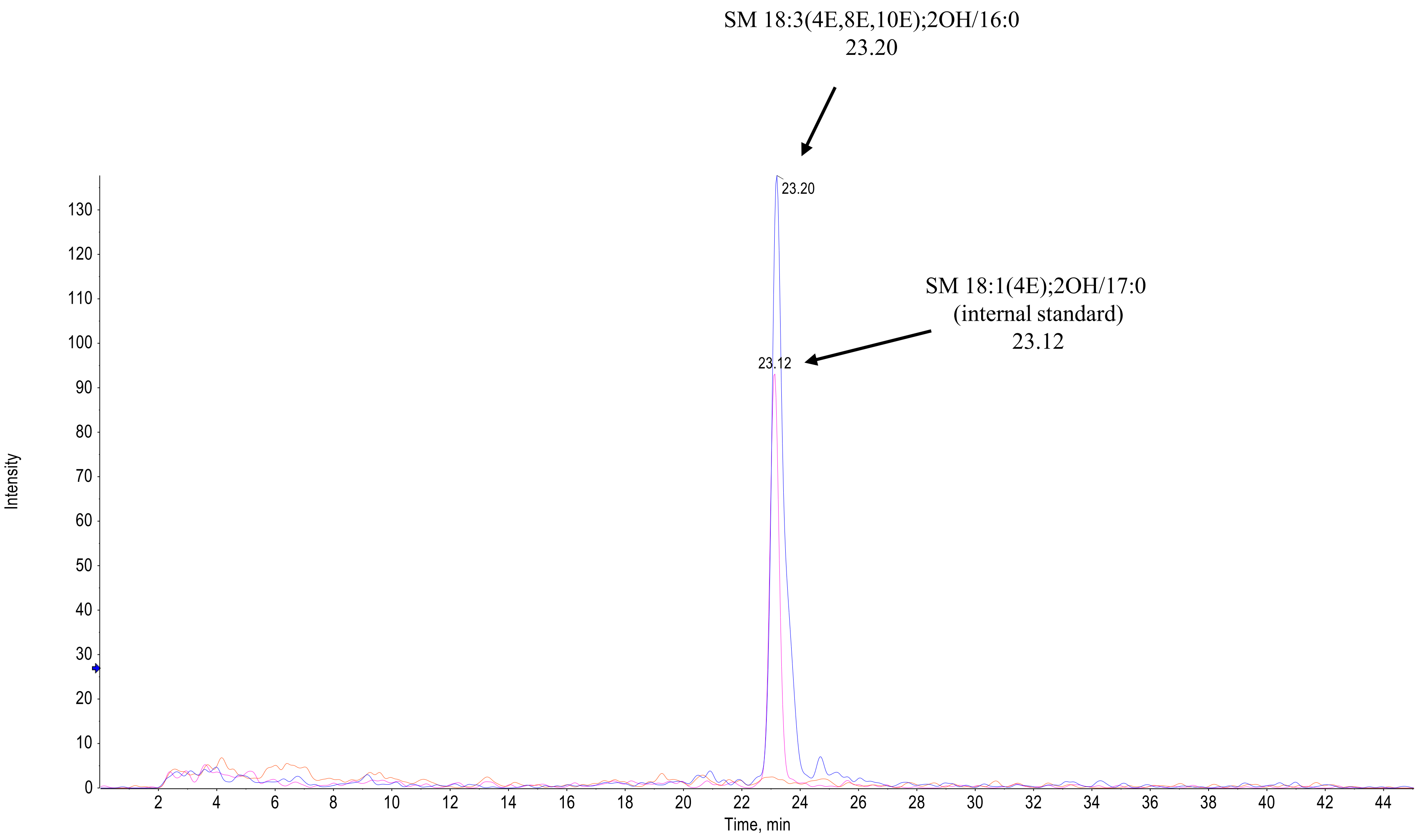

Figure S9. Continue.

(I) XICs of SMs with an LCB 18(9Me):2(4E,8E);2OH

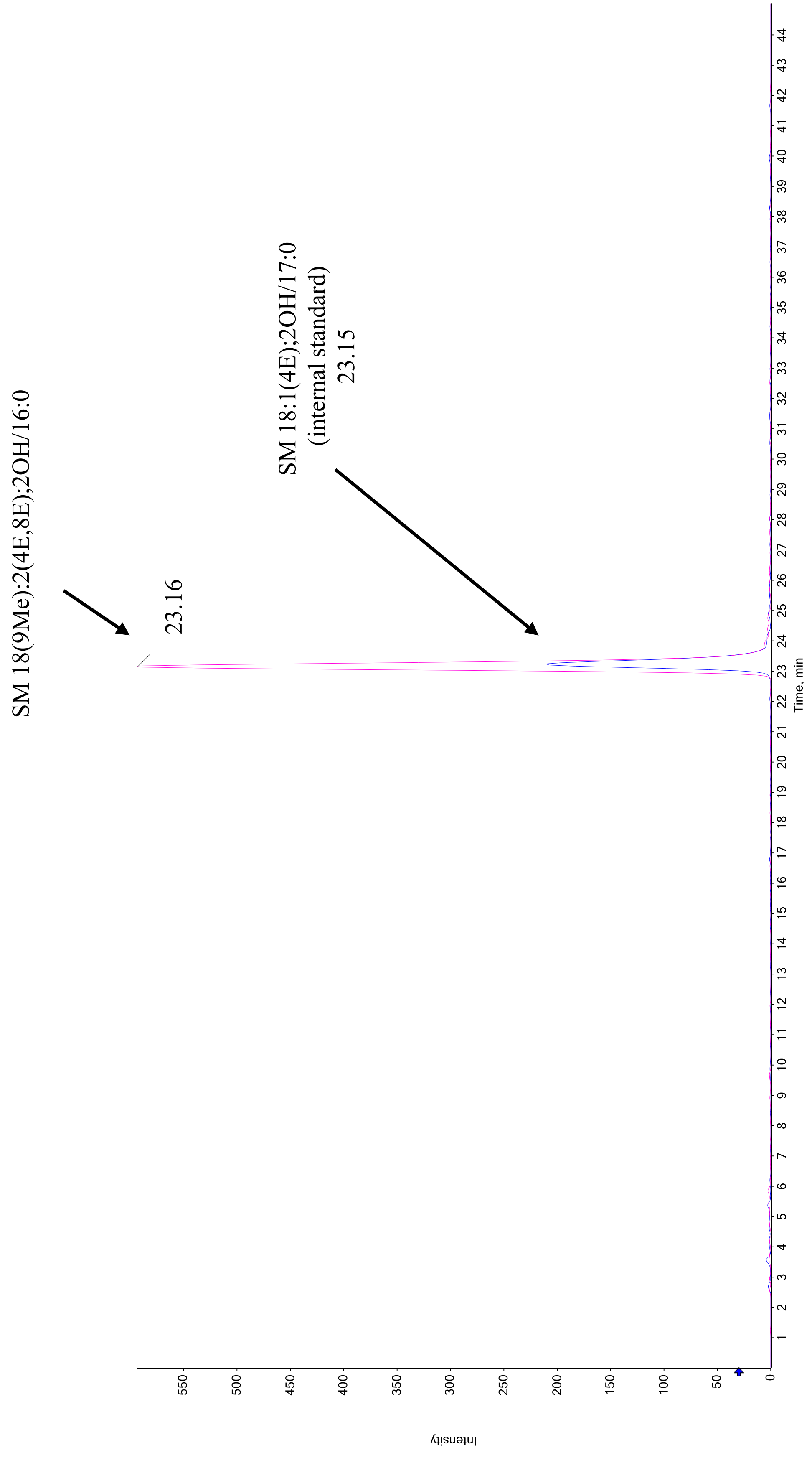

Figure S9. Continue.

(J) XICs of SMs with an LCB 18(9Me):3(4E,8E,10E);2OH

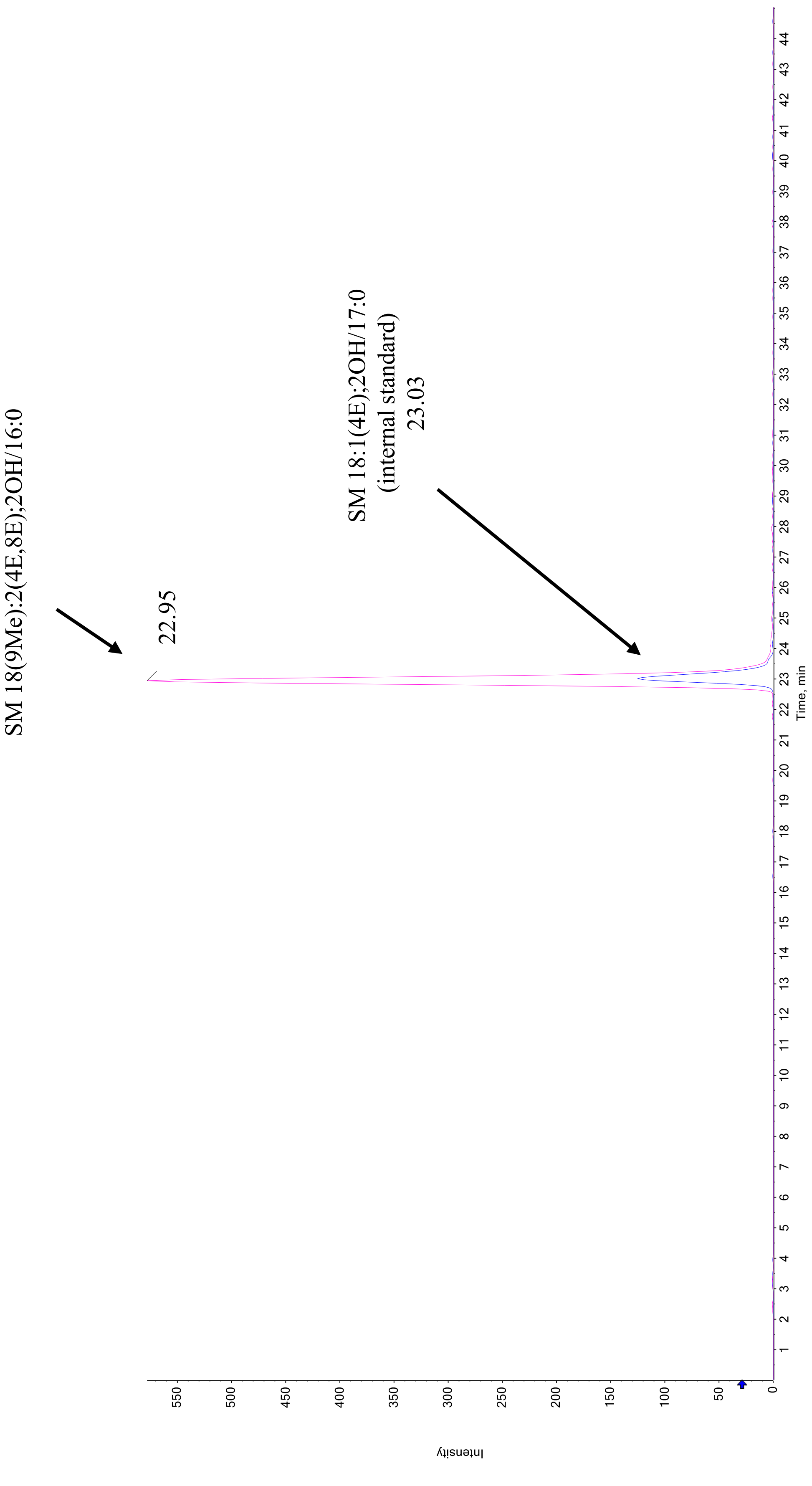

Figure S9: Typical product ion spectrum of demethylated SMs with an atypical LCB moieties and XICs of SMs based on insource CID/PRM on ESI-negative mode. (A) Product ion spectra of demethylated SM 18:2(4E,8Z);2OH/16:0. (B) Product ion spectra of demethylated SM 18:2(4E,8E);2OH/16:0. (C) Product ion spectra of demethylated SM 18(9Me):2(4E,8E);2OH/16:0. (D) Product ion spectra of demethylated SM 18:3(4E,8E,10E);2OH/16:0. (E) Product ion spectra of demethylated SM 18(9Me):3(4E,8E,10E);2OH/16:0.
(F) XIC of SM with an LCB 18:2(4E,8Z);2OH. (G) XIC of SM with an LCB 18:2(4E,8Z);2OH.
(H) XIC of SM with an LCB 18:3(4E,8E,10E);2OH. (I) XIC of SM with an LCB 18(9Me):2(4E,8E);2OH. (J) XIC of SM with an LCB 18(9Me):3(4E,8E,10E);2OH.
XICs were drawn based on the product ion $[SM\text{-}CH_3\text{-fatty acyl}]^-$ indicated in A-E.

Figure S10.

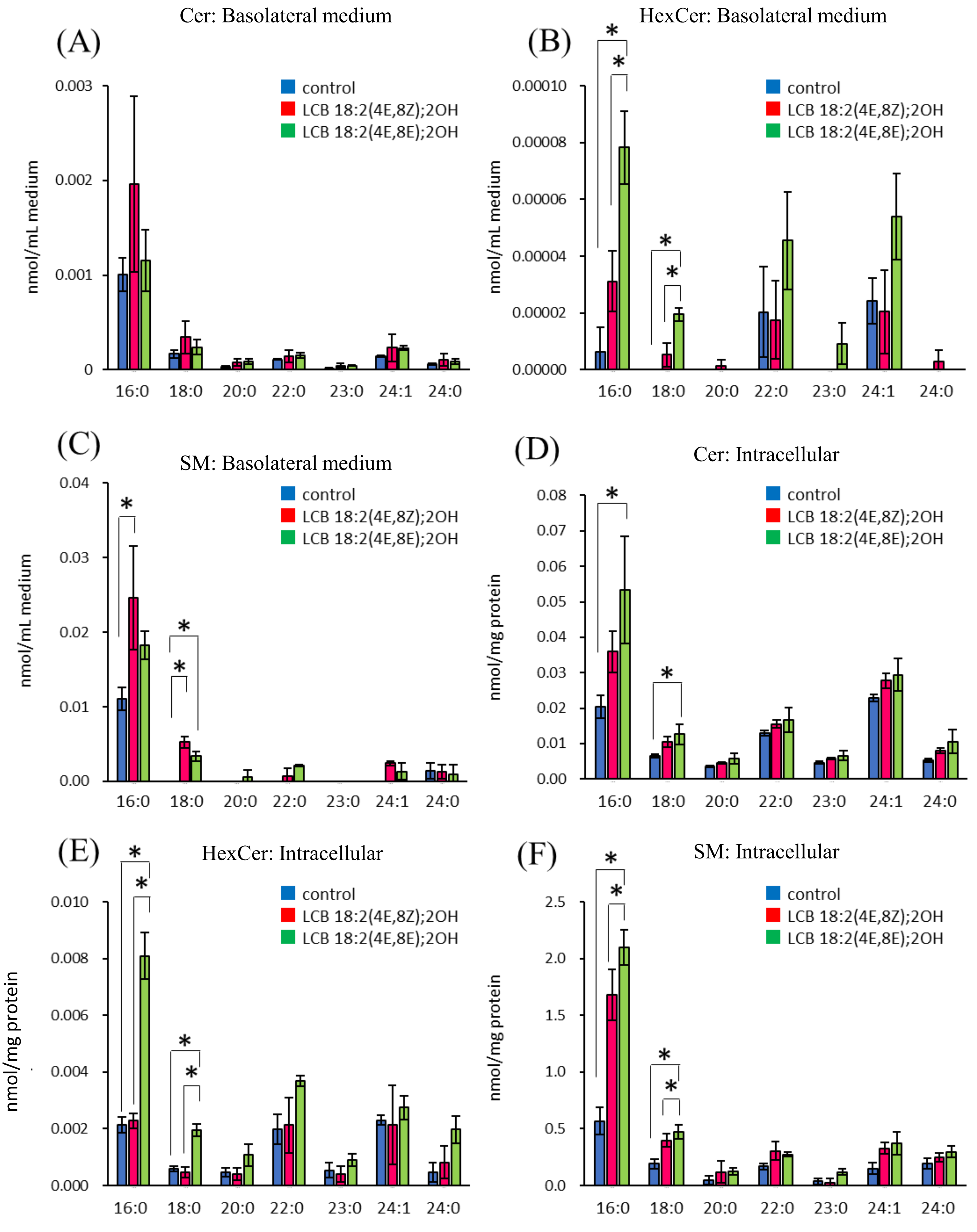

(A)
Cer: Basolateral medium
nmol/mL medium
0.003
0.002
0.001
0
control
LCB 18:2(4E,8Z);2OH
LCB 18:2(4E,8E);2OH
16:0
18:0
20:0
22:0
23:0
24:1
24:0
(B)
HexCer: Basolateral medium
nmol/mL medium
0.00010
0.00008
0.00006
0.00004
0.00002
0.00000
(C)
SM: Basolateral medium
nmol/mL medium
0.04
0.03
0.02
0.01
0.00
(D)
Cer: Intracellular
nmol/mg protein
0.08
0.07
0.06
0.05
0.04
0.03
0.02
0.01
0.00
(E)
HexCer: Intracellular
nmol/mg protein
0.010
0.008
0.006
0.004
0.002
0.000
(F)
SM: Intracellular
nmol/mg protein
2.5
2.0
1.5
1.0
0.5
0.0

Figure S10: Molecular species in the basolateral medium and intracellular sphingolipids with an LCB 18:2(4E,8Z);2OH moiety or LCB 18:2(4E,8E);2OH moiety in Caco-2 cells treated with LCB 18:2(4E,8Z);2OH or LCB 18:2(4E,8E);2OH. for 24 h. (A) Ceramides in the basolateral medium. (B) Hexosylceramides in the basolateral medium. (C) Sphingomyelins in the basolateral medium. (D) Ceramides in cells. (E) Hexosylceramides in cells. (F) Sphingomyelins in cells. Lipid amounts of basolateral media and cells were normalized with medium volumes and cell protein amounts, respectively. Values are presented as mean ± standard deviation (SD) (n = 3; *significant difference by Tukey's multiple comparison test, $P < 0.05$).

Figure S.11

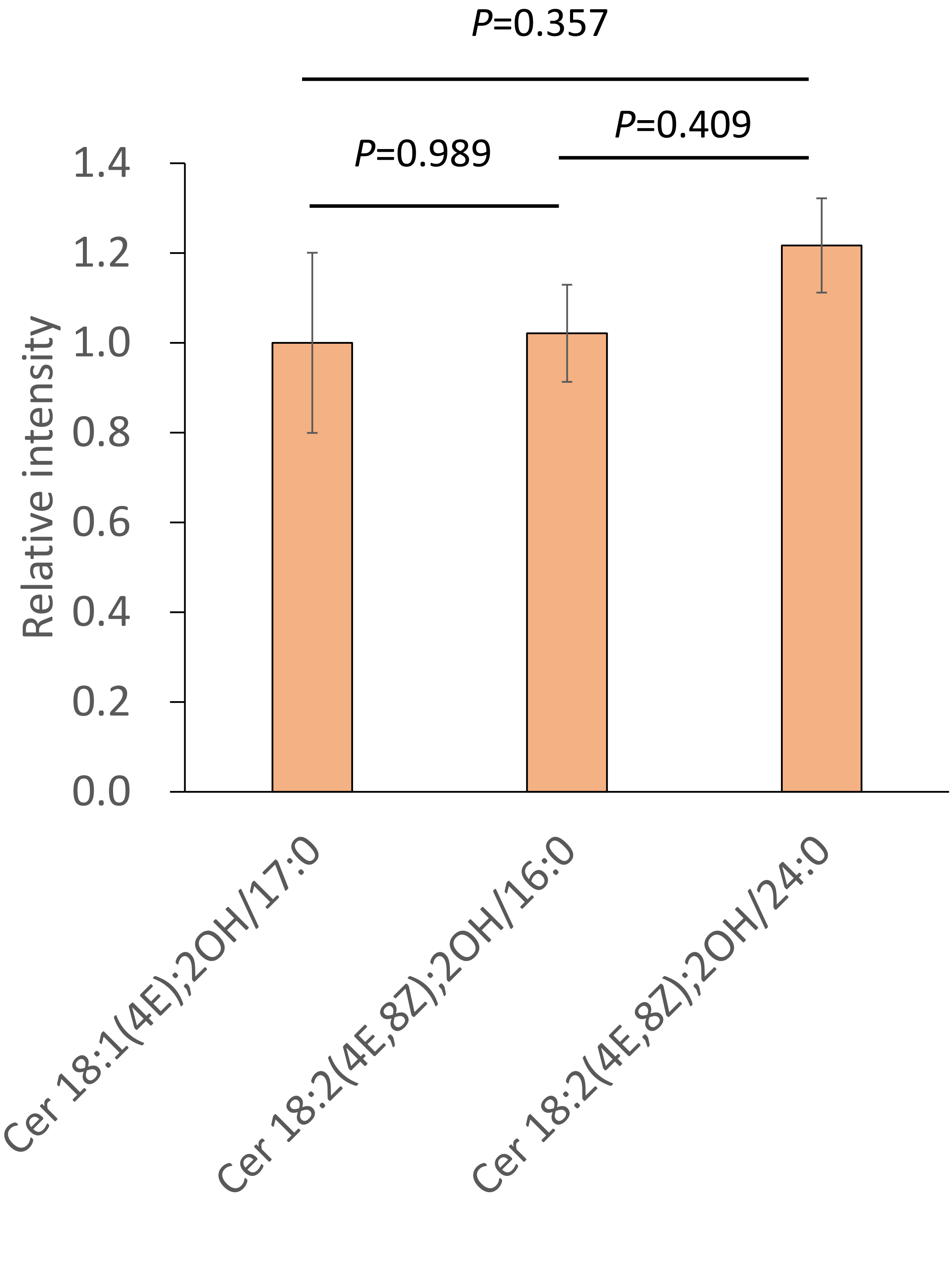

Figure S11. Comparison of the detection intensity of Cer 18:1(4E);2OH/17:0, Cer 18:2(4E,8Z);2OH/16:0 and Cer 18:2(4E,8Z);2OH/24:0. Three Cers were dissolved with acetonitrile/methanol=19:1 (v/v/) at final concentration of 0.25 μM and 10 μL of Cers solutions were injected onto LC-MS/MS. The experiment was repeated three times. Statistical significances were analyzed by one-way ANOVA folled by Tukey's multiple comparison test as post hoc test.

Figure S.12

(A) Preparative HPLC chromatogram of crude Cer 18:2(4E,8Z);2OH/16:0

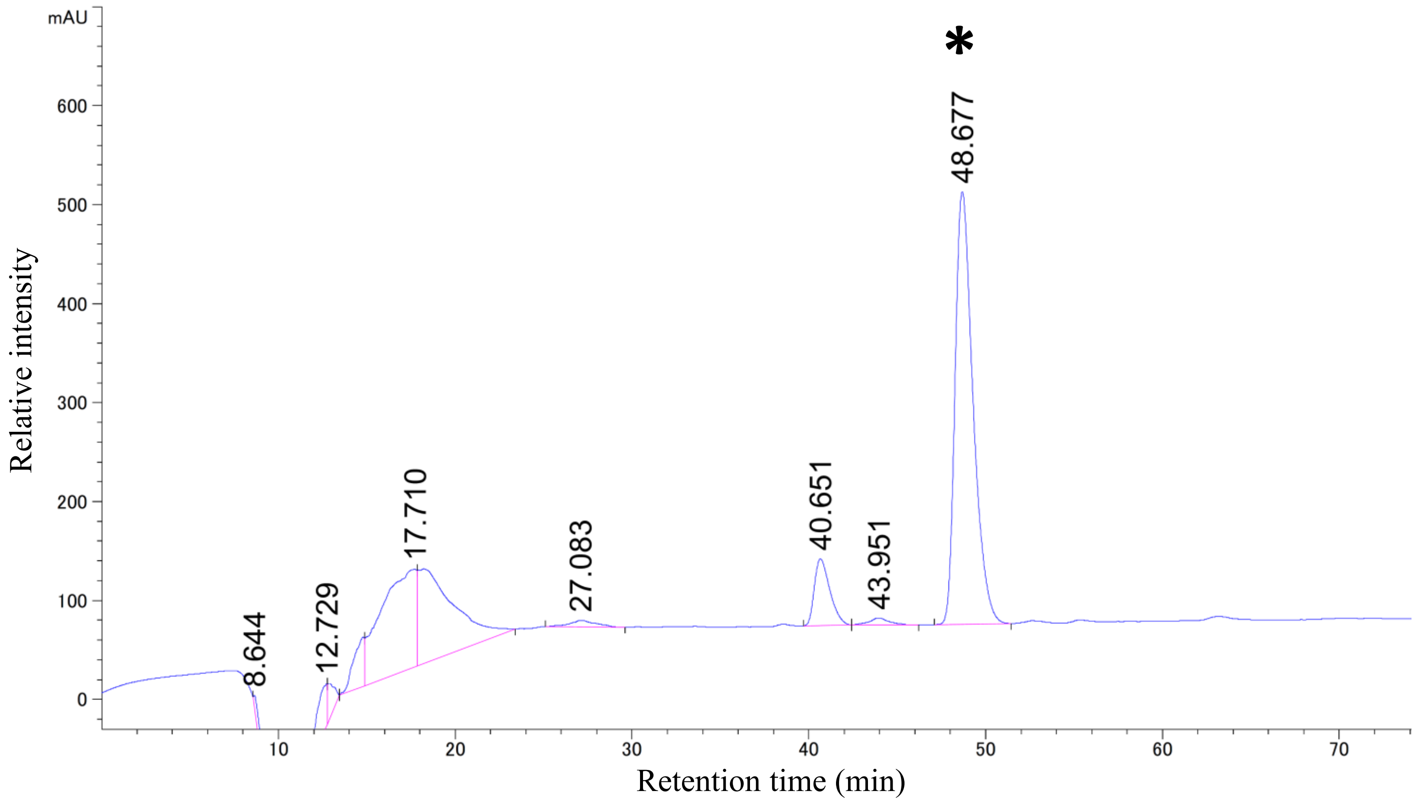

(B) Analytical HPLC chromatogram of blank sample (solvent)

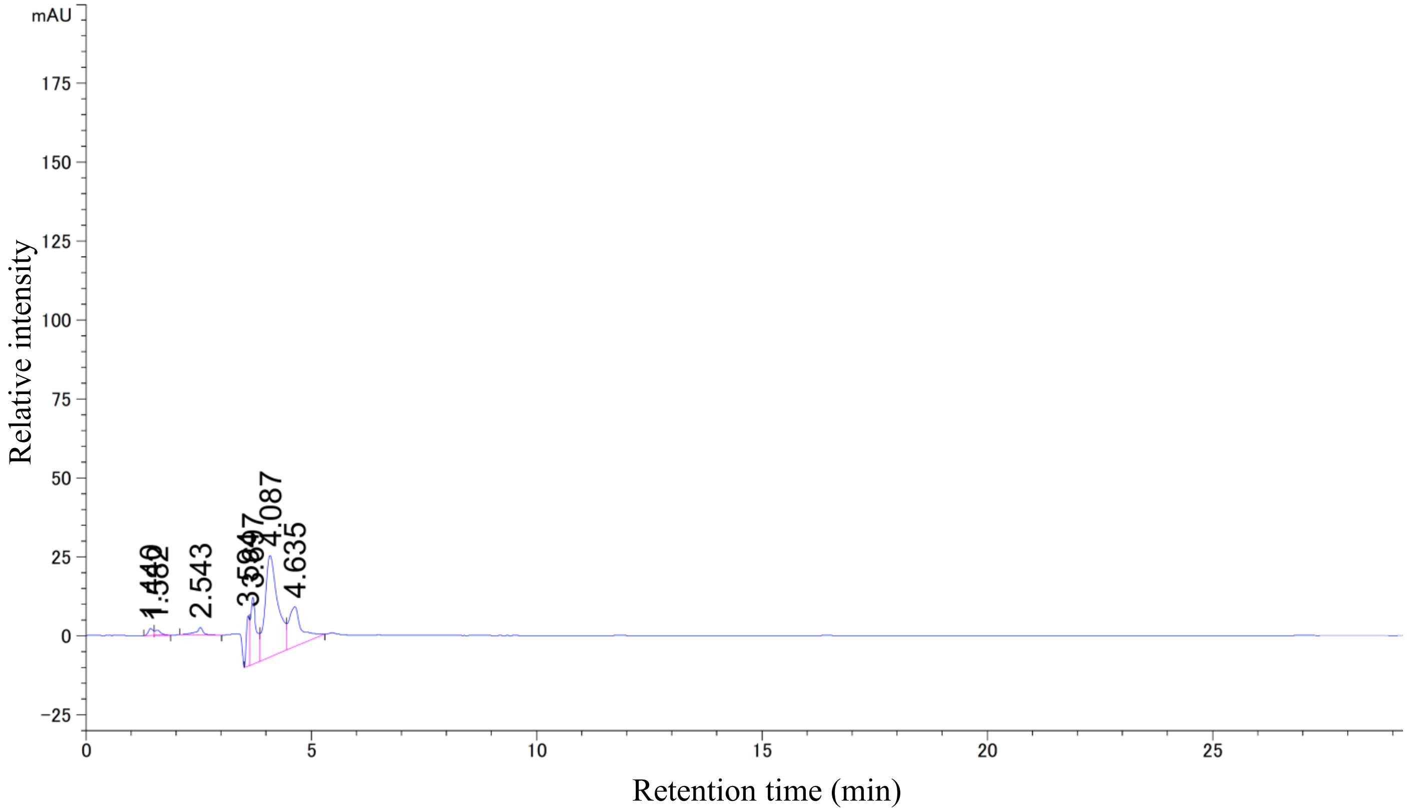

Figure S.12. Continue.

(C) Analytical HPLC chromatogram of purified Cer 18:2(4E,8Z);2OH/16:0

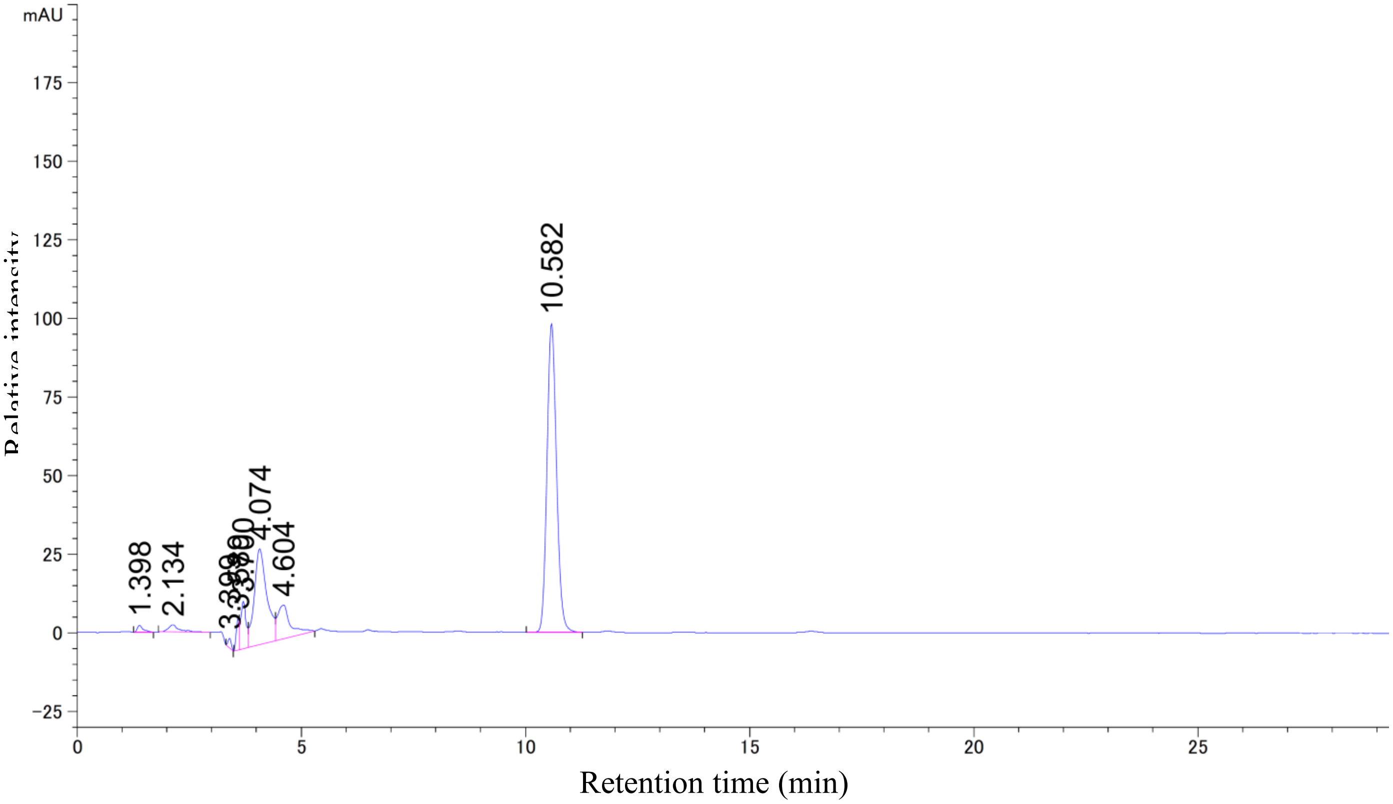

Figure S12: Chromatograms of preparative and analytical HPLC for Cer 18:2(4E,8Z);2OH/16:0. (A) Chromatogram of preparative HPLC. The peak indicated by the asterisks was collected and confirmed to Cer 18:2(4E,8Z);2OH/16:0 by ESI-MS/MS analysis. (B) Analytical HPLC chromatogram of blank sample (solvent). (C) Analytical HPLC chromatogram of purified Cer 18:2(4E,8Z);2OH/16:0.

Figure S.13

(A) Preparative HPLC chromatogram of crude Cer 18:2(4E,8Z);2OH/24:0

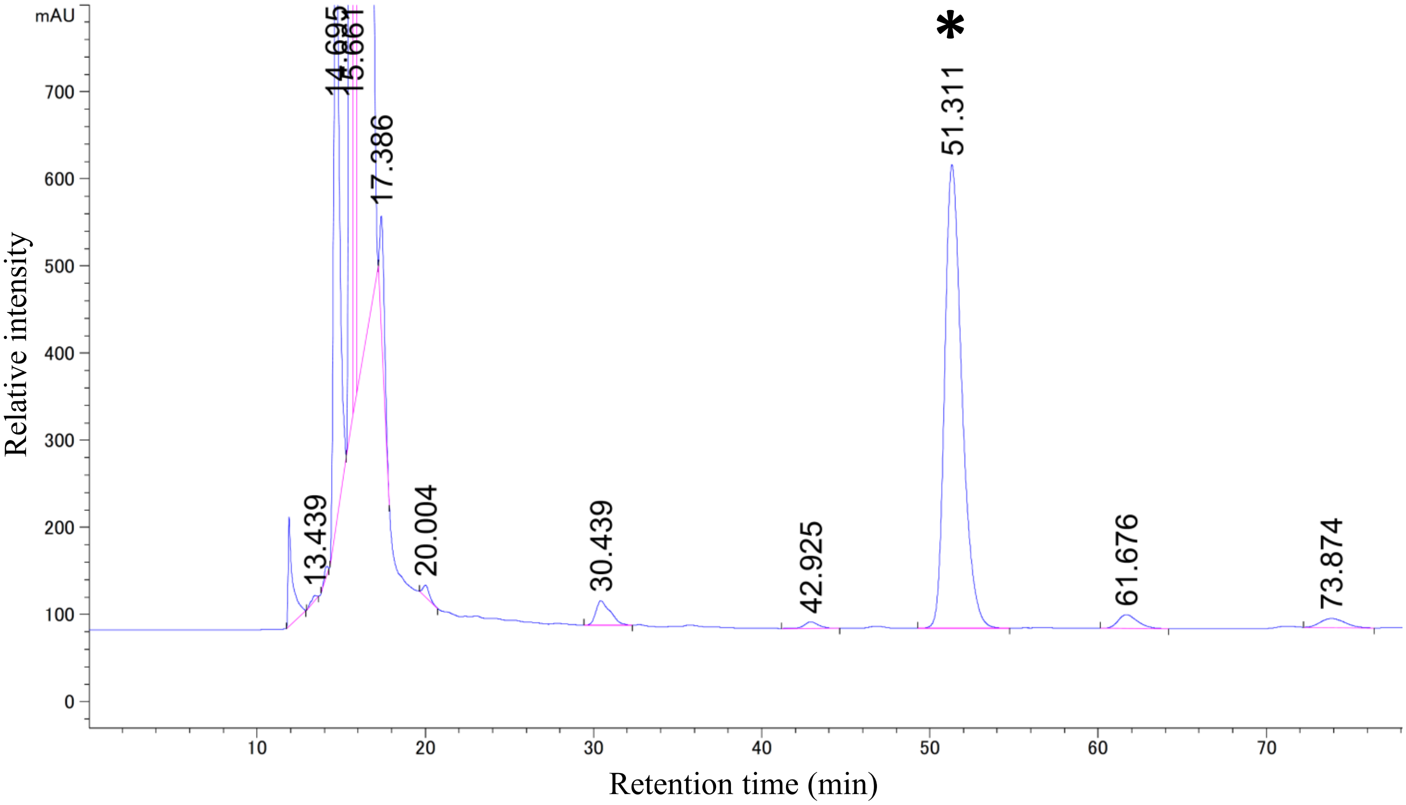

(B) Analytical HPLC chromatogram of Blank sample (solvent)

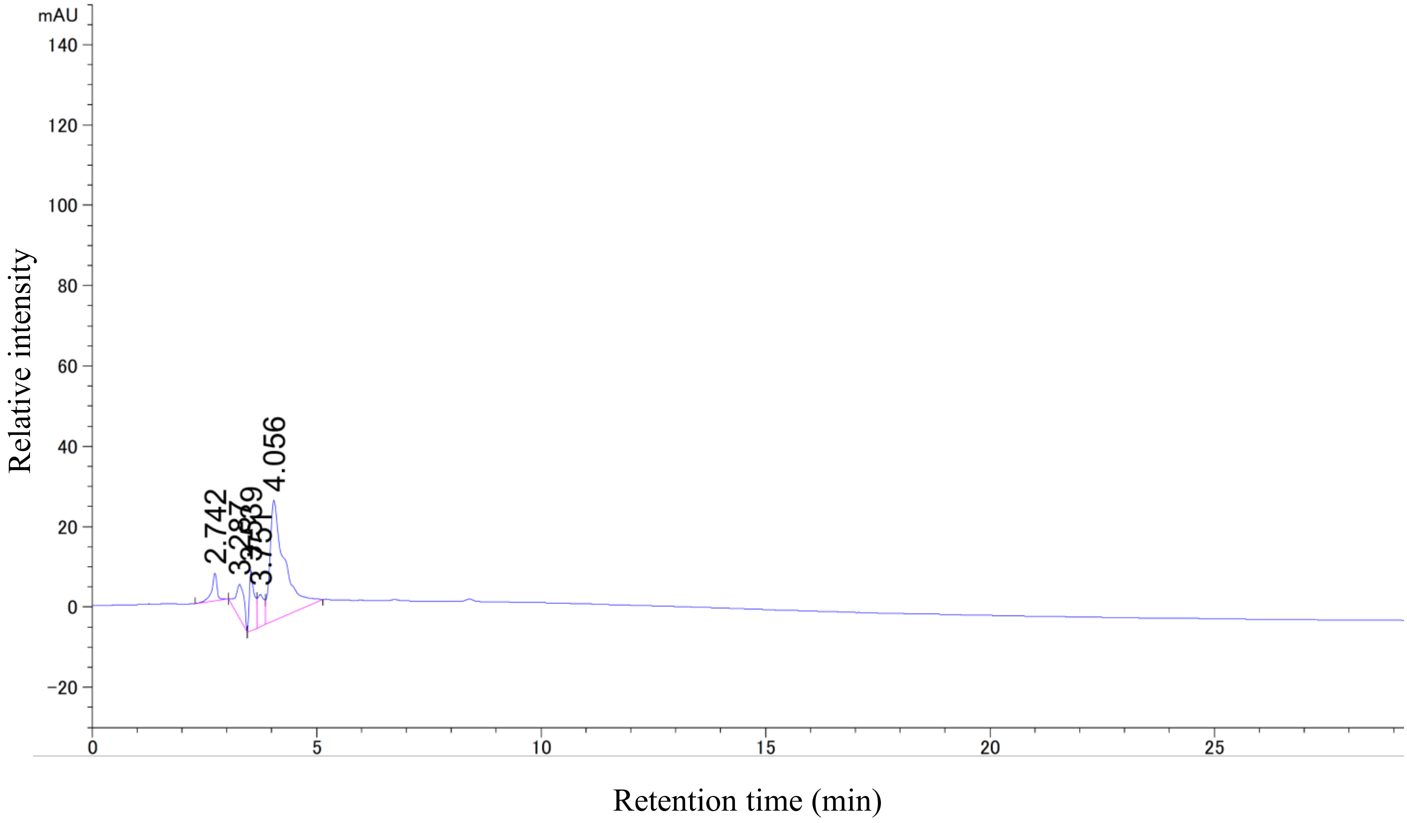

Figure S.13. Continue.

(C) Analytical HPLC chromatogram of purified Cer 18:2(4E,8Z);2OH/24:0

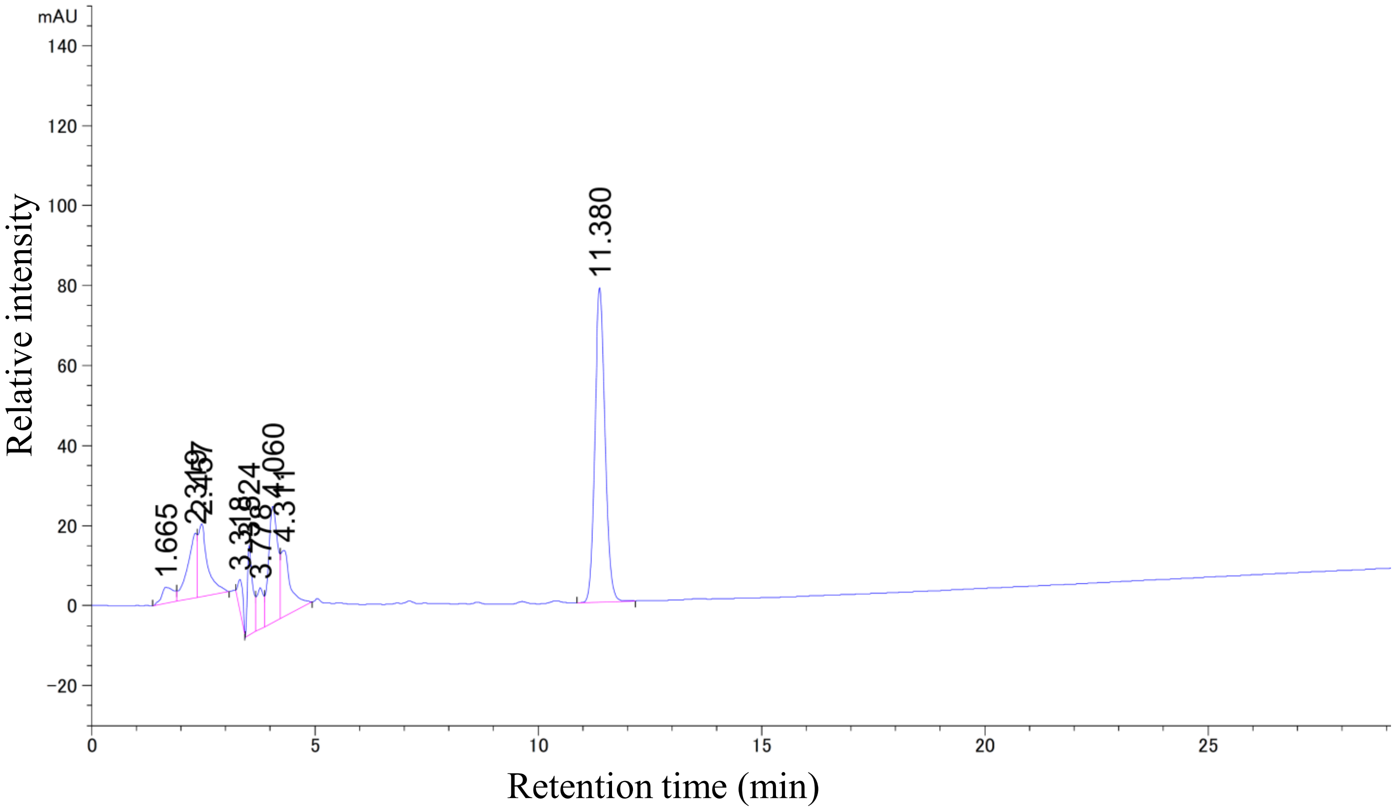

Figure S13: Chromatograms of preparative and analytical HPLC for Cer 18:2(4E,8Z);2OH/24:0. (A) Chromatogram of preparative HPLC. The peak indicated by the asterisk was collected and confirmed to Cer 18:2(4E,8Z);2OH/24:0 by ESI-MS/MS analysis. (B) Analytical HPLC chromatogram of blank sample (solvent). (C) Analytical HPLC chromatogram of purified Cer 18:2(4E,8Z);2OH/24:0.

Figure S.14

(A) Blank sample (solvent)

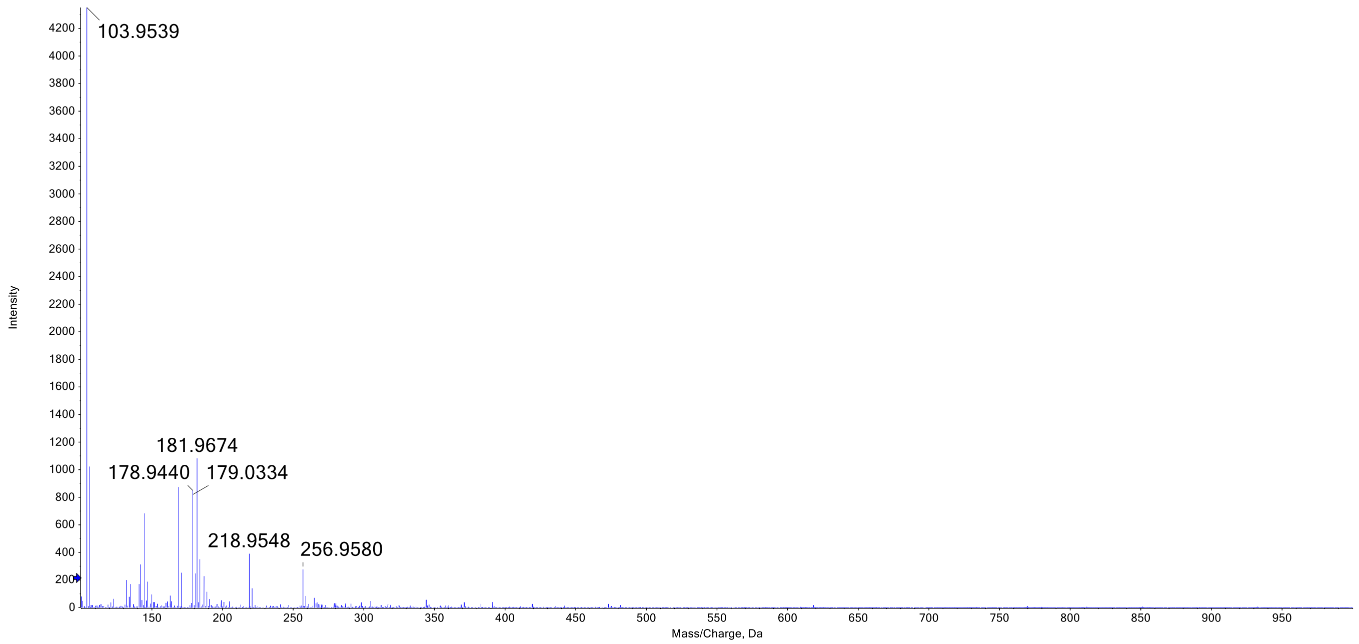

Figure S.14. Continue.

(B) TOF MS spectrum of Cer 18:2(4E,8Z);2OH/16:0

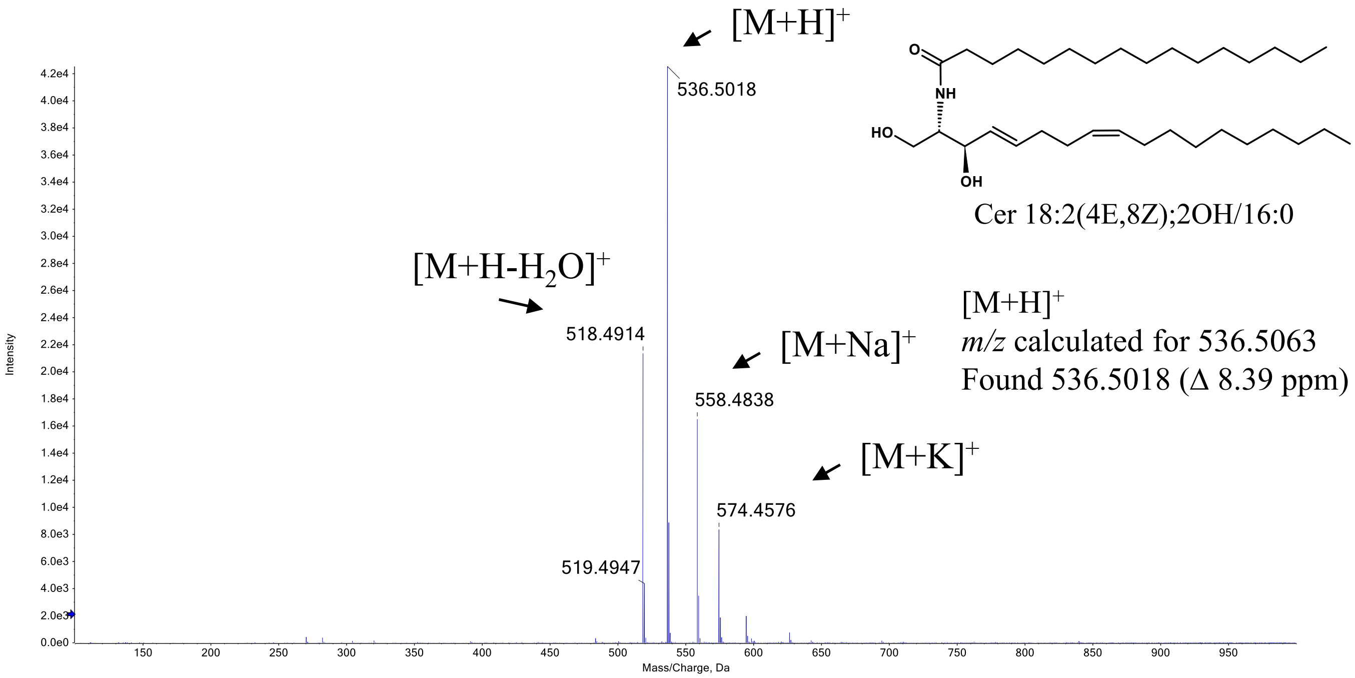

(C) Product ion spectrum of Cer 18:2(4E,8Z);2OH/16:0

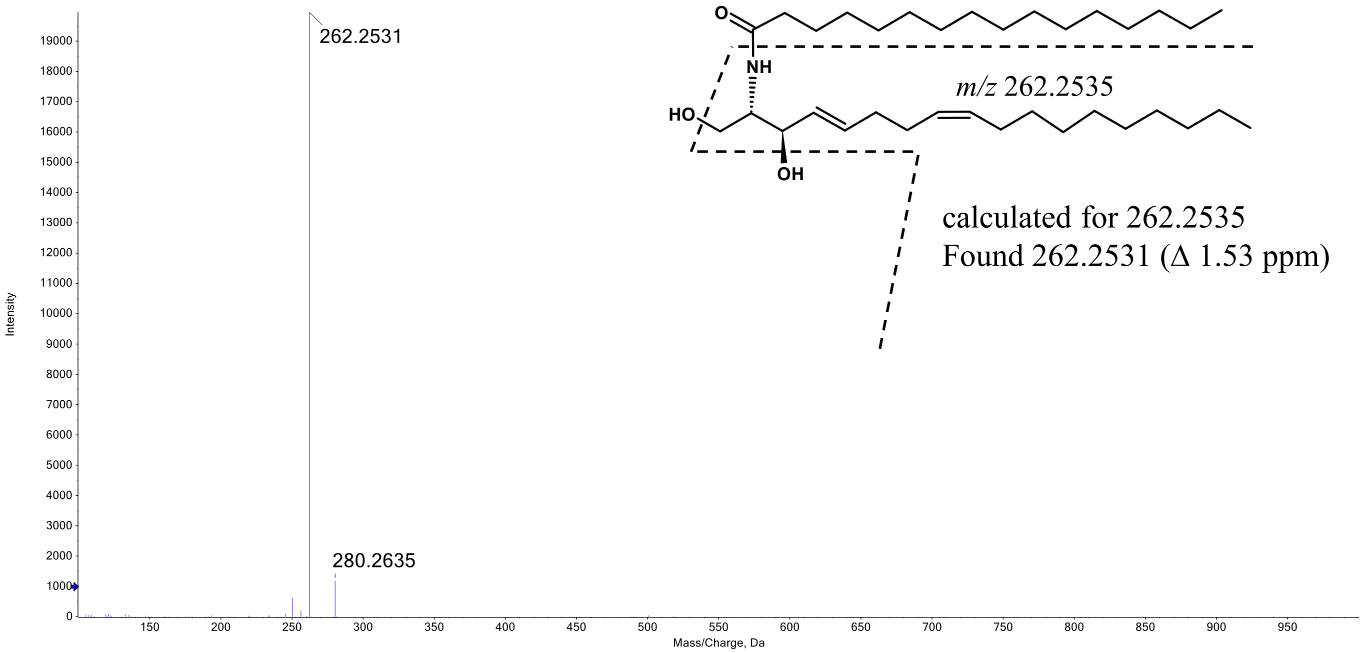

Figure S.14. Continue.

(D) TOF MS spectrum of Cer 18:2(4E,8Z);2OH/24:0

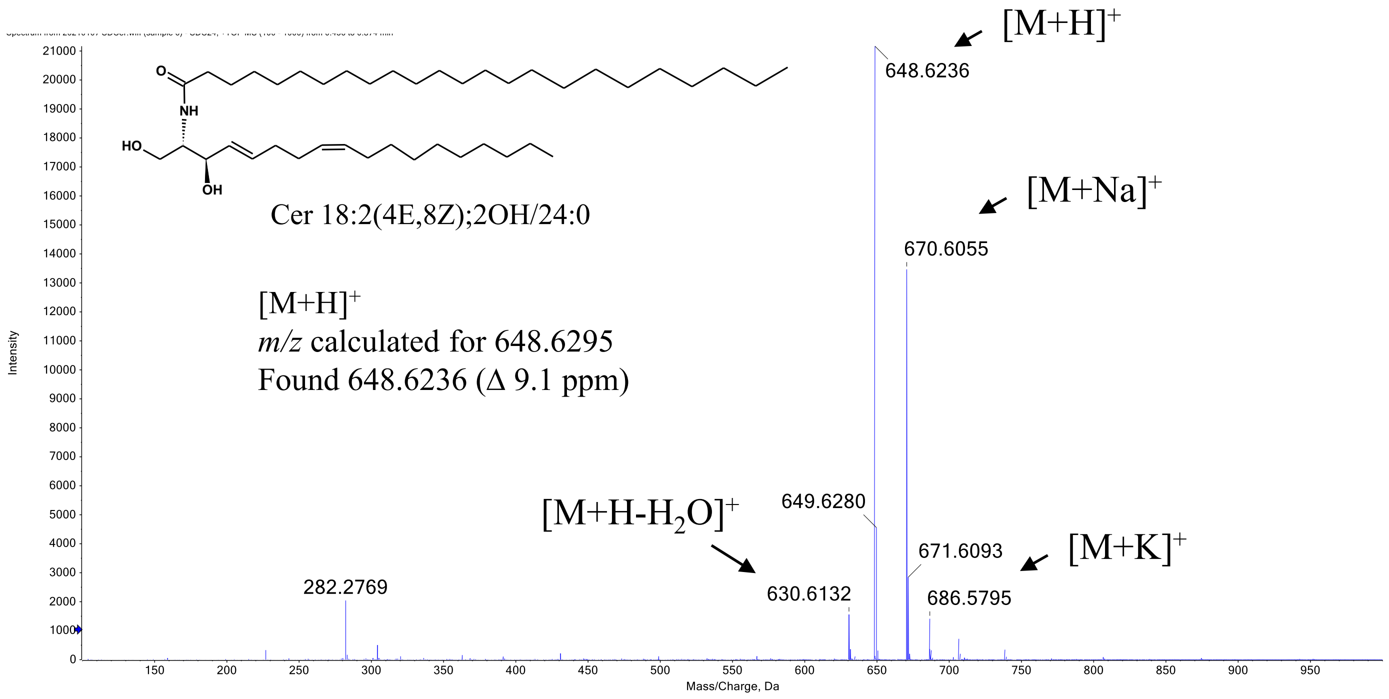

(E) Product ion spectrum of Cer 18:2(4E,8Z);2OH/24:0

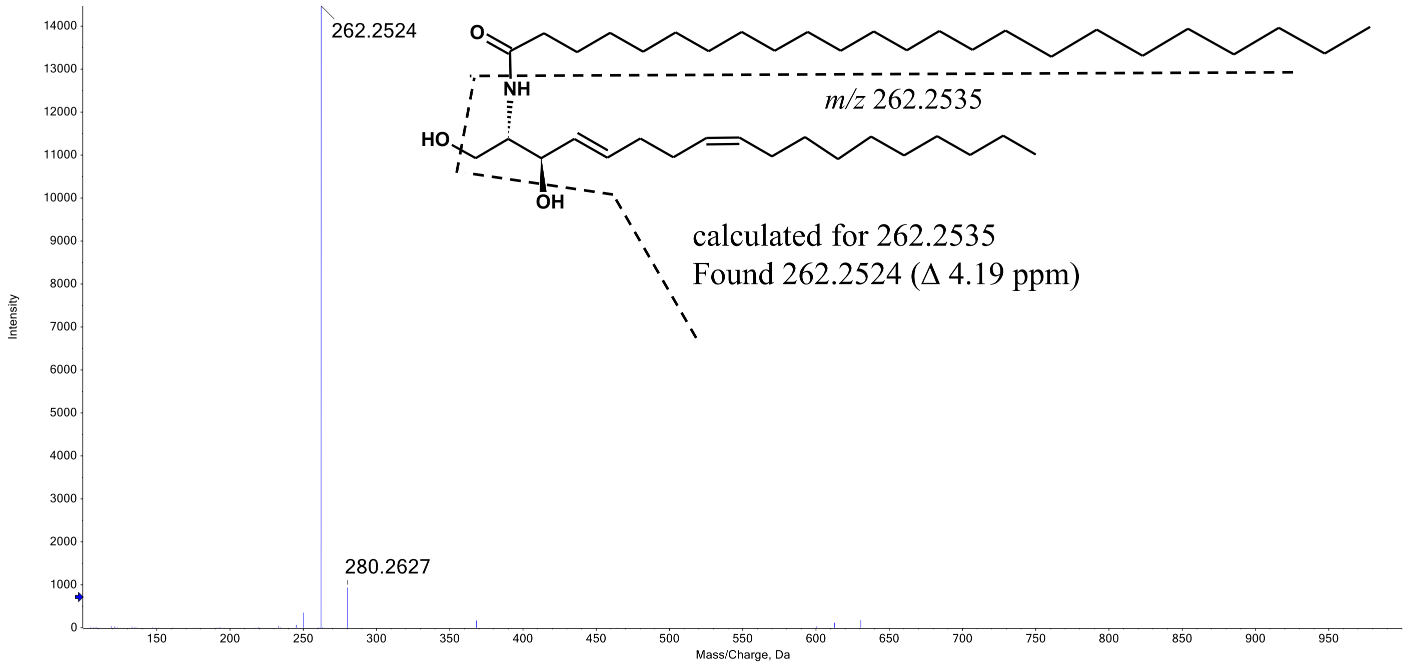

Figure S14: ESI-MS spectrum of synthetic Cer 18:2(4E,8Z);2OH/16:0 and Cer 18:2(4E,8Z);2OH/24:0. (A) TOF MS spectrum of Cer 18:2(4E,8Z);2OH/16:0. (B) product ion spectrum of Cer 18:2(4E,8Z);2OH/16:0. (C) TOF MS spectrum of Cer 18:2(4E,8Z);2OH/24:0. (D) Product ion spectrum of Cer 18:2(4E,8Z);2OH/24:0.

Figure S15.

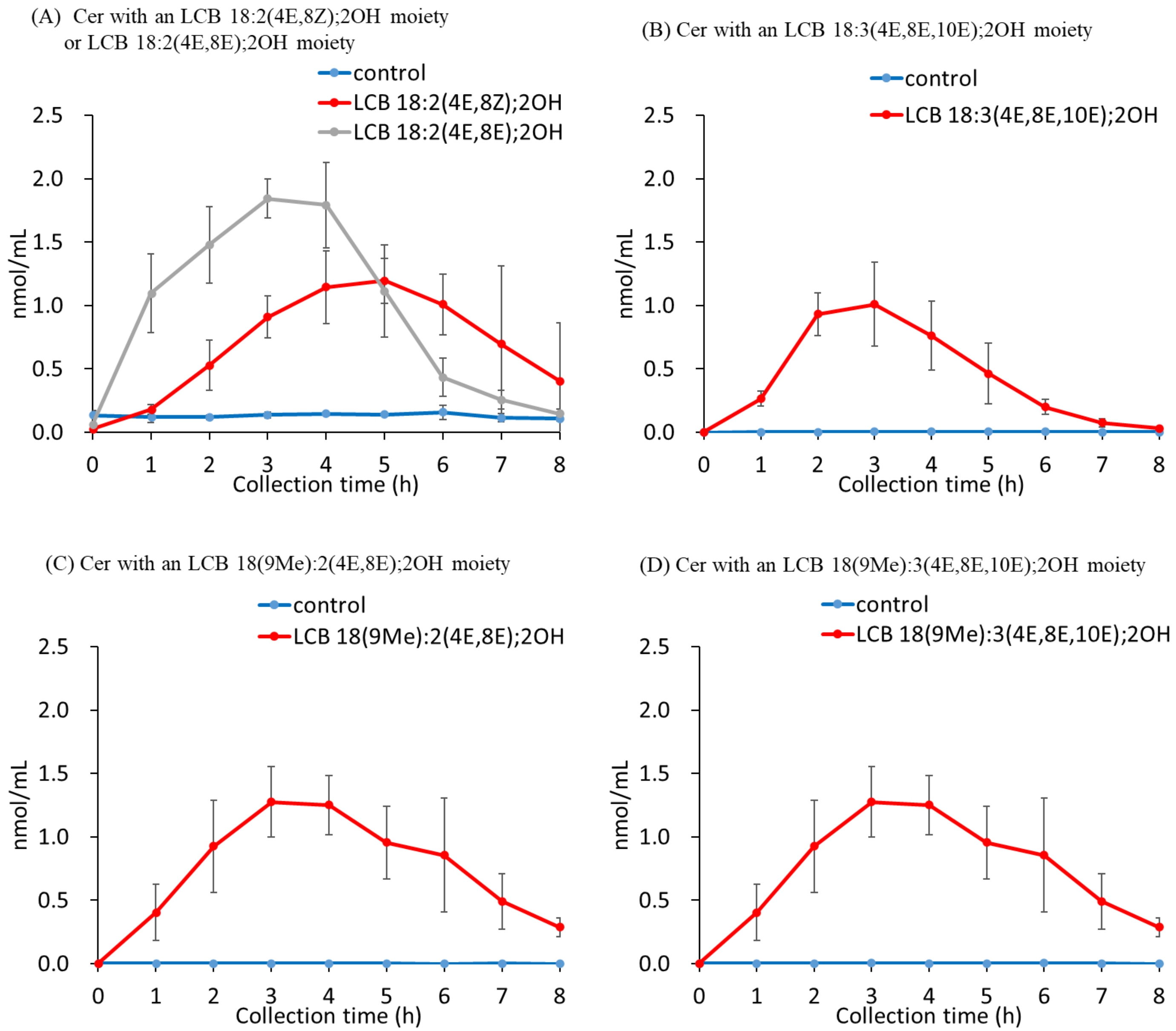

Figure S15: Molar concentrations of ceramides in chyle of rats after enteral administration of long-chain base emulsions. (A) Cers with an LCB 18:2(4E,8Z);2OH moiety or an LCB 18:2(4E,8E);2OH moiety, (B) Cers with an LCB 18:3(4E,8E,10E);2OH, (C) Cers with an LCB 18(9Me):2(4E,8E);2OH moiety, and (D) Cers with an LCB 18(9Me):3(4E,8E,10E);2OH moiety in the lymph of rats following enteral administration of long-chain base emulsions. Lipid amounts were normalized with collected chyle volumes. Values are presented as mean ± standard deviation.

Figure S16.

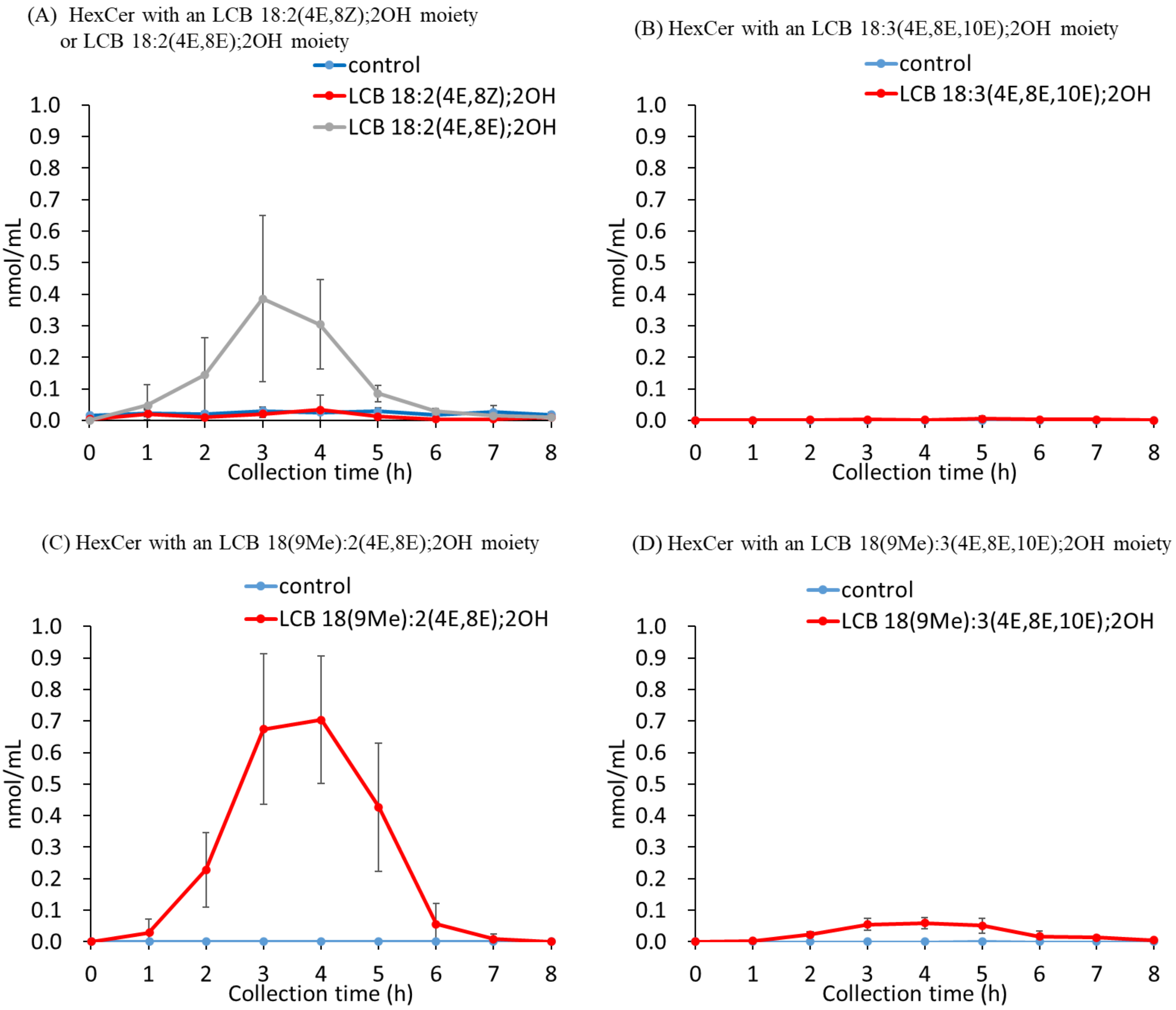

Figure S16: Molar concentrations of hexosylceramides in chyle of rats after enteral administration of long-chain base emulsions. (A) HexCers with an LCB 18:2(4E,8Z);2OH moiety or an LCB 18:2(4E,8E);2OH moiety, (B) HexCers with an LCB 18:3(4E,8E,10E);2OH, (C) HexCers with an LCB 18(9Me):2(4E,8E);2OH moiety, and (D) HexCers with an LCB 18(9Me):3(4E,8E,10E);2OH moiety in the lymph of rats following enteral administration of long-chain base emulsions. Lipid amounts were normalized with collected chyle volumes. Values are presented as mean ± standard deviation (SD) (n = 4–5).

Figure S17.

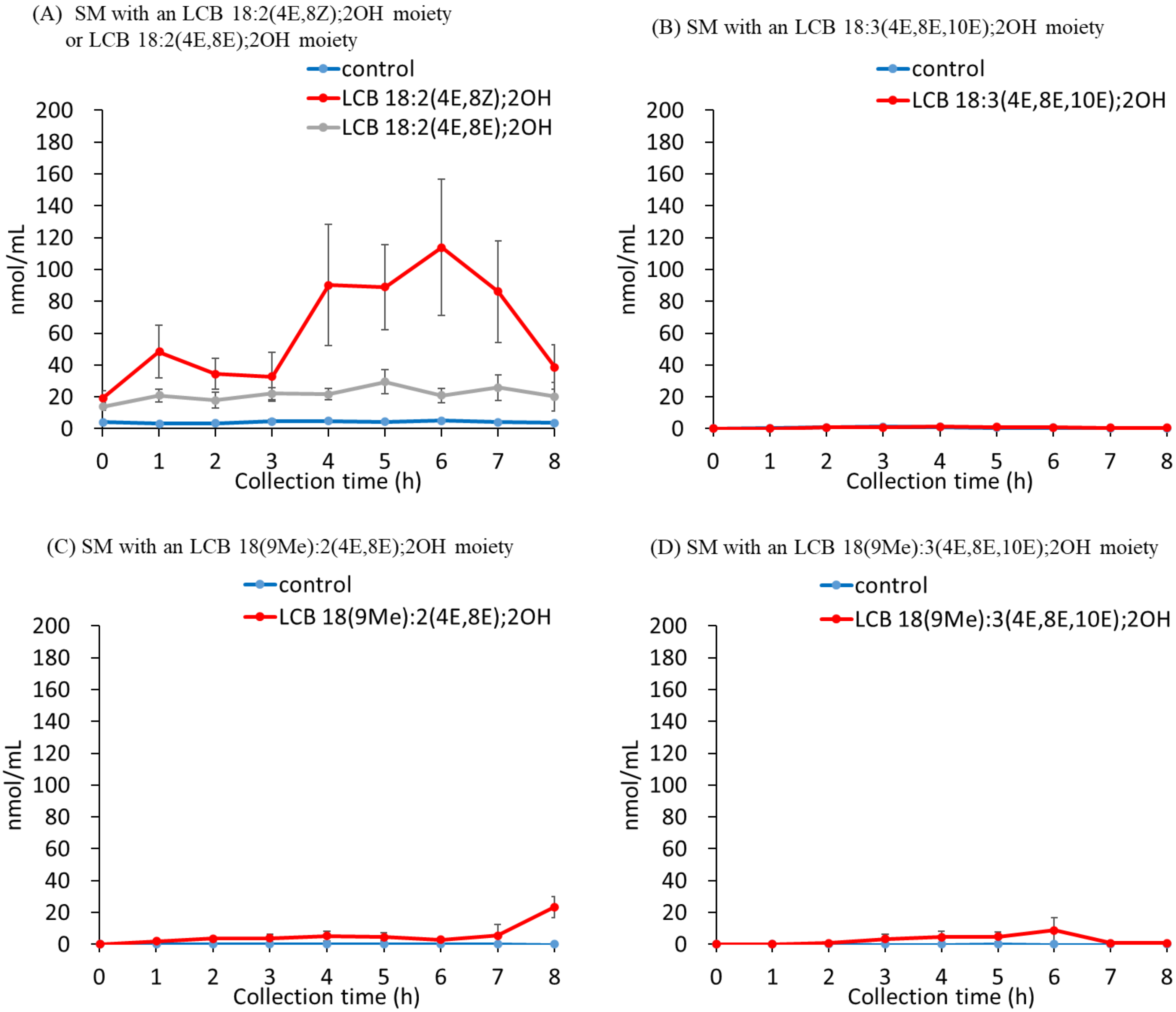

Figure S17: Molar concentrations of shingomyelins in chyle of rats after enteral administration of long-chain base emulsions. (A) SMs with an LCB 18:2(4E,8Z);2OH moiety or an LCB 18:2(4E,8E);2OH moiety, (B) SMs with an LCB 18:3(4E,8E,10E);2OH, (C) SMs with an LCB 18(9Me):2(4E,8E);2OH moiety, and (D) SMs with an LCB 18(9Me):3(4E,8E,10E);2OH moiety in the lymph of rats following enteral administration of long-chain base emulsions. Lipid amounts were normalized with collected chyle volumes. Values are presented as mean ± standard deviation (SD).
