# Supplementary material for: Structure-dependent absorption of atypical sphingoid long-chain bases from digestive tract into lymph
Source: Lipids Health Dis. 2021 Mar 1;20:24. doi: 10.1186/s12944-021-01448-2 (PMC7919070; doi:10.1186/s12944-021-01448-2)
Supplement: Supplementary file 2 — Additional file 2. Synthesis of Ceramides. [file 12944_2021_1448_MOESM2_ESM.pdf]

Supplemental information for "Structure-dependent absorption of atypical sphingoid long-chain bases from the digestive tract into lymph"

Additional file 2

Reagents

Palmitic anhydride, lignoceric acid, 1-Ethoxycarbonyl-2-ethoxy-1,2-dihydroquinoline (EEDQ) were purchased from Tokyo Chemical Industry Co., Ltd (Tokyo Japan).

Synthesis of Cer 18:2(4E,8);2OH/16:0

LCB 18:2(4E,8Z);2OH (8.3 mg, 27.9 μmol), palmitic anhydride (72.4 mg, 146 μmol) and methanol (5 mL) were mixed in glass capped vial and incubated at 50 ◦C for 31 h. After incubation reacted mixtures were filtrated through membrane filter (pore size; 0.45 mm) and purified with inertsil ODS-3 (diameter; 20 mm, length; 250 μm, particle size 5 mm. GL Science, Tokyo, Japan) column equipped HPLC. Cer 18:2(4E,8);2OH/16:0 was eluted with methanol at flow rate 4 mL/min. Eluted materials were monitored by UV absorbance at wave length 210 nm. The compound eluted at 48.677 min was collected and solvents were evaporated with rotary evaporator (Fig. S12A). Colorless amorphous solid (8.8 mg) was obtained and the purity was analyzed by HPLC equipped with CAPCELL PAK MG (diameter; 4.6 mm, length; 250 mm, particle size 5 μm. SHISEIDO, Tokyo, Japan). Cer 18:2(4E,8);2OH/16:0 was eluted with methanol at flow rate 0.8 mL/min and elution behavior was monitored by UV absorbance at wave length 210 nm (Fig. S12B,C). The structure was confirmed infusion ESI-MS/MS analysis.

Synthesis of Cer 18:2(4E,8);2OH/24:0

LCB 18:2(4E,8Z);2OH (30.3 mg, 102 μmol), lignoceric acid (5.6 mg, 145 μmol), EEDQ (80.8 mg, 326 μmol) and ethanol (5 mL) were mixed in glass capped vial and incubated at 50 ◦C for 31 h. After incubation, ethanol was evaporated with nitrogen stream and resulted materials were dissolved with 15 mL methanol/2-propanol=4:1 (vol/vol). Sample solution was filtrated through membrane filter (pore size; 0.45 μm) and purified with inertsil ODS-3 (diameter; 20 mm, length; 250 mm, particle size 5 μm) column equipped HPLC. The mixture of methanol/2-propanol=7:3 (vol/vol) was used as mobile phase at flow rate 4 mL/min and elution of Cer 18:2(4E,8);2OH/24:0 was monitored by UV absorbance at wave length 210 nm. The compound eluted at 51.311 min was collected and solvents were evaporated with rotary evaporator (Fig. S13A). Colorless amorphous solid (9.7 mg) was obtained and the purity was analyzed by HPLC equipped with CAPCELL PAK MG (diameter; 4.6 mm, length; 250 mm, particle size 5 μm. SHISEIDO, Tokyo, Japan). Cer 18:2(4E,8);2OH/24:0 was eluted with methanol/2-propanol=7:3 (vol/vol) at flow rate 0.8 mL/min and elution behavior was monitored by UV absorbance at wave length 210 nm (Fig. S13B,C). The structure was confirmed infusion ESI-MS/MS analysis.

MS analysis of Cer 18:2(4E,8);2OH/16:0 and Cer 18:2(4E,8);2OH/24:0

Purified Cers were dissolved with methanol at final concentration of 0.3 μM and TOF MS spectra and product ion spectra were recorded with TripleTOF5600 system. Sample solutions were directly injected TripleTOF5600 system and recorded TOF MS and product ion spectrum at positive ion mode. The parameters for TOF MS were as follows: ion spray voltage floating, 5500 V; temperature, 100° C; declustering potential, 80 V; collision energy, 10 V; ion source gas 1, 15 psi; ion source gas 2, 0 psi; curtain gas, 15 psi; accumulation time, 0.2 s. To record the production spectrum of Cer 18:2(4E,8);2OH/16:0 and Cer 18:2(4E,8);2OH/24:0, collision energy was changed to 35 V and *m/z* 536.5 and *m/z* 648.6 were selected as precursor ions, respectively (Fig S14. A-E).
